# Analysis of a gene panel for targeted sequencing of colorectal cancer samples

**DOI:** 10.18632/oncotarget.24138

**Published:** 2018-01-10

**Authors:** Klaus Højgaard Jensen, Jose M.G. Izarzugaza, Agnieszka Sierakowska Juncker, Rasmus Borup Hansen, Torben Frøstrup Hansen, Pascal Timshel, Thorarinn Blondal, Thomas Skøt Jensen, Eske Rygaard-Hjalsted, Peter Mouritzen, Michael Thorsen, Rasmus Wernersson, Henrik Bjørn Nielsen, Anders Jakobsen, Søren Brunak, Flemming Brandt Sørensen

**Affiliations:** ^1^ Department of Bio and Health Informatics, Technical University of Denmark, Kgs, Lyngby 2800, Denmark; ^2^ Intomics A/S, Kgs, Lyngby 2800, Denmark; ^3^ Oncology Department, Vejle Hospital, Vejle 7100, Denmark; ^4^ Exiqon A/S, Vedbaek 2950, Denmark; ^5^ Novo Nordisk Foundation Center for Protein Research, Faculty of Health and Medical Sciences, University of Copenhagen, Copenhagen DK-2200, Denmark; ^6^ Patologisk Institut, Aarhus Universitetshospital, Aarhus 8200, Denmark

**Keywords:** colorectal cancer, biomarker discovery, NGS, precision medicine

## Abstract

Colorectal cancer (CRC) is a leading cause of death worldwide. Surgical intervention is a successful treatment for stage I patients, whereas other more advanced cases may require adjuvant chemotherapy. The selection of effective adjuvant treatments remains, however, challenging. Accurate patient stratification is necessary for the identification of the subset of patients likely responding to treatment, while sparing others from pernicious treatment. Targeted sequencing approaches may help in this regard, enabling rapid genetic investigation, and at the same time easily applicable in routine diagnosis.

We propose a set of guidelines for the identification, including variant calling and filtering, of somatic mutations driving tumorigenesis in the absence of matched healthy tissue. We also discuss the inclusion criteria for the generation of our gene panel. Furthermore, we evaluate the prognostic impact of individual genes, using Cox regression models in the context of overall survival and disease-free survival. These analyses confirmed the role of commonly used biomarkers, and shed light on controversial genes such as *CYP2C8*.

Applying those guidelines, we created a novel gene panel to investigate the onset and progression of CRC in 273 patients. Our comprehensive biomarker set includes 266 genes that may play a role in the progression through the different stages of the disease. Tracing the developmental state of the tumour, and its resistances, is instrumental in patient stratification and reliable decision making in precision clinical practice.

## INTRODUCTION

### Colorectal cancer overview

Colorectal cancer (CRC) is one of the most common malignancies in the World; nearly 1.4 million new cases are diagnosed every year (WHO Globocan 2012). CRC is particularly prevalent in developed countries. For example, more than 5,000 new cases were recorded in 2014 in Denmark (Danish Colon Cancer Group, Annual Report 2014) with approximately two out of three being colon cancers (CC). Metastatic disease, synchronous or metachronous, will typically be detected in half of the patients. An overall 5-year survival of 60% underlines the need for improved treatment.

Surgery is the cornerstone in the treatment of patients with CRC and can be performed with curative intent in patients with stage I disease, which is restricted to the inner part of the bowel wall. At the other end of the spectrum, patients with metastatic disease, stage IV, are primarily treated with palliative chemotherapy and only a small fraction of them become long term survivors. The remaining patients, *i.e.* stage II disease growing to the outer layers of or through the bowel wall, and stage III involving regional lymph nodes, constitute a special clinical challenge. The majority is cured by surgery and an additional small fraction responds favourably to adjuvant chemotherapy. The challenge is to identify those patients who will benefit from adjuvant treatment and spare those who will not from toxic, unnecessary treatment. Therefore, new biomarkers to improve selection for adjuvant therapy are needed.

### Advances in precision medicine

The list of genes in which some mutations confer resistance to specific treatments has grown over the recent years and currently includes many examples. De Roock *et al.* [1] evaluated the role of *KRAS*, *BRAF, PIK3CA* and *PTEN* mutations in CRC and the efficacy of anti-EGFR therapy. Similarly, Modest *et al.* [2] investigated *KRAS* p.12 mutated CRCs. Dienstmann *et al.* [3] evaluated the improvement on the prediction of overall survival in the presence of microsatellite instability and considered *BRAF* V600E and *KRAS* mutations in a cohort of non-metastatic stage II and stage III CC patients. Keum *et al.* [4] proposed a panel for the stratification of CRC patients consisting on mutations in *KRAS, BRAF* and *PIK3CA* and the expression of *IRS1, IRS2, FASN*, and *CTNNB1* as these genes are implicated in the insulin signaling pathway. The American Society for Clinical Pathology, College of American Pathologists, Association for Molecular Pathology and American Society of Clinical Oncology have recently promulgated a series of guidelines for the evaluation of CRC [5]. The collection of biomarkers suggested in these *Good Practice Guidelines* includes: *KRAS* and *NRAS* codons 12 and 13 of exon 2, 59 and 61 of exon 3, 117 and 146 of exon 4; *BRAF* pV600, genes related to DNA mismatch repair status testing.

A steadily growing battery of precision medicine approaches based on mutations in these genes has increased the treatment possibilities for CRC patients over the last decade. These innovative therapies may be administered alone or as part of a combination protocol including also traditional therapeutic approaches. Additional targeted approaches attempt at bypassing the resistance to other treatments. This is the case of the resistance to EGFR-targeted therapies, developed by carriers of mutations in RAS proto-oncogenes [2].

All developments in the field led to current treatment of CRC being customised to the genome of the patient’s individual tumour by necessity. Tran and collaborators neatly reviewed the treatment alternatives for some of the aforementioned mutations [6].

### Summary of the work presented in this manuscript

We propose a novel panel of 266 genes that may be involved in onset and progression of CRC. Our panel is much more comprehensive than existing similar gene collections. and has been designed to include genes that are likely to play a causative role in the progression through the different developmental stages of the malignancy. Consequently, our panel may be instrumental in the decision making for precision clinical practice. We detail the steps taken for the selection of genes for our biomarker panel. First, we propose guidelines for the thorough filtering of germline variants in a setting, where matched healthy tissue is not available. This has a dual purpose: to reduce the analytical and computational requirements of the annotation of variants, facilitating an alignment with the throughput demands of a clinical setting, but also to ensure patient anonymity, a common requirement in precision medicine. Second, we propose guidelines for the inclusion of genes in a biomarker panel. Our integrative approach combines information from the automatic mining of the biomedical literature, different state-of-the-art databases (dbSNP, COSMIC, Uniprot, among others), and the largest cancer genomics effort to date [7]. The biomarker panel is also complemented with common targets for therapeutic drugs and genes deemed significant from our expertise on systems biology approaches. Third, we use Cox regression models to determine the role of specific genes in the survival of patients. This approach confirms the role of oncogenes recurrently involved in the onset and progression of CRC and contributes to the controversial role of *CYP2C8* as an active driver of cancer.

## RESULTS AND DISCUSSION

### NGS profile generation

### Cohort description

The patient cohort consisted of 273 CRC patients (137 males and 136 females) with complete metadata; 100 patients had been diagnosed with RC, while 173 were diagnosed with CC. Patients with CC present different laterality of their tumours, 101 and 72 patients had left- or right-side tumours, respectively. Further details on the composition of the cohort are included in Table 1 and Supplementary Figure 1.

**Table 1:** Description of the cohort after filtering

	Filtered retrospective cohort (*N* = 273)		Progression free survival (*N* = 217)	
Cancer type	CC	RC	CC	RC
Samples	173	100	136	81
Gender				
Male	81	56	65	43
Female	92	44	71	38
Stage				
I	10	12	10	6
II	78	34	67	31
III	63	43	55	39
IV	22	11	4	5
Tumour location				
Right sided	72	-	56	-
Left sided	101	-	80	-
Post-operative treatment				
None	139	94	104	78
Chemotherapy	33	3	32	3
Pre-operative treatment				
Radiation therapy	1	3	0	0
Age at time of operation				
Median	74	69.5	74	69
Mean	71.5	70.3	71.1	69.2

Our 273 patients were classified according to their disease stage. We found 22, 112, 106 and 33 in stage I, II, III and IV at time of operation, respectively. 217 had information on the time between surgery and eventual progression. The median age at time of operation was 73 years (69.5 for RC and 74 for CC).

### NGS data processing

Raw variant calling on 273 colorectal tumour samples resulted in a total of 152,520 variant positions, where at least one read covered an alternative allele. As no matched normal samples were available, the differentiation between germline and somatic variation turned up challenging. To circumvent this limitation, somatic variants of biological relevance were identified by technical and manual filtering. These cautiously applied filters removed known germline variants and those variants predicted to have only minor biological impact.

### Technical and biological filters

Applying technical filters (read depth greater or equal than 10 and fraction of alternative alleles (AAF) in the range between 0.05 and 0.95) reduced the total number of variants to 26,973. Of these, 4,454 variants were found in at least one of the genomic databases used for filtering, as described in Methods. Of the remaining 22,519 variants, 18,222 were identified as ‘modifier’ or ‘low impact’ by SnpEff [8].

### Manual filtering

Manual inspection of the variant table revealed some genes and positions that were clearly overrepresented due to a strange biological composition, *e.g. TBP* (6:170561916–17561960) and *LURAP1L* (9:12775850–12775885) contain G/S and Q-repeats, respectively, which are found in various lengths throughout a population, while *KRTAP4-5* consists of ~26 pentameric AA-repeats, although this number can vary between individuals. Moreover, *ERICH6B* (13:45596547–45596602) was masked due to in-frame deletions frequently occurring in European populations, albeit not identified in our automatic filtering steps. After masking out these four specific regions, attention was given to frequently occurring polymorphisms in dbSNP [9]. Histograms of the distribution of the alternative allele frequency for all rs-ids present in 4 or more samples are shown in the Supplementary File 2. Rs-ids or genomic positions, where the alternative allele frequency distributed evenly around 0.5, were considered of germline origin and removed from the dataset (100 rs-ids/positions). Also rs-ids, annotated as ‘Benign’ in ClinVar [10] and not having any connotations to cancer, were removed (58 rs-ids) and, finally, a subset of 13 rs-ids were removed for other reasons (high co-occurrence with other variants or updates made to dbSNP). As the extended panel aims at providing a general screening set for patients with CRC, rather than at explaining the contribution of rare variation, a final filtering step removed genes being mutated in fewer than 5 samples. After the manual filtering process, our ‘extended panel’ consisted of 3,841 high impact variants in 266 genes. These results are summarized in Supplementary Figure 3.

### Identification of most recurrently mutated genes

### Most frequently mutated genes

Table 2 displays the top 25 most frequently mutated genes in the 273 patients with CRC. Interestingly, 151 (54.9%), 143 (52%), and 73 (26.5%) of the patients present at least one somatic mutation in known tumour suppressor genes *TP53, APC* and *FAT4*, respectively. The commonly reported oncogene *KRAS* is also found in the CRC samples. Namely, 76 (27.6%) of the tumours present somatic mutations in this gene.

**Table 2:** Most frequently mutated genes

Gene	Sample count	Sample freq (%)	COSMIC freq. (%)	ICGC freq. (%)
*TP53*	151	54.9	43.5	57.6
*APC*	143	52	41.2	58.6
*SYNE1*	90	32.7	26.9	30.0
*KRAS*	76	27.6	34.7	35.4
*FAT4*	73	26.5	19.6	22.4
*LRP2*	71	25.8	17.5	12.4
*LRP1B*	61	22.2	20.4	18.6
*DNAH5*	61	22.2	17.0	18.9
*CSMD1*	59	21.5	13.3	14.3
*ATM*	57	20.7	22.7	12.4
*DMD*	49	17.8	3.5	10.5
*PCDHGA8*	45	16.4	5.6	24.9
*CSMD3*	45	16.4	21.2	17.8
*RYR2*	42	15.3	19.1	20.5
*PIK3CA*	42	15.3	13.5	19.2
*AXIN1*	41	14.9	3.3	−
*MPO*	40	14.5	2.7	−
*SLC22A1*	39	14.2	1.6	−
*ZNF208*	38	13.8	5.1	−
*BRAF*	38	13.8	12.3	10.5
*SCN10A*	36	13.1	6.2	−
*PCDHGB4*	36	13.1	4.3	27.3
*OR2L13*	36	13.1	1.9	7.6
*FBXW7*	36	13.1	11.4	13.8
*CES1*	36	13.1	2.3	−

The fact that *SYNE1* appears mutated in 90 (32.7%) tumours might be purely artefactual, given the extreme length of this gene, encoding for 8797 amino acids. In order to correct for the increased probability of finding longer genes among the recurrently mutated ones, we normalised the mutated counts with respect to the total nucleotide count of the genes (Table 3). As expected, *SYNE1* disappears from the ranking, while known cancer-relevant genes such as *KRAS*, *TP53* and *APC* prevail. *OR2L13*, an olfactory receptor gene, known to hypermutate in spite of its commonly assumed neutrality, ranks high in the corrected list. Moreover, the variants found in this gene are likely germline rather than somatic. The hypervariability of the gene makes it difficult for filtering approaches to discern this subclass of rapidly evolving gene families and to filter germline variation satisfactorily.

**Table 3:** Most frequently mutated genes, corrected by gene length

Gene	Weighted mutation frequency
*KRAS*	0.42
*TP53*	0.41
*OR2L13*	0.12
*APC*	0.08
*SKC22A1*	0.08
*CES1*	0.07
*FBXW7*	0.07
*SUPT4H1*	0.06
*B2M*	0.06
*PCDHGA8*	0.06
*MP0*	0.06
*NRAS*	0.05
*BRAF*	0.05
*AXIN1*	0.05
*PIK3CA*	0.05
*MT1A*	0.05
*HIST1H4F*	0.05
***CYP2C8***	**0.05**
*SH3BGRL3*	0.04
*PCDHGB4*	0.04
*CYP3A5*	0.04
*CYP2B6*	0.04
*ZNF208*	0.04
*NR1H4*	0.04
*TCF7L2*	0.04

### Most frequently mutated local regions

Mutations in cancer genes, especially oncogenes, tend to group around particular positions of the protein [11]. A sign that these regions retain functional relevance, when mutated, confers an adaptive advantage to cancer cells, - a proxy for the identification of novel candidate driver genes. To isolate regions of the genes that accumulate mutations, we characterised the mutation burden *per* exon as a proxy for functional unit (Table 4). The last exon (exon 16) of APC is the one accumulating the most mutations, as 120 patients carry a mutation in this exon. Followed by exon 2 in *KRAS* (*n* = 63) and exon 8 in *TP53* (*n* = 47). These are known cancer-related genes previously discussed. A runner-up in this classification is the first exon of the protocadherin gamma subfamily A 8 gene, *PCDHGA8*, which appears mutated in 44 of our CRC patients. This gene has not been previously associated to CRC.

**Table 4:** Most frequently mutated exons

Gene	Exon rank	Sample count
*APC*	16/16	120
*KRAS*	2/6	63
*TP53*	8/11	47
*PCDHGA8*	1/4	44
*ZNF208*	1/4	38
*OR2L13*	2/2	36
*PCDHGB4*	1/4	35
*TP53*	5/11	32
*BRAF*	15/18	32
*PCDHA10*	1/4	31
*FAT4*	9/17	29
*FAT4*	1/17	29
*TP53*	7/11	27
*TSHZ3*	2/2	27
*SLC22A1*	7/11	26
*AXIN1*	7/11	26
*PCDHGA2*	1/4	24
*TP53*	6/11	23
*PCDHA5*	1/4	22
*PCDHA3*	1/4	22
*PCDHGA4*	1/4	21
*PCDH2A2*	1/4	21
*AMER1*	2/2	20
*FAT4*	17/17	20
*PCDHA8*	1/4	19

Furthermore, we explored, whether mutations were distributed across the entire exon or targeted a limited number of preferred amino acids. The latter would help relate the pernicious effect with a functional role for the affected amino acid. Several positions stand out in this analysis (Table 5). Examples are position 12 of *KRAS* (*n* = 47), mutation of amino acid 600 in *BRAF* (*n* = 29), or the accumulation of somatic mutations in position 273 of *TP53* (*n* = 20). Interesting from this analysis is the incorporation of a frequently mutated (*n* = 25) position 650 in the *AXIN1* gene. This gene is a regulator of apoptosis via induction of the WNT pathway, and consequently, likely driving cancer aetiology in the affected patients.

**Table 5:** Most frequently mutated gene positions

Gene	Pos (AA)	Sample count	% samples mutated in gene
*KRAS*	12	47	61.8
*BRAF*	600	29	76.3
*AXIN1*	650	25	61.0
*OR2L13*	265	24	66.7
*TP53*	273	20	13.2
*PCDHGA8*	770	18	40.0
*SLC22A1*	425	15	38.5
*MPO*	332	15	37.5
*TP53*	175	15	9.9
*PCDHGB4*	420	14	38.9
*KRAS*	13	14	18.4
*PCDHA10*	81	13	39.4
*PIK3CA*	1047	11	26.2
*APC*	876	11	7.7
*PMS2*	597	11	42.3
*CES1*	285	10	27.8
*TP53*	248	10	6.6
***CYP2C8***	**181**	**10**	**47.6**
*FAT4*	4726	9	12.3
*GGT1*	372	9	50.0
*TP53*	282	9	6.0
*TOP1*	81	9	64.3
*SLC22A1*	420	8	20.5
*CES1*	144	8	22.2
*FCGR3B*	114	8	80.0

### Most frequently mutated OncodriveCLUST spatial clusters

An extension of the previous approach is the consideration of spatial clusters instead of individual positions as a proxy for functional hot-spots. OncodriveCLUST [12] is a method to identify grouping of mutations positively selected during clonal evolution of tumours. One main strength of the method is that a homogeneous baseline mutation probability across all genes is not assumed, as this is likely an oversimplication. In contrast, OncodriveCLUST creates a background model, using silent mutations, which are supposed to be under no positive selection and may reflect the baseline mutability of different positions across the gene. OncodriveCLUST was run, using default parameters and the results displayed in Table 6. The clusters identified vary in length and include recurrent individual positions. As expected, the highly ranking genes previously identified (*KRAS, BRAF, TP53, APC, AXIN1,* …) also rank high in this method. However, it provides a finer grain definition of the hot-spots. This is the case of *KRAS* 12-14, mutated in 62 patients (respect to the 47 identified before on position 12).

**Table 6:** Most frequently mutated gene intervals, as defined by OncodriveClust

Gene	Interval	Sample count	% of samples mutated in gene
*KRAS*	12–14	62	81.6
*BRAF*	600	29	76.3
*TP53*	266–277	27	17.9
*TP53*	235–251	25	16.6
*AXIN1*	650	25	61.0
*TP53*	161–180	24	15.9
*SLC22A1*	419–425	24	61.5
*OR2L13*	265	24	66.7
*PCDHGA8*	770	18	40.0
*APC*	1303–1322	16	11.2
*MPO*	332	15	37.5
*PIK3CA*	542–549	14	33.3
*PCDHGB4*	420	14	38.9
*PCDHA10*	81	13	39.4
*PMS2*	595–597	12	46.2
*TP53*	282–296	12	7.9
*PIK3CA*	1043–1047	12	28.6
*APC*	876	11	7.7
*APC*	213–216	11	7.7
*TP53*	190–197	11	7.3
*PCBP1*	100–102	10	100.00
***CYP2C8***	**181**	**10**	**47.6**
*CES1*	285	10	27.8
*TOP1*	80–81	10	71.4
*APC*	1404–1415	9	6.3

### Correlation to outcome/other metadata

Regions identified by OncodriveClust, enriched for mutations, were included in a Cox regression analysis to find genomic areas that are negatively affecting overall survival (OS) and/or progression free survival (PFS).

No mutated genes were found to be significant predictors of OS or PFS for RC patients, neither when using all genes with mutations for the regression model, nor when only looking at genes mutated in at least five samples.

**Figure 1:**
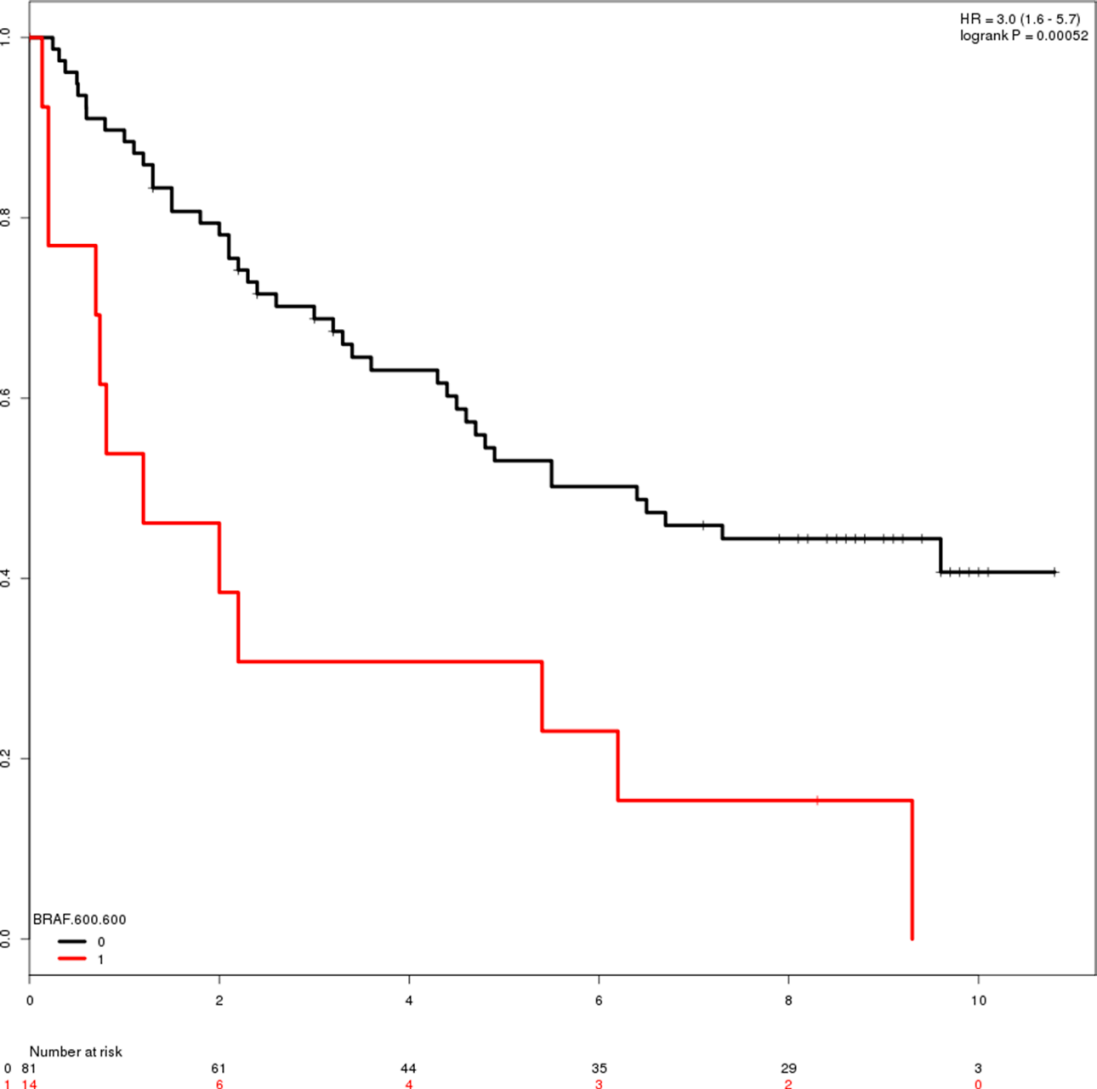

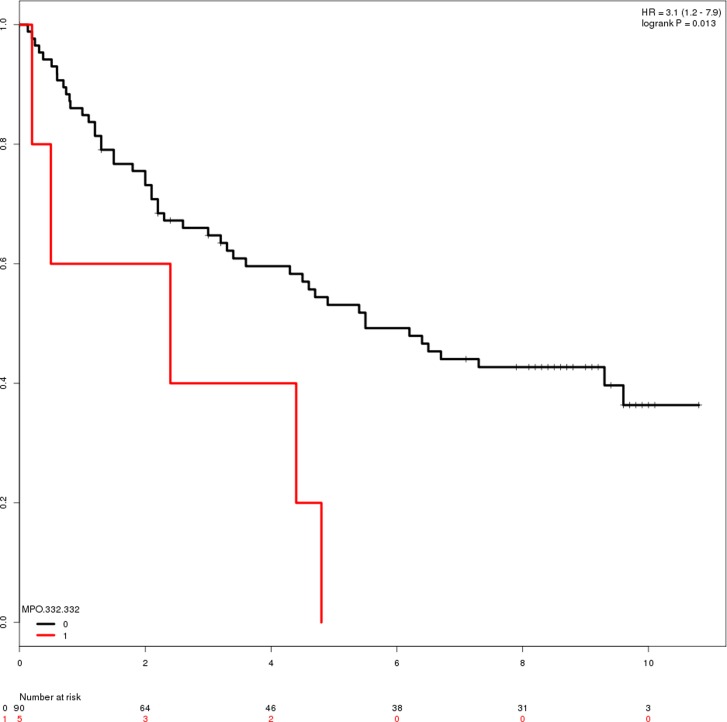
Overlap between the minor allele frequency (MAF) databases dbSNP, ExAC and GenomeDK used in the project

The pooled cohort of CC and RC patients reveals six regions with consistent non-zero coefficients in the Cox regression analysis. Table 7 reports the regions and log rank *p*-values of hazard ratios. Four clusters/positions were found to be significant predictors of reduced survival time, when reducing covariate space to include only clusters identified in cluster analysis. *BRAF* 600 and *MPO* 332 were found to be mutated in 11.1% (*n* = 24) and 4.6% (*n* = 10) of CC samples, respectively. Similarly, *CYP2B* 181 (*n* = 8, 3.7%) and *TP53* 305:307 (*n* = 8, 3.7%). Although several genes and clusters are mutated much more frequently than these two, they are not found to be significant prognostic markers (Figure 1A and 1B, respectively). A total of 47 (21.7%) individual patients present mutations in one or more of these regions.

**Table 7:** Intervals with non-zero coefficients in Cox-regression on pooled cohort

Gene	Overall survival					Progression free survival			
	Stage	I-III		I-IV		I-III		I-IV	
	Interval	*p*-value	HR	*p*-value	HR	*p*-value	HR	*p*-value	HR
*BRAF*	600:600	9.5e-3	2.0	1.2e-3^*^	2.0	4.6e-3^*^	2.1	1.2e-3^*^	2.2
*CYP2C8*	181:181	6.0e-3^*^	2.6	1.4e-2	2.3	5.0e-3^*^	2.7	9.2e-3	2.5
*MPO*	332:332	8.4e-4^*^	2.9	6.5e-4^*^	2.6	4.7e-3^*^	2.6	8.4e-3	2.4
*NR1H4*	183:189	8.2e-2	2.0	3.0e-2	2.2	4.4e-2	2.3	2.7e-2	2.5
*TP53*	235:251	9.5e-2	1.66	1.2e-1	1.54	6.6e-2	1.74	5.1e-2	1.76
*TP53*	305:307	2.6e-3^*^	3.1	5.3e-3^*^	2.7	3.5e-3^*^	3.0	1.1e-3^*^	3.1

OS and PFS are reported as independent analyses. 4 regions are predicted to have a significant negative impact on PFS after Bonferroni correction for multiple testing. [Significant Bonferroni corrected *p*-value: 8.3e-3 (*n* = 6)].

The role of *BRAF* and *TP53* as (proto)oncogenes is widely described in the literature. Contrarily, *CYP2C8* (Cytochrome P450 2C8) and *MPO* (Myeloperoxidase) is less established. *CYP2C8* is involved in the metabolism of several common drugs, and have been related to reduced metabolism of paclitaxel in *in vitro* studies [13], but the same effect has not been shown *in vivo*. Contrarily, several polymorphisms in *CYP2C8* have previously been investigated for potentially protective roles in CRC, but with negative results [14].

*MPO* is a widely used marker for inflammatory bowel disease [15], being an enzyme playing a central part in the host defense system and a well-known biomarker for chronic inflammation of the large intestine. Mutations in *MPO* 332 have previously been reported to increase hazard ratio for acute lymphoblastic leukemia patients [16], but have not been reported in relation to CRC. Position 332 is located in close proximity to a metal binding site (positions 334, 336, 338 and 340), and thus one might hypothesize that this mutation is changing the enzymatic activity of *MPO*.

### Progression free survival of CC patients in stage I-III

Similarly, a PFS analysis was carried out for CC and RC patients separately (Table 8). Three positions/regions (*BRAF* 600, *MPO* 332 and *TP53* 305:307) were found to significantly impact PFS for CC patients, while none were found in the RC cohort.

**Table 8:** Intervals with non-zero coefficients in Cox-regression on colon cancer and rectal cancer patients, respectively

		Progression free survival							
	Location	Colon cancer				Rectal cancer			
	Stage	I-III		I-IV		I-III		I-IV	
**Gene**	**Interval**	*p*-value	HR	*p*-value	HR	*p*-value	HR	*p*-value	HR
*BRAF*	600:600	6.4e-5^*^	3.0	3.3e-5^*^	2.9	0.2	0	0.56	0.56
*CYP2C8*	181:181	3.7e-2	2.5	4.7e-2	2.44	7.7e-2	2.87	0.11	2.54
*MPO*	332:332	2.3e-3^*^	3.8	3.8e-3^*^	3.6	0.23	1.87	0.29	1.73
*NR1H4*	183:189	4.8e-2	3.05	6.2e-2	2.9	0.33	1.78	0.13	2.16
*TP53*	235:251	0.28	1.57	0.34	1.5	0.12	1.96	0.078	2.05
*TP53*	305:307	2.8e-3^*^	3.7	4.2e-4^*^	4.1	0.39	1.85	0.45	1.71
*APC*	1303:1322	8.1e-2	2.1	0.11	1.97	0.37	1.6	0.48	1.44

Three positions/intervals which were also identified in the pooled analysis are significant after Bonferroni correction for multiple testing [Significant Bonferroni corrected *p*-value: 7.14e-3 (*n* = 7)].

These three genes were also responsible for determining the overall survival of colon cancer patients in stages I-III as represented in Table 9.

**Table 9:** Intervals with non-zero coefficients in Cox-regression on colon

	Overall survival		
	Location	Colon cancer	
	Stage	I-III	
**Gene**	**Interval**	*p*-value	HR
*BRAF*	600:600	9.1e-6^*^	2.8
*CYP2C8*	181:181	0.13	1.99
*MPO*	332:332	1.0e-3^*^	3.2
*TP53*	305:307	0.22	0.5
*PCDHBG4*	420:420	3.7e-3^*^	3.2

Three positions/intervals are significant after Bonferroni correction for multiple testing [Significant Bonferroni corrected *p*-value: 0.01 (*n* = 5)].

## CONCLUSIONS

The future of CRC treatment goes through patient stratification and precision treatment, customised to the individual genome of the patient’s tumour. Here we proposed a novel model for creating a biomarker panel, to facilitate decision making in a clinical scenario. Targeted sequencing of a defined number of biomarkers remarkably alleviates the economic and technological pitfalls of analysing a country wide cohort to a point scalable and feasible for clinical practice. We suggest guidelines for the filtering of germline variants to facilitate analysis in alignment with throughput demands of a clinical setting and to ensure the often required patient anonymity. We propose a detailed protocol for the inclusion of genes into the biomarker panel; we integrate information extracted from biomedical literature with forefront text mining approaches; we analyse pathways to identify candidate genes, and we address the common therapeutic targets of commonly administered therapies. These systems biology approaches complement the information extracted directly from dedicated state-of-the-art databases (among others: dbSNP, COSMIC, Uniprot), and the largest cancer genomics effort to date [7]. After filtering and validation, our panel consists of 266 genes. Finally, Cox regression models determine the contribution of specific genes in the survival of patients with CRC. With this approach, known oncogenes recurrently involved in the onset and progression of CRC are confirmed, and a new candidate oncogene, whose role has been a matter of recent debate, *CYP2C8*, is proposed as an active driver of cancer.

## MATERIALS AND METHODS

### Description of cohorts

The retrospective study population consisted of 303 patients, who underwent surgical resection of histologically verified adenocarcinomas of the colon or rectum at the Departments of Surgery, Horsens, Kolding and Vejle Hospitals, Denmark, from January 1999 through December 2000, and from whom archival tumour tissue was available. Patients in our cohort presented with disease stages ranging from I to IV, as displayed in Table 1 and Supplementary Figure 1. After filtering, our cohort consists of 273 patients with CRC.

Patients dying because of either post-operative complications or within one month from the operation (*N* = 24) were not included. Similarly, patients receiving neoadjuvant therapy prior to the primary intervention (*N* = 4) were excluded from the survival analysis. Reliable overall survival (OS) and progression-free survival (PFS) information was available for 217 patients with CRC (136 CC, 81 RC) including 9 stage IV patients (4 CC, 5 RC) at the time of surgery.

Pre-treatment examinations included a chest x-ray and ultrasound or CT scan of the abdomen. Postoperatively, tumours were histologically classified and staged according to the pTNM system [17]. Information regarding patient characteristics, relapse status and survival were based on patient records and registries. The study was reported to the Danish Data Protection Agency of Southern Denmark (ID#: 2008-58-0035) and approved by the Regional Scientific Ethical Committee for Southern Denmark, according to Danish law (ID#: S-20150010). The Danish Registry of Human Tissue Utilisation allows registered Danish citizens to refrain from scientific research, and all the studied patients were confirmed not to be included in this registry.

### Tissue specimens and tissue processing

All tumour containing tissue blocks were retrieved from the archives at the Department of Clinical Pathology, Vejle Hospital, Denmark, where all specimens originally had been processed, using standardized procedures for diagnostic purposes. In brief, the surgical specimens had been routinely fixed in formaldehyde over night, and 1 through 6 tissue blocks from each tumour had been dehydrated and embedded in paraffin. One 4 μm thick, hematoxylin-eosin (HE) stained tissue slide was cut from each tissue block and reviewed by an experienced pathologist for tumour content (*i.e.,* tumour cell nuclei) in steps of 10%. Tissue blocks with a tumour nuclear fraction, subjectively estimated to be lesser than 30%, were excluded from the study, resulting in the inclusion of 1 through 3 tissue blocks from each surgical specimen.

When cutting the tissue sections, care was taken to avoid contaminating tumour tissue from one case to another. Thus, cleaning of the working area was undertaken after cutting each case. Moreover, the technician changed gloves, replaced the knife on the microtome, and cleaned the microtome after finishing cutting the tissue blocks from individual cases. Tissue sections were placed in microtubes (MCT-150-C; 1.5 ml RNase/DNase/pyrogen safe; Axygen, USA), and transported to Exiqon A/S for further processing.

### Preparation of tumour tissue

In an initial prototype phase, five tumours were selected from the cohort to study the practical handling of the specimens, such as the DNA extraction as a function of the degree of infiltration of inflammatory cells in the tumour tissue, and the influence of intra-tumoural heterogeneity. These issues are of outmost concern for the clinical applicability of the laboratory technique. Tumours were selected so that each would have 4 blocks of tumour tissue available. Two 15 μm thick tissue sections were cut from each of the 4 tissue blocks available from each case, and placed in separate microtubes. Cleaning of the working area, change of gloves and microtome knife, as well as cleaning of the microtome, were carried out after cutting each block from individual cases. Moreover, two additional 15 μm thick tissue sections were cut from each of the same tissue blocks, and mutually placed in one microtube *per* case (Supplementary Table 1). All sections were cut adjacently, with one additional HE-stained section at the top and the bottom of the tissue section stack, to ensure the content of tumour tissue. On these two latter tissue sections, the content of inflammatory cells was estimated semi-quantitatively by an experienced pathologist (score: *low* or *high*). Also, the tumour cell fraction (*i.e.,* the tumour nuclear fraction) was estimated subjectively in the same session, as mentioned above.

A subsequent phase considered the remaining tumours. Tissue availability imposed some restrictions. For example, when screening HE-stained sections from the individual tumours of all 273 patients, the threshold of 30% tumour content had diminished the number of formalin-fixed, paraffin-embedded (FFPE) tissue blocks to 1 through 3 for each case. Within these tissue blocks it was, however, evident that some cases had rather low tumour content. Thus, the individual cases were divided into tumours of low (*N* = 30) and high fraction (*N* = 243) of malignant tumour cells.

Cutting tissue sections from these FFPE tissue blocks for the discovery study was modified according to the knowledge obtained in the prototype phase. Thus, the efficiency of the enzymatic digestion of the tissue slides was improved by using 10 μm thick tissue sections. Moreover, the intra-tumoural heterogeneity did not profoundly influence the results obtained (Supplementary Figure 2). Accordingly, a total of six 10 μm thick tissue sections were cut from each FFPE tissue block; *i.e.,* in the case of only one FFPE tissue block *per* patient, all sections were cut from this block, whereas in the case of 2 or 3 FFPE tissue blocks *per* patient, 3 or 2 tissue sections were cut from the individual tissue blocks, respectively. All 6 tissue sections from each patient were mutually placed in microtubes, using the working setting stated above. For quality control, one 4 μm thick, HE-stained tissue section was cut from each FFPE tissue block, after cutting the adjacent sections, mentioned above, to ensure the tumour content (≤30% or >30% adenocarcinoma cell nuclear fraction) of the individual cases.

### Construction of the target gene list

### Selection of frequently mutated genes

To identify relevant gene targets for sequencing, we selected a panel of gene candidates based on literature reviews and publicly available databases (Supplementary Table 3). All gene names were mapped HGNC identifiers, using the R package HGNChelper before merging into a combined gene matrix. A total of 1426 unique genes were scored based on nine features (Supplementary Table 4) encompassing information about their mutation frequency and known association with CRC. The score was computed for each gene by summing the number of occurrences of the gene across all nine features. Genes were then ranked based on their score (ties were resolved by selecting the highest CRC mutation frequency as listed by COSMIC). As there is overlap among the selection criteria defined in Supplementary Table 4, the scoring algorithm resulted in 93 genes with a positive score (score ≥0). Six additional genes with a mutation frequency greater than 7% were added to the gene panel, giving a total of 99 gene targets.

### Extension of gene panel

In a subsequent step, the aforementioned set of genes was extended to include additional genes in which mutations likely play a significant role in the aetiology of CRC. Several gene lists were constructed, including: a) Genes found to be co-mentioned with CRC in PubMed abstracts, b) genes from selected pathways, c) genes with proteins targeted by compounds in relevant oncologic treatments, d) genes that are often mutated in CRC according to our analysis of data from The Cancer Genome Atlas (TCGA) [7], and e) genes that were hand-picked based on expert knowledge. The content of these lists will be disclosed in the following sections.

### Genes from text mining

Using Intomics’ database of synonyms for diseases and genes, we text mined a corpus consisting of 13,417,371 abstracts from PubMed dating from before September 2013. A total of 53,930 abstracts mentioned CRC or one of its synonyms, and for each gene a Fisher’s exact test assessed, whether synonyms for the gene were mentioned together with synonyms for CRC more often than would be expected by random. After adjustment for multiple testing at a Bonferroni-corrected significance of 5·10^−7^, 375 genes significantly associated with CRC, according to the text mining.

### Genes from pathways

A list was constructed consisting of genes from the KEGG pathways [18] “hsa05210” (CRC), “hsa04370” (VEGF signalling pathway), and genes with proteins annotated in UniProt [19] with GO-accession [20] “GO:0048010” (VEGF receptor signaling pathway) or descending accessions. This list was then filtered as described below.

### Genes as targets related to therapy

Protein targets for the compounds bevacizumab, capecitabine, cetuximab, fluorouracil (5-FU), irinotecan, oxaliplatin and panitumumab, all of which are used in treatment of CRC, were extracted from DrugBank [21], and the corresponding genes were added to a list. In addition, genes corresponding to protein targets for the above compounds and floxuridine, regorafenib, sorafinib, sunitinib and vatalanib, also used in therapy of CRC, were extracted from the CHEMBL database [22], filtered for biological relevance as described below, and added to the list.

### Genes from TCGA data

Data for somatic mutations in patients with adenocarcinoma of the colon or rectum were downloaded from The Cancer Genome Atlas [7] in December 2013. Low impact mutations according to SNPeff [8] were discarded, and for each gene the number of remaining mutations was normalised taking gene length into consideration. The 50 genes with the highest mutation rate were then used for further analysis.

### Hand-picked genes

*EGFR, EPCAM, MLH3* and *PMS2* are human genes where the corresponding proteins are annotated with the “Hereditary non-polyposis CRC” keyword in *UniProt* [19]. These were added to the panel.

### Filter of biologically relevant genes

The different gene lists discussed above were filtered according to their biological relevance before they were included in the panel.

The protein-protein interaction network InBio Map^TM^ developed by Intomics [23] was used to identify genes, whose proteins interact with the proteins coded by the 50 genes that had the highest mutation rates in the data from TCGA. The rationale behind the filtering is that if a mutation in gene *A* is relevant, then mutations in gene *B* may also be relevant, given that both their protein products are part of the same protein complex. A gene was included on this list if it was one of the 50 genes with the highest mutation rates, or if at least 10% of its corresponding protein’s known interactors were among the proteins coded by these 50 genes (only high-confidence interactors were considered).

### Construction of genomic libraries

### DNA isolation

DNA was isolated from six 10 μm slices of formalin-fixed, paraffin-embedded (FFPE) tissues using the QIAamp DNA FFPE Tissue kit (Qiagen Inc.) with the following amendments: Samples were deparaffinised four times with 1 mL xylene (Sigma Inc.). Digestion steps were performed in double volumes, in that protease K digestion was performed in 360 μL ATL buffer, using 40 μL Protease K overnight at 65° C, followed by a heating step of 75° C for 15 min. RNA digestion was carried out, using 4 μL RNase A, then 400 μL AL buffer was added and 400 μL ethanol (100%), vortexed and loaded into columns in two steps. DNA was eluted in 75 μL nuclease-free water and quantified using a Nanodrop 1000 spectrophotometer (Thermo Scientific).

### Fractionation

Samples were fractionated, using M220 Focused-Ultrasonicator (Covaris Inc.), aiming at average size of 200 bp. An aliquot of 3 μg of genomic DNA was diluted to a final volume of 130 μL and transferred to a microTUBE AFA Fiber Snap-Cap tube, using the following settings at 4° C: Peak incident power 50, duty factor 20%, cycles per burst 200, treatment time 300 seconds. Samples were purified and concentrated, using AMPureXP magnetic bead system (1.8x volume beads), washed twice in 70% ethanol (Agencourt Bioscience Corporation), and eluted in nuclease-free water. The quality of the fractionation was checked on a bioanalyzer, using DNA High Sensitivity chips.

### Genomic library construction

Library construction and target gene enrichment were performed using the SureSelect XT Target Enrichment system (Agilent technologies Inc.), according to the manufacturer’s instructions based on a published protocol [24]. In short, the fractionated genomic DNA (3 μg) was end-repaired, 3′ dA overhangs added followed by adapter ligation. Between the three library generation steps, the samples were purified and concentrated, using AMPureXP bead system (1.8x volume beads), washed twice in 70% ethanol (Agencourt Bioscience Corporation) and eluted in nuclease-free water. The libraries were finally amplified (12 cycle protocol), using Hercules II fusion PCR system (Agilent Inc.) and purified again with AMPureXP bead system. Libraries were quantified, using Nanodrop 1000 (Thermo Fisher Inc.).

### Targeted sequencing

### Biotinylated RNA baits

In the initial pilot phase, commercially available SureSelect XT Human-All-Exome RNA baits (Agilent Technologies Inc.) were used following the instruction from the manufacturer.

The retrospective cohort was interrogated, using a novel panel of 266 genes. These genes were uploaded into the Agilent SureSelect E-array software to design 56,008 probes (3× tiling density), using moderately stringent masking. The probes covered exons (± 10 nt.), 3′ UTRs and 5′ UTRs of the 266 candidate genes under investigation. Total genomic region spanned 1.357 Mbp. The sequences of all 56,008 baits are listed in the Supplementary File 1. Biotinylated RNA baits were synthesised by Agilent Inc. for the SureSelect XT Target Enrichment system.

### Pooling and hybrid capture

DNA libraries (750 ng) were transferred to 1.5-mL polypropylene sample tubes, lyophilized with a speedvac evaporator, and resuspended in 3.5 μL of nuclease-free water. Solution-based hybrid capture was performed according to the SureSelect XT protocol with overnight hybridization at 65° C, standard washing and Dynabead purification (Thermo Fisher Inc.) according to the manufacturer’s instructions. Libraries were amplified (12 cycle protocol) with Hercules II Fusion PCR system and SureSelect 96 index system, using half of the enriched library (bound to Dynabeads) and quality checked on Bioanalyzer DNA High Sensitivity chip (Agilent Technologies Inc.). Then libraries were diluted to 1:40,000 and quantified by KAPA qPCR system for Illumina libraries (KAPA biosystems Inc.), using library standards according to manufacturer’s instructions on a LightCycler 480 qPCR system (Roche), and the second derivative MAX Cq calculation method. Libraries were run on a HiSeq 2500 sequencer (Illumina Inc.), using version 3 chemistry and a 75 bp paired end protocol. After sequencing, BCL data files were de-multiplexed and converted into FASTQ data, using bcl2fastq software (Illumina Inc.).

### Heterogeneity analysis on prototype phase samples

Inter- and intra-sample heterogeneity of the 5 samples from the prototype phase was assessed by comparing the mutation profile between samples from different tumours and slices from the same tumour.

For each slice, the overall sequencing quality was assessed using *fastqc v0.11.2*. Sequences were trimmed and adapters and low quality sequences were removed using *cutadapt v.1.2.1* and *prinseq-lite v0.20.4* to improve mapping to the human reference genome.

Reads were mapped to the hg19-build of human reference to ensure compatibility with BED-regions provided for WXS. A common artefact, when sequencing FFPE samples, is a high proportion of duplicate reads. Those were marked and removed before further processing using *Picard-tools - MarkDuplicates v1.109.*

The resulting files from the mapping were subset to contain only reads mapping to gene regions specified in Agilent SureSelect WXS kit with *samtools v0.1.18.*

After preprocessing of the samples, a thorough analysis was carried out to identify mutations in a range of genes and to investigate both inter- and intra-sample heterogeneity based on the mutation profiles of the specimens. Thus, single nucleotide polymorphisms (SNP) and indels were called with *VarScan v2.3.7*. Identified SNPs and indels were filtered with dbSNP138 [17] and annotated with *Annovar v2.0*.

### Variant calling on discovery cohort

To assess the intra- and inter-sample heterogeneity the overlap between individual slices and samples was analysed with *Varscan v2.3.*7 and custom made scripts.

Sequencing read quality was assessed with *fastQC v.0.11.2.* To ensure high reads quality before mapping, low quality reads were removed, and adapter and low quality bases clipped from 3′ and 5′ using *cutadapt v.1.8.1* and *prinseq-lite.pl v.0.20.4.*

Reads were mapped to GRCh38, using *bwa mem v.07.12* [25]. Duplicate reads were marked (*Picard-tools 1.128*) [26], and only reads mapping in proper pairs were selected for variant calling.

Variant calling was performed with *bcftools v.1.2,* utilizing the multi-allelic caller to ensure conservation of information on multi-allelic sites. Indels were left-aligned, and multi-allelic sites were split into bi-allelic records, which were then annotated with information from dbSNP and the Danish Reference Genome [27, 28]. Variant effects were evaluated for canonical transcripts with *snpEff v.4.1l* [8], using genome version GRCh38.76.

Several criteria were used to identify somatic variants of biological relevance. Allele frequencies were defined as the number of high quality bases supporting the allele divided by the total number of high quality bases. We used the same definition of “high quality” as in the *samtools software package* [29]. In each sample, the variant positions were flagged according to these criteria:

- If the QUAL column in the VCF file is below 20, the “LQ” (low quality) flag is used.
- If the number of high quality bases per genomic position is below 10, the “LD” (low depth) flag is used.
- If one alternative allele frequency is more than 95%, or if the number of high quality non-alternative alleles is below 5, the “HF” (high frequency) flag is used, if 5 or more high quality reads support the alternative allele. Since the tumour contents of the samples are expected to be much lower than 95%, somatic mutations will not have alternative allele frequencies that are so high.
- If all alternative allele frequencies at the position are below 5% or supported by less than 5 high quality bases, the “LF” (low frequency) flag is used. Somatic mutations that are driver mutations will not have such low allele frequencies, unless the tumour content of the sample is very low.
- If at least one alternative allele has a frequency of at 5% or more and is supported by 5 or more high quality reads, the “G5”, “DK”, “EG”, and “EE” flags are used, if, respectively, *all* such alleles have been observed in at least 5% of
  - a population in the 1000 Genomes Project [30] (the same as in the *dbSNP database* [9]),
  - the population in the Danish Reference Genome project [27, 28],
  - the population in the Exome Aggregation Consortium [31], and the European sub-population in the Exome Aggregation Consortium.
- If at least one alternative allele has a frequency of 5% or more and is supported by five or more high quality reads, and all such alleles have been observed either in at least 5% of *any* of the aforementioned populations, the “CO” flag (common) is used. Note the “CO” flag can be set for a position, even though none of the “G5”, “DK”, “EG”, or “EE” flags are set, if several alternative alleles are seen at the position, and one allele has one of the flags and another allele has another of the flags.
- Each alternative allele’s biological impact on canonical transcripts was assessed using the *snpEff software package*. The software assesses the level of impact, using the categories “modifier”, “low”, “moderate”, and “high”. The maximum impact level for the alternative alleles with frequencies of 5% or more, and supported by 5 or more high quality reads, was computed and represented by the flags “I0”, “I1”, “I2”, and “I3” corresponding to the four impact levels.

**Figure 2:**
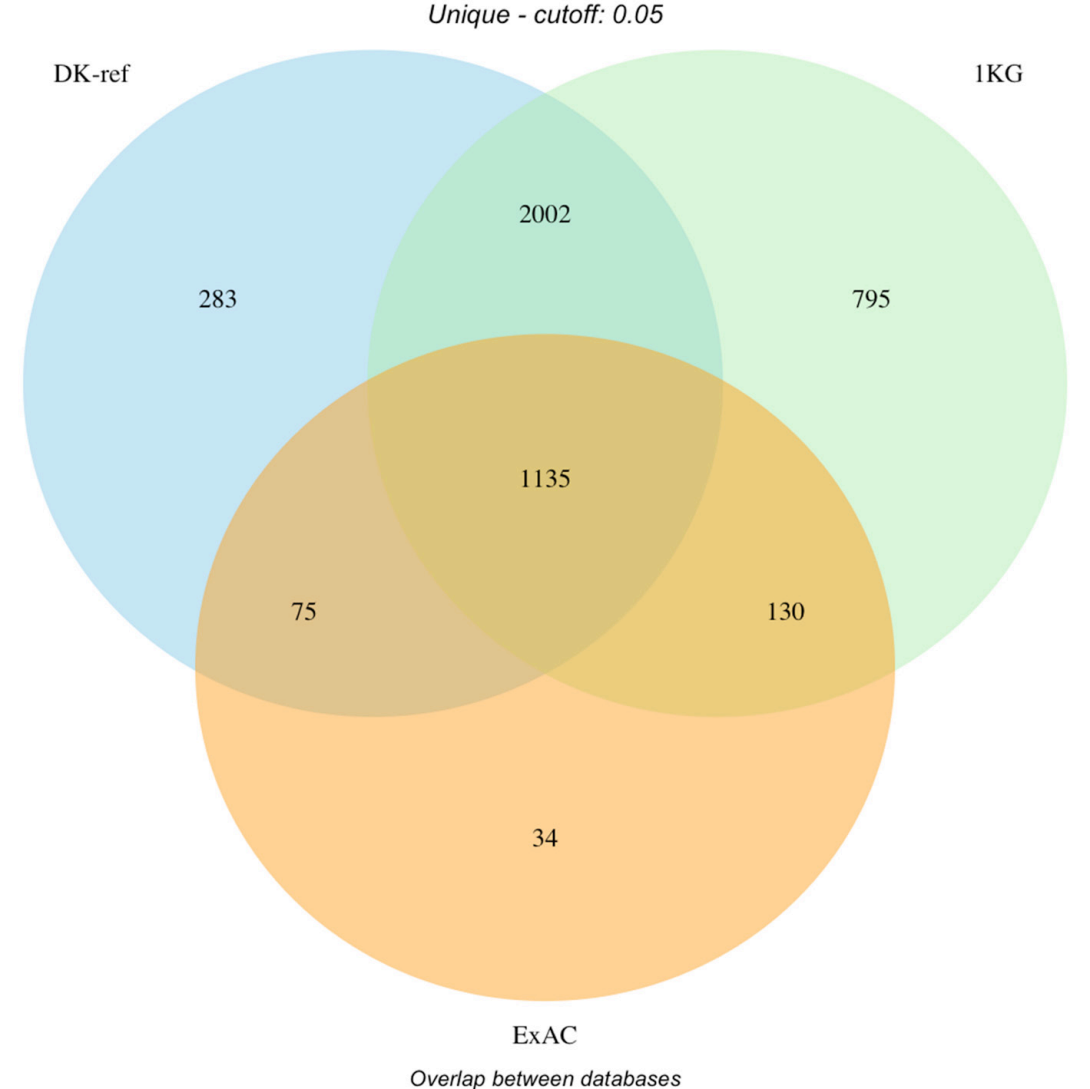
Filtering of variants by database (Danish reference genome, dbSNP and ExAC) Overlap of filtered variants between databases. DK-ref: Variants found i*n* >= 5% of parents from panel behind Danish reference genome. 1KG: Variants found in >= 5% of a population in 1000 Genomes Project. ExAC: Variants found in >= 5% of ExAC cohort globally or European subset.

**Figure 3:**
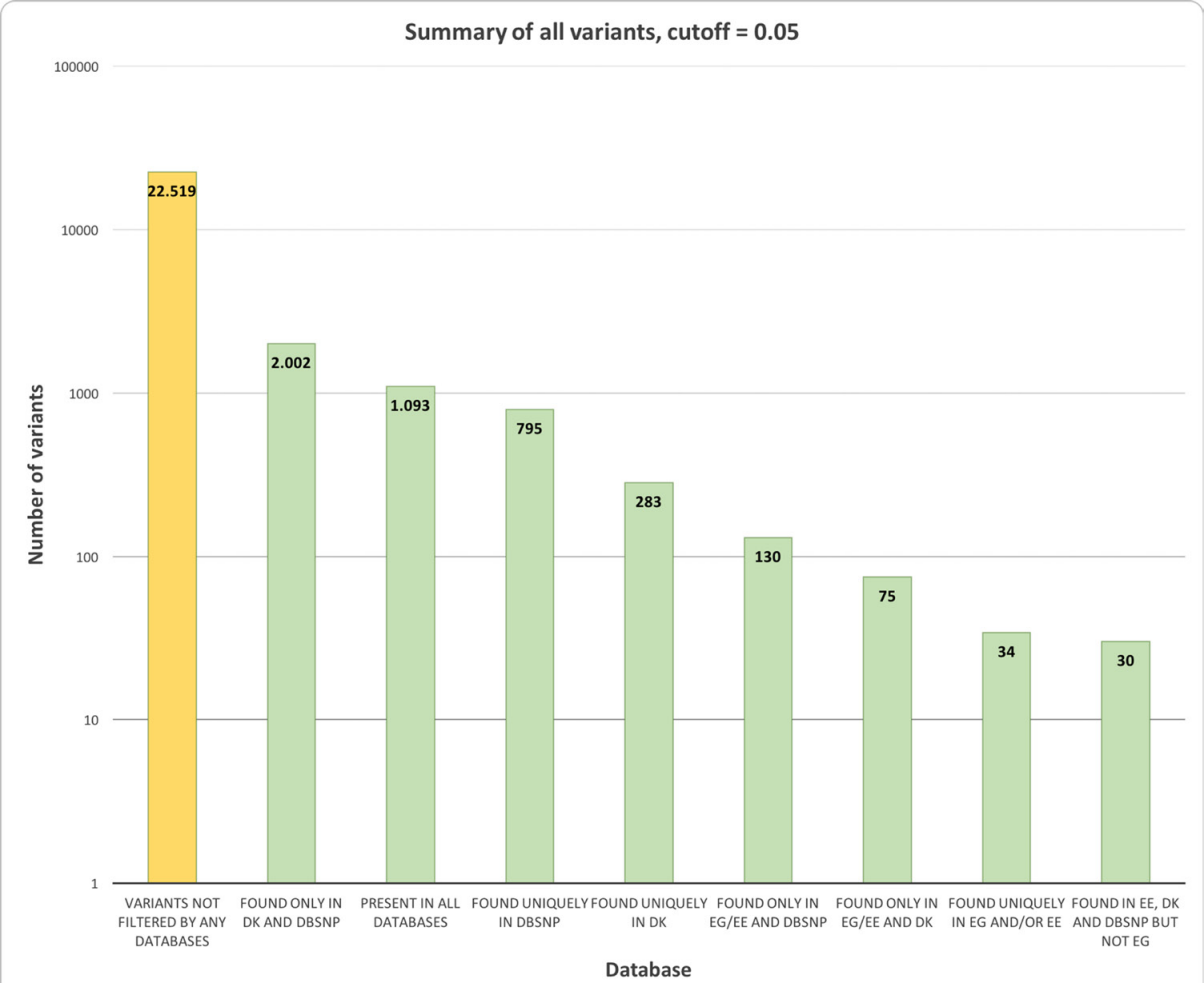
Survival plots of patients with mutations in the *BRAF* and *MPO* genes (**A**) Survival-plot of patients with mutation (red, *n* = 14) or reference (black, *n* = 81) in BRAF 600. Hazard ratio = 3 (1.6–5.7). (**B**) Survival-plot of patients with mutation (red, *n* = 5) or reference (black, *n* = 90) in *MPO* 332. Hazard ratio = 3.1 (1.2–7.9).

The overlap between the different databases is represented in Figure 2, whereas the distribution of variants in each category is represented in Figure 3. Supplementary Table 2 summarises the distribution of flags across the full set of variants. Note that not all combinations are possible (e.g. “G5” implies “CO”), and that the same variant will count multiple times if observed in multiple samples.

### Cox regression

To identify regions with predictive power regarding survival, a proportional hazards model (Cox regression) was applied to all regions identified with *OncodriveClust*. OncodriveClust identified 378 mutational hot-spots that will be referred hereafter as clusters. Given that the number of covariates (i.e. 378 clusters) is high, compared to the number of patients (*n* = 217), we used a LASSO-model, which will select one of a group of correlated predictors and shrink the rest to zero [32].

Cox regression survival analyses were carried out for the following groups:

1) OS for pooled group of cancer patients in stage I-IV

2) OS for pooled group of cancer patients in stage I-III

3) PFS for pooled group of cancer patients in stage I-IV

4) PFS for pooled group of cancer patients in stage I-III

5) PFS for CC patients in stage I-IV

6) PFS for CC patients in stage I-III

7) PFS for RC patients in stage I-IV

8) PFS for RC patients in stage I-III

As only 9 patients were in stage IV at time of operation, the differences in PFS with and without this group were minimal. Regions mutated in more than five samples were used as covariates for model selection.

Cox-regression analysis was performed with the *glmnet* R-package, which is an elastic net LASSO fit. The model uses an elastic net to bridge the gap between ridge regression (shrinking correlated predictors towards each other) and LASSO (discard non-influential coefficients). In the model, the parameter a is used to control the elastic net between a complete ridge model (α = 0) and a complete LASSO model (α = 1). Using varying degrees of a to favor either a ridge model or a LASSO model provided the same results, with only size of coefficients varying between the models.

Calculating standard error for non-zero coefficients is not meaningful for biased estimation methods such as LASSO, since this procedure aims at reducing the variance of estimators. Thus, to evaluate the significance of predictors carrying non-zero coefficients, these parameters are tested in a regular survival analysis to calculate statistical significance and hazard ratios.

## SUPPLEMENTARY MATERIALS FIGURES AND TABLES














