# Supplementary material for: Analysis of a gene panel for targeted sequencing of colorectal cancer samples
Source: Oncotarget. 2018 Jan 10;9(10):9043–60. doi: 10.18632/oncotarget.24138 (PMC5823670; doi:10.18632/oncotarget.24138)
Supplement: Supplementary file 1 [file oncotarget-09-9043-s001.pdf]

Analysis of a gene panel for targeted sequencing of colorectal cancer samples

SUPPLEMENTARY MATERIALS

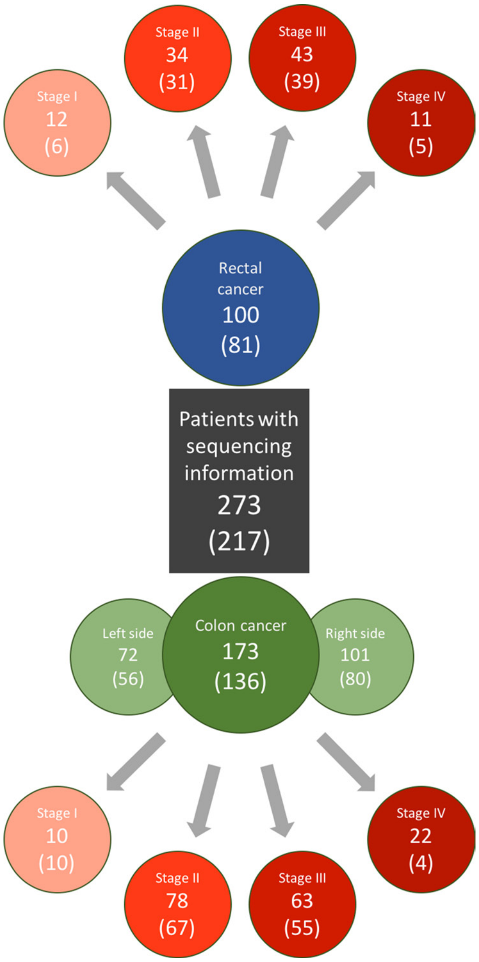

Supplementary Figure 1: Size of patient groups. Numbers in parentheses indicate number of patients with information on progression-free survival time.

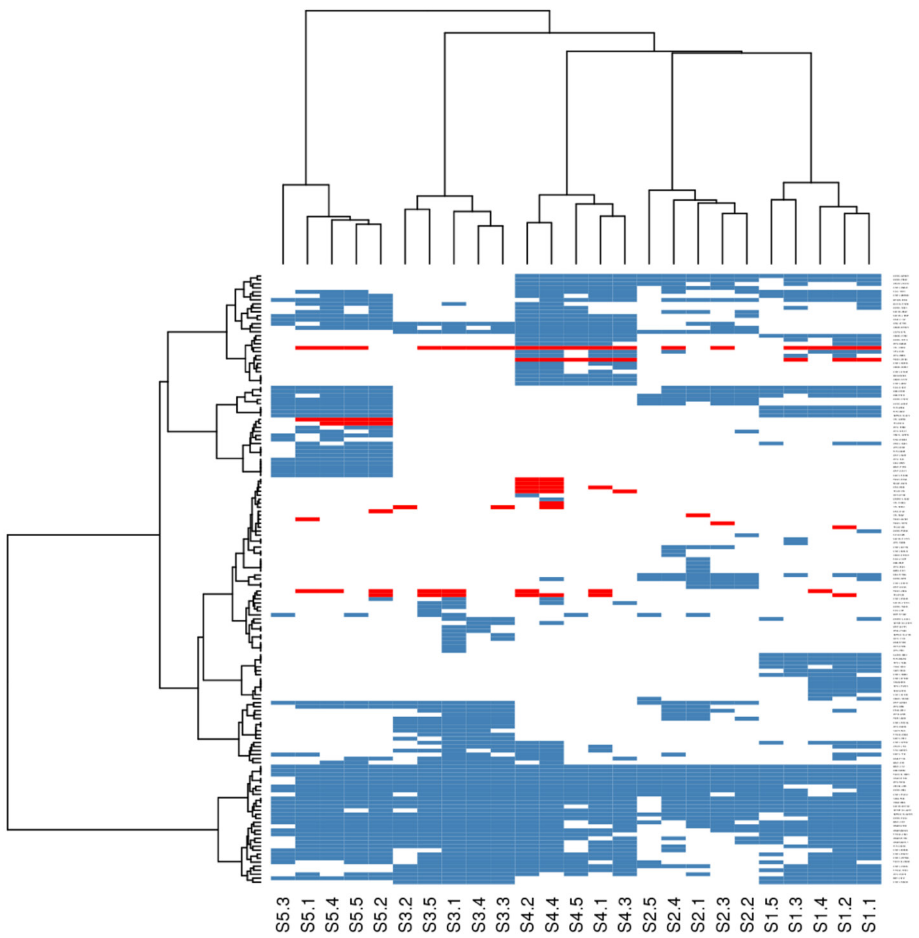

ID14−051−S1/variants.filtered.qual100+.dp30+

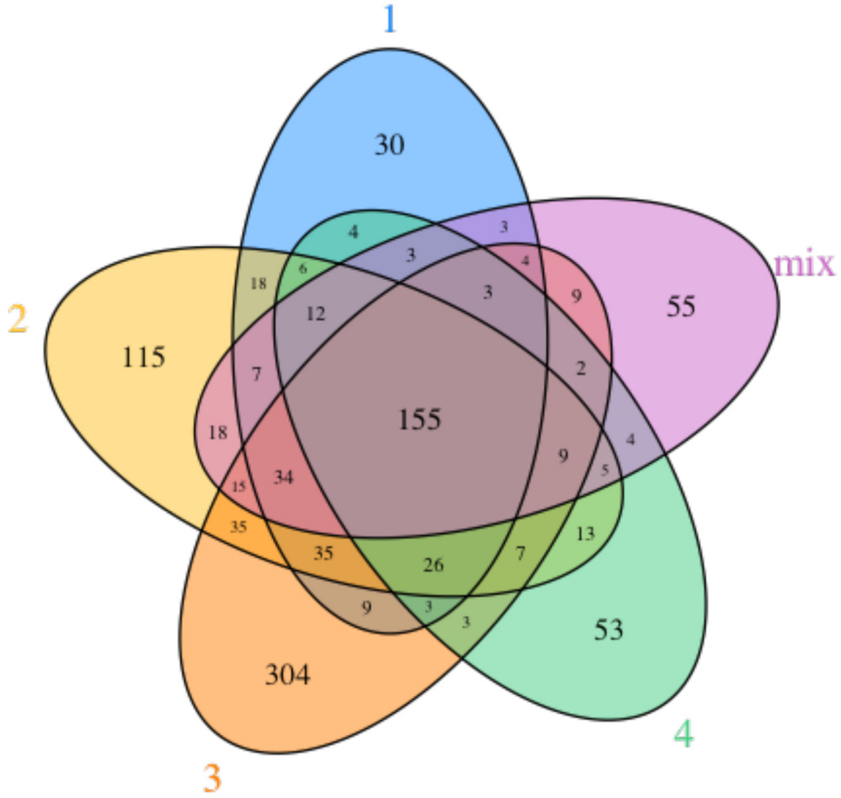

/1000 of total mutations (17052 mutations)

ID14−051−S2/variants.filtered.qual100+.dp30+

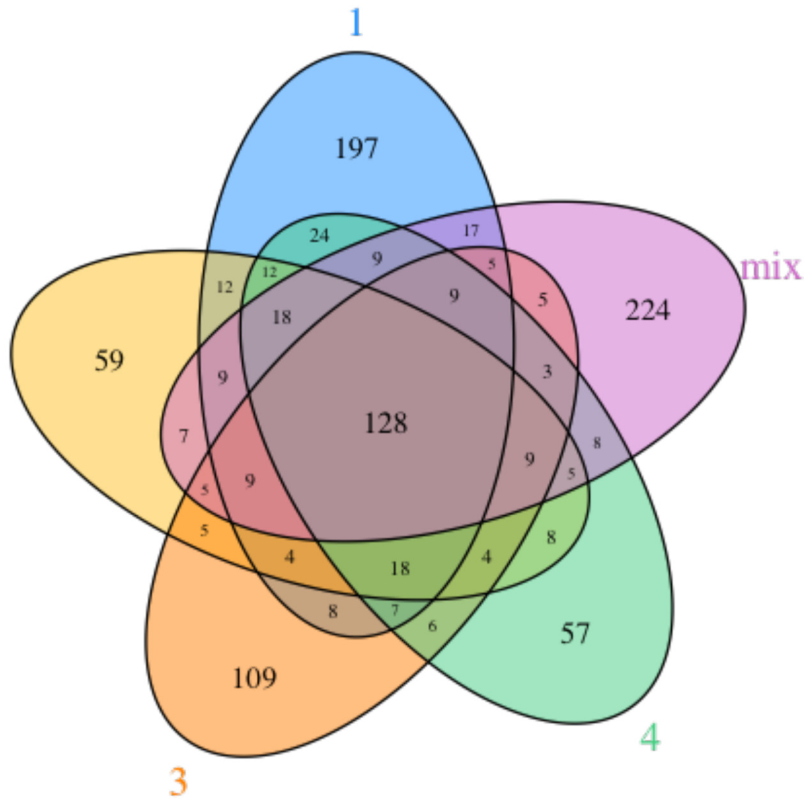

/1000 of total mutations (20171 mutations)

ID14−051−S3/variants.filtered.qual100+.dp30+

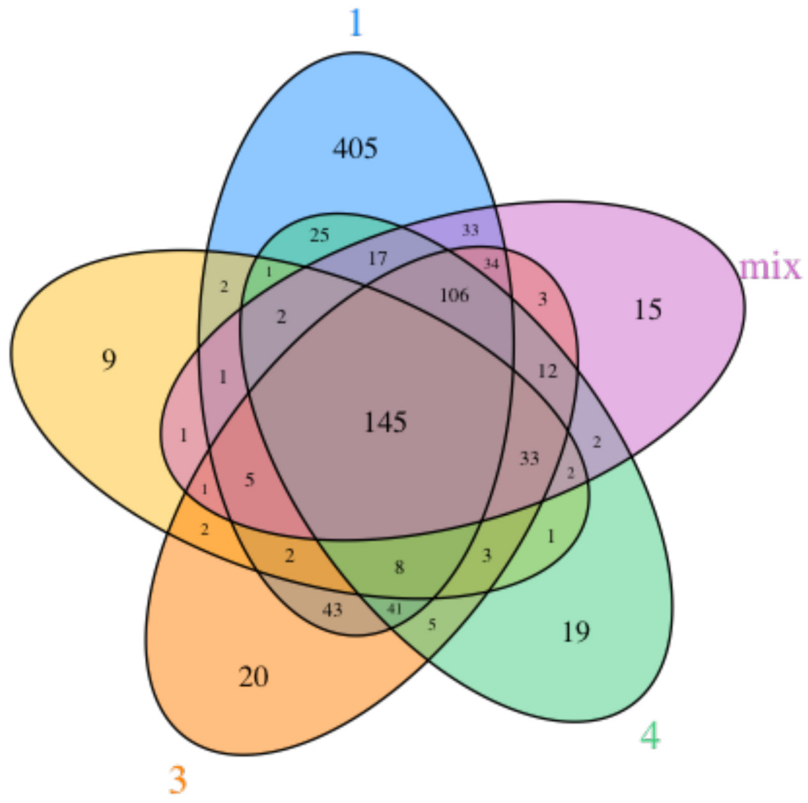

/1000 of total mutations (14314 mutations)

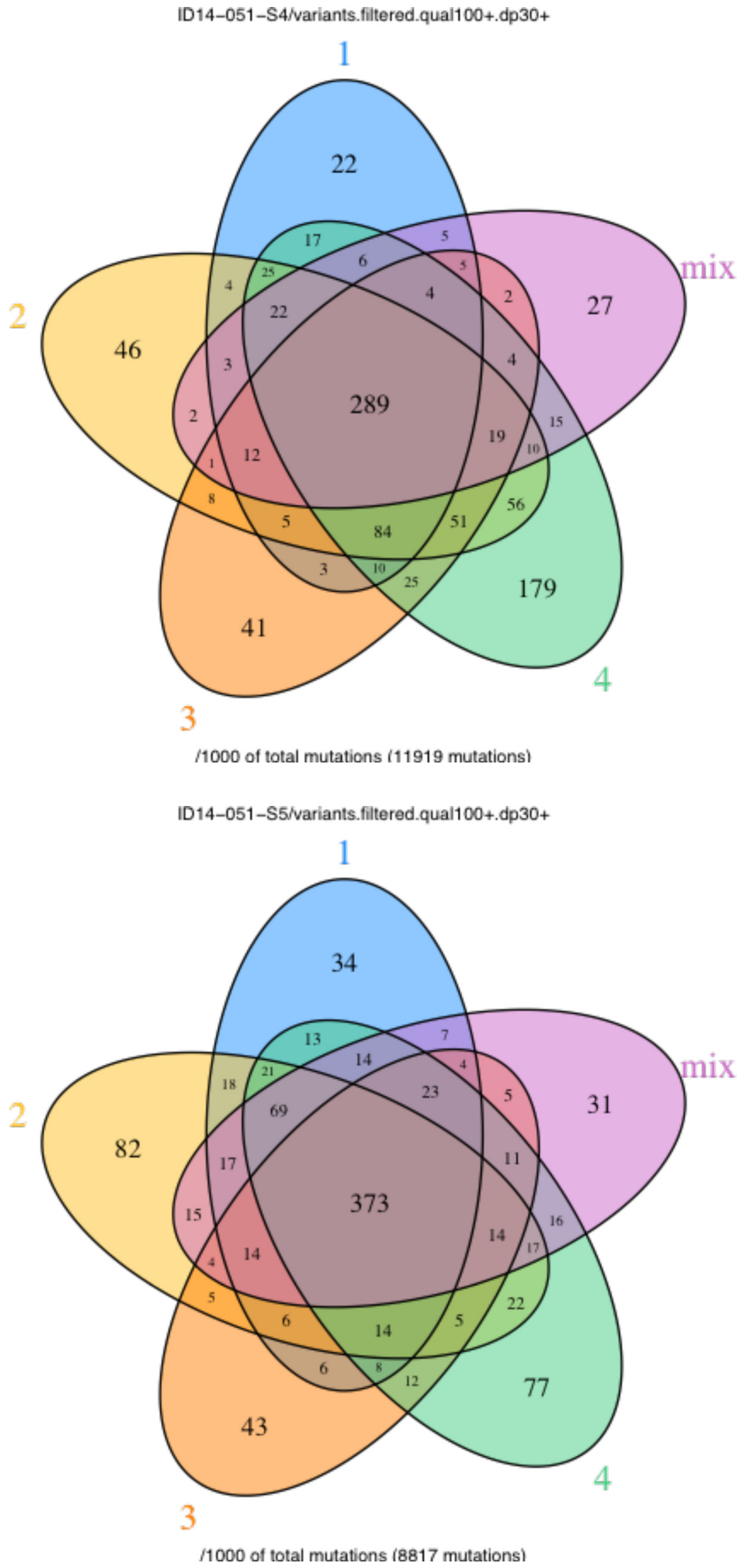

Supplementary Figure 2: Sample heterogeneity analysis.

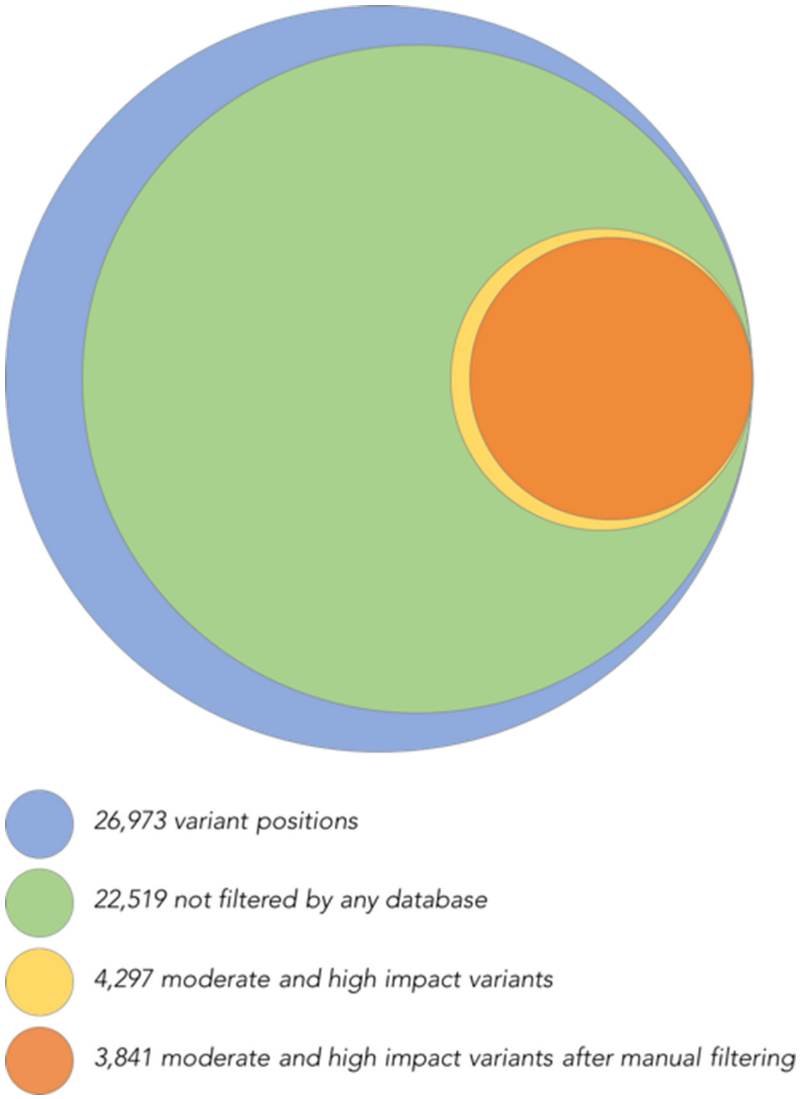

Supplementary Figure 3: Number of high quality variants detected before and after database and manual filtering.

Supplementary Table 1: Composition of the 5 cases used in the pilot study to evaluate the clinical applicability of the laboratory technique, regarding DNA extraction as a function of tumour infiltrating inflammatory cells and intra-tumoural heterogeneity. To evaluate intra-tumoural heterogeneity, sections cut from individual tumour tissue block were placed separately, or mutually from all 4 tumour tissue blocks, in one microtube, respectively. See Supplementary_Table_1

Supplementary Table 2: Distribution of the exclusion flags used in the filtering of variants. See Supplementary_Table_2

Supplementary Table 3: Description of the sources used for the inclusion of genes in the extended gene panel. See Supplementary_Table_3

Supplementary Table 4: Content of the gene panel. A total of 1426 unique genes were scored based on information about their mutation frequency and known association with CRC. See Supplementary_Table_4

Supplementary File 1: Description of the 56,008 probes designed with Agilent's SureSelect E-array software (3x tiling density) and moderately stringent masking. The probes covered exons (±10 nt.), 3'UTRs and 5'UTRs of the 266 candidate genes in our panel. See Supplementary_File_1

Supplementary File 2: Histograms of the distribution of the alternative allele frequency for all rs-ids present in 4 or more samples. See Supplementary_File_2
