# Supplementary material for: Analysis of a gene panel for targeted sequencing of colorectal cancer samples
Source: Oncotarget. 2018 Jan 10;9(10):9043–60. doi: 10.18632/oncotarget.24138 (PMC5823670; doi:10.18632/oncotarget.24138)
Supplement: Supplementary file 2 [file oncotarget-09-9043-s002.pdf]

SureDesign

SureSelect DNA Design Report

General Information

User: s.raynere@wh.iov.cn
Workgroup: bds
Folder: bds

File Type: SureSelect DNA Design Report
Created By: SureSelect DNA Standard Design Wizard
Date: 03-Feb-2015

Design Information

Design Name: test
Species: H. sapiens (H. sapiens, hg19, GRCh37, February 2009)

Target Summary

266 Target IDs resolved to 266 targets comprising 3551 regions.
0 Target IDs were not found.
Region Size: 1.357 Mbp

Probe Summary

Total Probes: 56008
Total Probes Size: 1.535 Mbp
Coverage: 97.81054%
Recommended Minimum Sequencing per Sample: 306.951 Mbp
Pricing: Tier 2 (Probe Region Size = 0.5 - 2.999 Mbp; up to 57.5K probes)

Target Parameters

Databases: RefSeq, Ensembl, CCDS, Gencode, VEGA, SNP, CytoBand
Region: Coding Exons + UTRs + 5' UTR + 3' UTR
Region Extension: 10 bases from 3' end and 10 bases from 5' end.
Allow Synonyms: No

Probe Tiling Parameters

Tiling density: 3x
Masking: Moderately Stringent
Boosting: MaximizePerformance

Design Details

Target ID	Regions	Coverage	High Coverage (>= 90%)	Low Coverage (< 90%)
ABCB1	35	98.96405 %	33	2
ABCC1	32	99.170586 %	31	1
ABCC2	32	100.0 %	32	0
ABCC3	35	98.50916 %	33	2
ABCC4	35	93.490654 %	32	3
ABCC5	35	96.80422 %	33	2
ABCG2	18	100.0 %	18	0
ACOT4	3	100.0 %	3	0
ACVR1B	11	99.75778 %	11	0
ACVR2A	12	100.0 %	12	0
ADAMTSL4-AS1	4	91.935486 %	2	2
AFF2	22	99.70926 %	22	0
AKT1	16	93.919655 %	15	1
ALB	19	100.0 %	19	0
ALPI	10	100.0 %	10	0
AMER1	2	100.0 %	2	0
ANO4	31	100.0 %	31	0
APC	25	99.51002 %	25	0
ARID1A	19	100.0 %	19	0
ARID2	24	95.494705 %	22	2
ATM	65	95.413315 %	64	1
ATP5B	10	100.0 %	10	0
ATP5E	2	100.0 %	2	0
ATP6V0D2	13	78.10398 %	10	3
ATP7A	22	97.225464 %	21	1
ATP7B	21	100.0 %	21	0
AXIN1	9	95.7236 %	8	1
AXIN2	11	100.0 %	11	0
B2M	3	100.0 %	3	0
BAX	3	95.5739 %	2	1
BBC3	5	97.08428 %	4	1
BCHE	7	96.927376 %	6	1

Target ID	Regions	Coverage	High Coverage (>= 90%)	Low Coverage (< 90%)
BCLAF1	14	100.0 %	14	0
BCOR	19	100.0 %	19	0
BRAF	21	100.0 %	21	0
C1ORF170	5	100.0 %	5	0
C1QA	4	100.0 %	4	0
C1QB	5	100.0 %	5	0
C1QC	3	100.0 %	3	0
C1R	13	99.36306 %	13	0
C1S	16	92.82247 %	13	3
CASP8	17	71.97544 %	15	2
CCDC160	3	100.0 %	3	0
CCR2	4	100.0 %	4	0
CD70	4	95.623146 %	3	1
CDA	4	100.0 %	4	0
CDC27	20	100.0 %	20	0
CDH2	20	97.75302 %	19	1
CDK12	14	100.0 %	14	0
CES1	14	100.0 %	14	0
CES2	12	92.26618 %	11	1
CHRDL1	12	100.0 %	12	0
CNBD1	19	94.76174 %	18	1
COX6B2	5	93.98752 %	5	0
CPXCR1	3	100.0 %	3	0
CRTC1	15	100.0 %	15	0
CSMD1	82	100.0 %	82	0
CSMD3	73	99.87991 %	73	0
CTNNB1	22	100.0 %	22	0
CXCR2	6	84.57177 %	5	1
CYP1A1	7	100.0 %	7	0
CYP1A2	7	60.23875 %	6	1
CYP1B1	6	100.0 %	6	0
CYP24A1	12	100.0 %	12	0
CYP27B1	9	100.0 %	9	0
CYP2A6	9	100.0 %	9	0

Target ID	Regions	Coverage	High Coverage (>= 90%)	Low Coverage (< 90%)
CYP2B6	9	82.20262 %	8	1
CYP2C8	10	97.535934 %	9	1
CYP2C9	10	99.76872 %	10	0
CYP2E1	11	100.0 %	11	0
CYP3A4	14	98.107796 %	14	0
CYP3A5	14	80.17106 %	13	1
CYP3A7	15	97.97313 %	14	1
DKK2	6	100.0 %	6	0
DKK4	4	100.0 %	4	0
DMD	93	98.84416 %	91	2
DNAH5	81	99.58559 %	81	0
DPYD	26	100.0 %	26	0
EDNRB	10	100.0 %	10	0
EGFR	36	99.40107 %	35	1
EIF4A2	2	100.0 %	2	0
ELF3	6	94.93913 %	6	0
EP300	31	100.0 %	31	0
EPCAM	10	97.646194 %	9	1
ERBB2	33	98.63278 %	32	1
ERBB3	31	98.300446 %	30	1
EVC2	26	100.0 %	26	0
FAM194B	17	100.0 %	17	0
FAM5C	11	100.0 %	11	0
FAT4	18	100.0 %	18	0
FBXW7	17	98.72797 %	16	1
FCGR1A	7	100.0 %	7	0
FCGR2A	10	96.485855 %	9	1
FCGR2B	8	99.056335 %	8	0
FCGR2C	8	94.155655 %	7	1
FCGR3A	9	100.0 %	9	0
FCGR3B	6	100.0 %	6	0
FZD3	8	94.751564 %	8	0
FZD9	1	100.0 %	1	0
GGT1	18	95.16356 %	16	2

Target ID	Regions	Coverage	High Coverage (>= 90%)	Low Coverage (< 90%)
GOT1	8	100.0 %	8	0
GPC6	9	100.0 %	9	0
GRIK3	16	100.0 %	16	0
GSTM1	7	100.0 %	7	0
GSTP1	3	100.0 %	3	0
GSTT1	8	89.60322 %	7	1
HBE1	5	98.24047 %	4	1
HBG2	9	89.35206 %	7	2
HIST1H2AG	1	95.690414 %	1	0
HIST1H2AI	1	100.0 %	1	0
HIST1H2AK	1	100.0 %	1	0
HIST1H2AL	1	100.0 %	1	0
HIST1H2AM	1	100.0 %	1	0
HIST1H3A	1	100.0 %	1	0
HIST1H3B	1	100.0 %	1	0
HIST1H3C	1	100.0 %	1	0
HIST1H3D	2	100.0 %	2	0
HIST1H3E	1	100.0 %	1	0
HIST1H3F	1	100.0 %	1	0
HIST1H3G	1	100.0 %	1	0
HIST1H3H	1	100.0 %	1	0
HIST1H3I	1	100.0 %	1	0
HIST1H3J	2	100.0 %	2	0
HIST1H4A	2	100.0 %	2	0
HIST1H4B	1	100.0 %	1	0
HIST1H4C	1	100.0 %	1	0
HIST1H4D	1	100.0 %	1	0
HIST1H4E	1	100.0 %	1	0
HIST1H4F	1	100.0 %	1	0
HIST1H4H	2	100.0 %	2	0
HIST1H4I	1	97.39382 %	1	0
HIST1H4J	1	100.0 %	1	0
HIST1H4K	1	100.0 %	1	0
HIST1H4L	1	100.0 %	1	0

Target ID	Regions	Coverage	High Coverage (>= 90%)	Low Coverage (< 90%)
HIST2H4A	4	100.0 %	4	0
HIST2H4B	4	100.0 %	4	0
HIST4H4	3	87.35315 %	2	1
HUS1	12	95.539566 %	11	1
IDH2	12	97.34899 %	12	0
IL13RA1	13	98.3531 %	13	0
IRF5	14	97.937706 %	13	1
KIF2B	1	100.0 %	1	0
KRAS	6	98.52013 %	6	0
KRBOX1	9	97.934494 %	8	1
KRTAP22-2	1	100.0 %	1	0
KRTAP4-3	1	100.0 %	1	0
KRTAP4-5	1	100.0 %	1	0
KRTAP5-5	1	100.0 %	1	0
LIFR	27	97.70254 %	25	2
LRP1B	94	99.77358 %	93	1
LRP2	80	100.0 %	80	0
LSM2	8	91.58223 %	6	2
LURAP1L	2	100.0 %	2	0
MAP2K1	11	100.0 %	11	0
MAP2K4	13	100.0 %	13	0
MAP7	22	100.0 %	22	0
MAPK8IP1	13	98.15998 %	12	1
MDM2	15	88.03032 %	13	2
MIER3	18	100.0 %	18	0
MLH1	24	96.59896 %	23	1
MLH3	14	100.0 %	14	0
MMP26	8	100.0 %	8	0
MPO	11	100.0 %	11	0
MRVI1	24	98.26822 %	23	1
MSH2	20	97.87958 %	18	2
MSH6	14	90.80398 %	13	1
MT1A	3	100.0 %	3	0
MT2A	3	100.0 %	3	0

Target ID	Regions	Coverage	High Coverage (>= 90%)	Low Coverage (< 90%)
MTHFR	13	95.57953 %	13	0
MYO1B	37	98.294266 %	36	1
NPAP1	1	90.338165 %	1	0
NQO1	6	97.05373 %	6	0
NR1H4	13	93.436295 %	12	1
NRAS	7	100.0 %	7	0
NRXN1	39	99.272316 %	39	0
NTN4	11	100.0 %	11	0
OR10A7	1	100.0 %	1	0
OR2L13	3	100.0 %	3	0
OR2M4	1	100.0 %	1	0
OR2W3	3	96.805115 %	3	0
OTOL1	4	100.0 %	4	0
PCBP1	1	100.0 %	1	0
PCDHA1	4	100.0 %	4	0
PCDHA10	4	100.0 %	4	0
PCDHA2	5	100.0 %	5	0
PCDHA3	4	100.0 %	4	0
PCDHA4	4	100.0 %	4	0
PCDHA5	4	100.0 %	4	0
PCDHA6	4	100.0 %	4	0
PCDHA7	4	100.0 %	4	0
PCDHA8	4	100.0 %	4	0
PCDHGA1	4	100.0 %	4	0
PCDHGA2	4	100.0 %	4	0
PCDHGA3	4	100.0 %	4	0
PCDHGA4	4	100.0 %	4	0
PCDHGA5	4	100.0 %	4	0
PCDHGA6	4	100.0 %	4	0
PCDHGA7	4	100.0 %	4	0
PCDHGA8	4	100.0 %	4	0
PCDHGB1	4	100.0 %	4	0
PCDHGB2	4	100.0 %	4	0
PCDHGB3	4	100.0 %	4	0

Target ID	Regions	Coverage	High Coverage (>= 90%)	Low Coverage (< 90%)
PCDHGB4	4	100.0 %	4	0
PIK3CA	23	100.0 %	23	0
PIK3R1	20	100.0 %	20	0
PMS1	17	98.51571 %	17	0
PMS2	14	95.94402 %	13	1
PPAT	12	92.789474 %	11	1
PRIM2	17	99.478714 %	17	0
PTEN	10	99.492584 %	9	1
PTPLA	7	87.5 %	6	1
PTPN12	23	97.368416 %	22	1
RBM10	25	100.0 %	25	0
RPL41	3	100.0 %	3	0
RYR2	114	100.0 %	114	0
SCN10A	27	100.0 %	27	0
SCN7A	27	95.731285 %	25	2
SETD2	29	97.64584 %	24	5
SH3BGRL3	2	100.0 %	2	0
SIRT4	4	100.0 %	4	0
SLC22A1	11	100.0 %	11	0
SLC22A2	17	98.57165 %	17	0
SLC22A3	11	99.29672 %	11	0
SLC22A7	12	100.0 %	12	0
SLC29A1	15	100.0 %	15	0
SLC31A1	7	93.70315 %	6	1
SLC9A9	20	98.36645 %	18	2
SLCO1B1	15	100.0 %	15	0
SMAD2	12	99.267456 %	12	0
SMAD3	12	100.0 %	12	0
SMAD4	16	97.90283 %	15	1
SOD1	6	99.812996 %	6	0
SOX9	3	96.09512 %	3	0
SPHK2	9	100.0 %	9	0
SUPT4H1	5	100.0 %	5	0
SYNE1	150	98.07359 %	147	3

Target ID	Regions	Coverage	High Coverage (>= 90%)	Low Coverage (< 90%)
TBP	9	100.0 %	9	0
TCERG1	27	100.0 %	27	0
TCF7L2	19	100.0 %	19	0
TGFBR2	10	100.0 %	10	0
TIMM8B	3	100.0 %	3	0
TMEM257	1	100.0 %	1	0
TMPRSS11A	10	100.0 %	10	0
TNFRSF10C	10	97.6438 %	9	1
TOP1	21	100.0 %	21	0
TOP1MT	27	100.0 %	27	0
TP53	14	89.99052 %	12	2
TRAF3	12	100.0 %	12	0
TRIM23	15	100.0 %	15	0
TRPS1	15	100.0 %	15	0
TSHZ3	2	98.82557 %	1	1
TYMP	6	90.573654 %	5	1
TYMS	7	100.0 %	7	0
UBE2NL	1	100.0 %	1	0
UGT1A1	6	89.928055 %	5	1
UGT1A9	5	100.0 %	5	0
UMPS	9	96.532616 %	7	2
UPP1	12	96.598366 %	12	0
UPP2	10	96.614494 %	9	1
VEGFA	4	100.0 %	4	0
VMO1	3	100.0 %	3	0
VTI1A	13	94.99366 %	11	2
WBSCR17	14	87.19441 %	13	1
ZC3H13	21	99.28852 %	20	1
ZNF148	14	98.73779 %	13	1
ZNF208	4	76.668846 %	3	1
