# Supplementary material for: Analysis of a gene panel for targeted sequencing of colorectal cancer samples
Source: Oncotarget. 2018 Jan 10;9(10):9043–60. doi: 10.18632/oncotarget.24138 (PMC5823670; doi:10.18632/oncotarget.24138)
Supplement: Supplementary file 3 [file oncotarget-09-9043-s003.pdf]

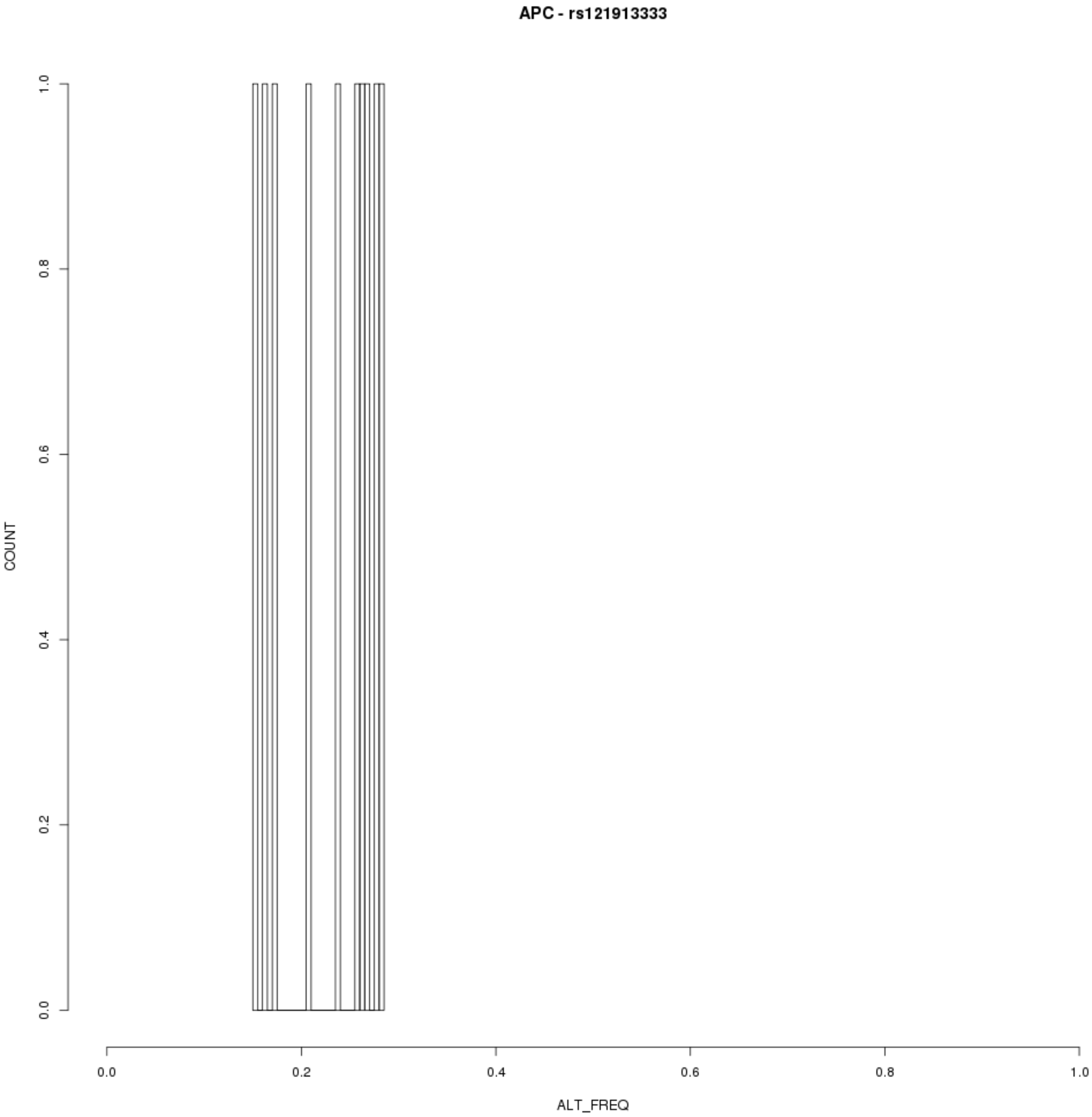
APC - rs121913333
COUNT
0.0
0.2
0.4
0.6
0.8
1.0
ALT_FREQ

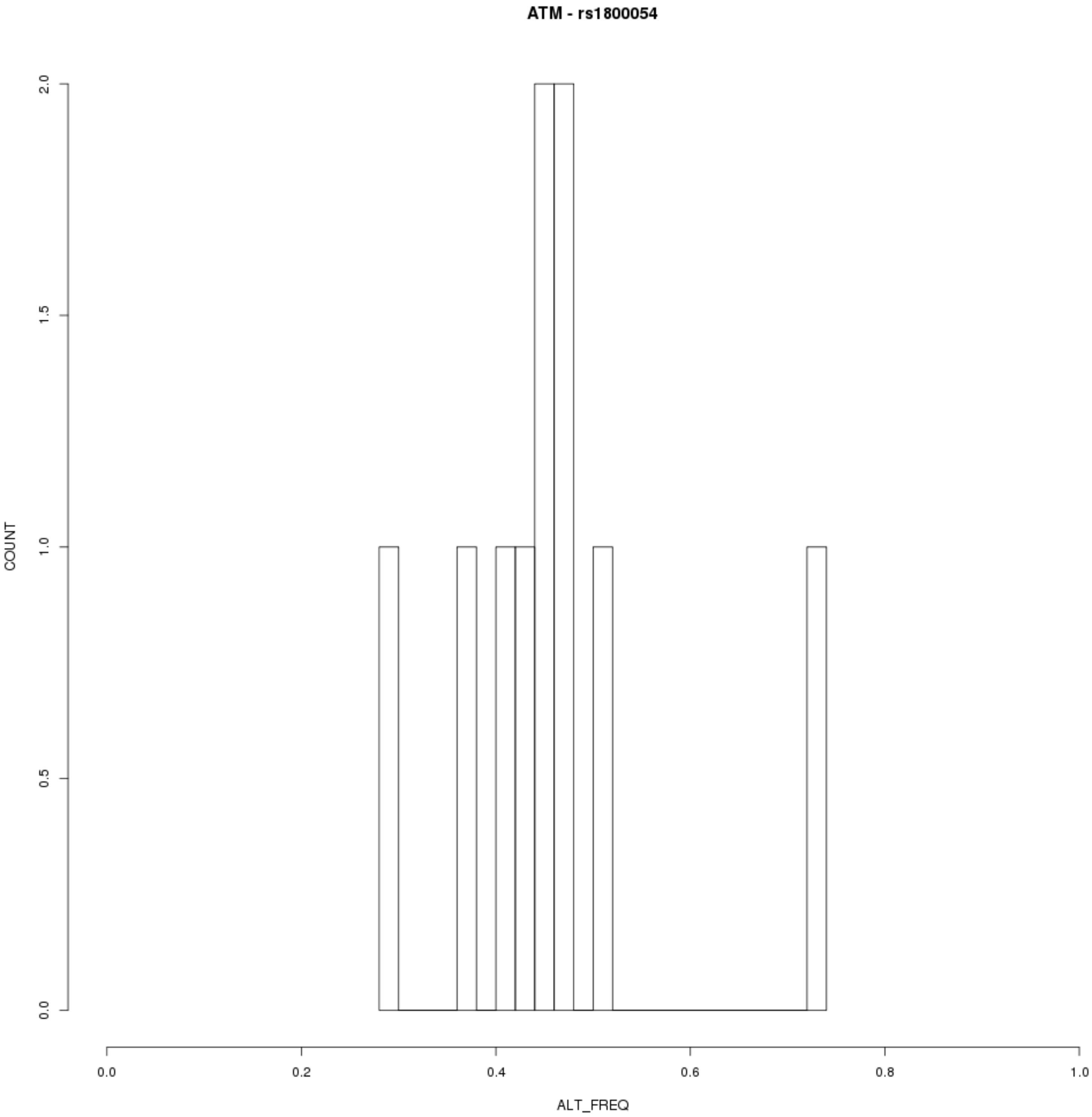

ATM - rs1800054
COUNT
ALT_FREQ
0.0
0.5
1.0
1.5
2.0
0.0
0.2
0.4
0.6
0.8
1.0

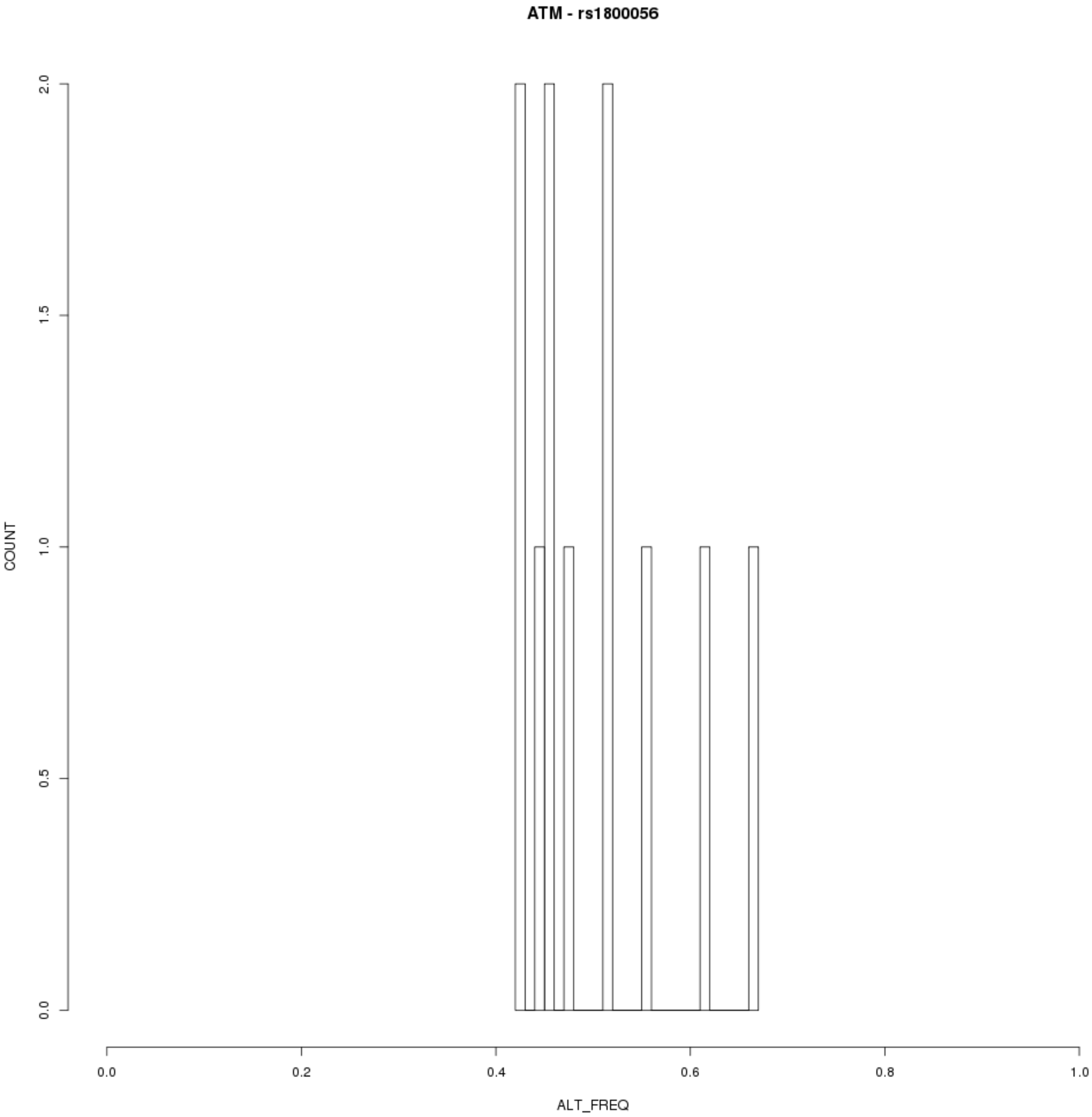
ATM - rs1800056
COUNT
ALT_FREQ
0.0
0.5
1.0
1.5
2.0
0.0
0.2
0.4
0.6
0.8
1.0

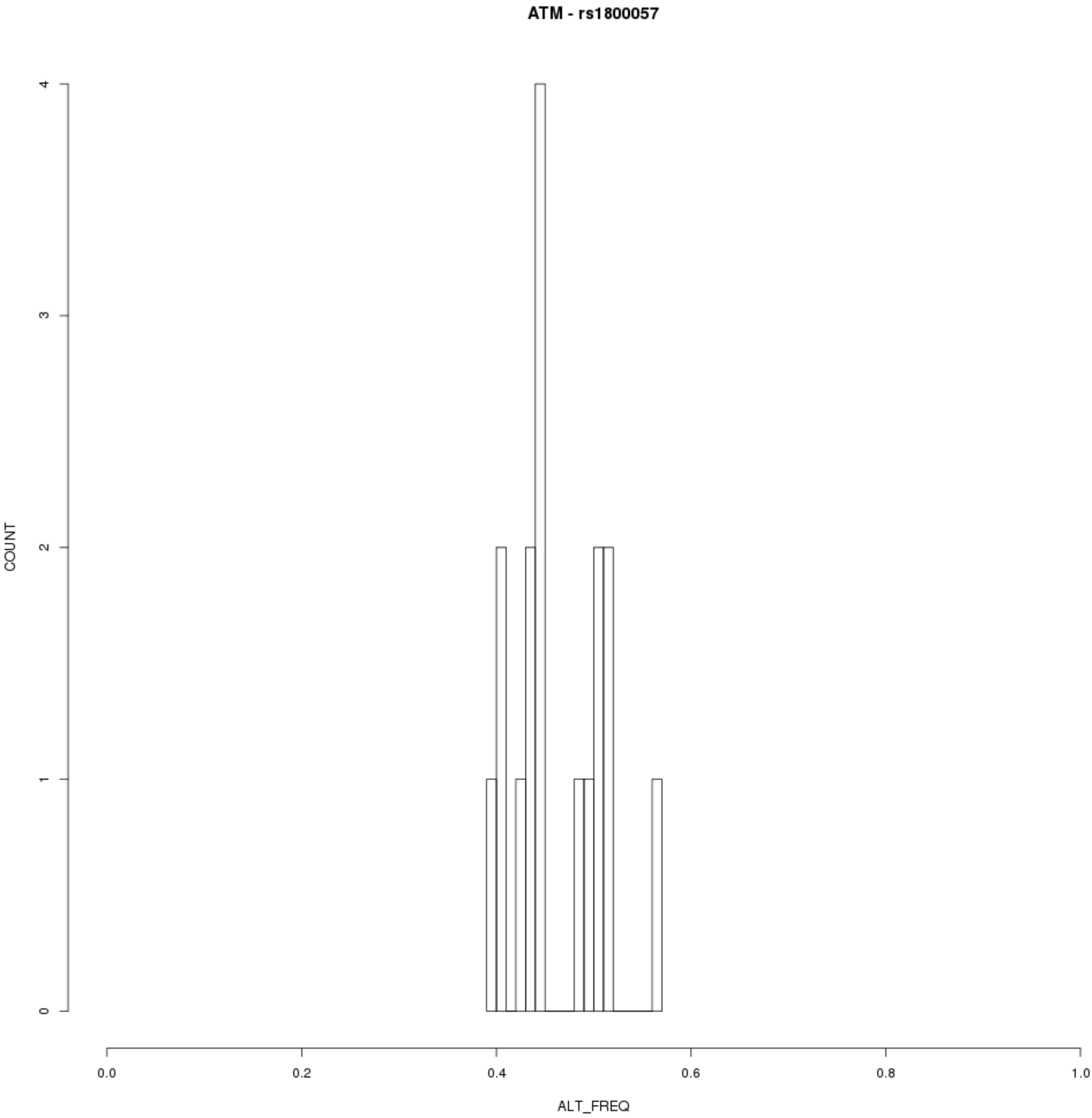
ATM - rs1800057
COUNT
0
1
2
3
4
0.0
0.2
0.4
0.6
0.8
1.0
ALT_FREQ

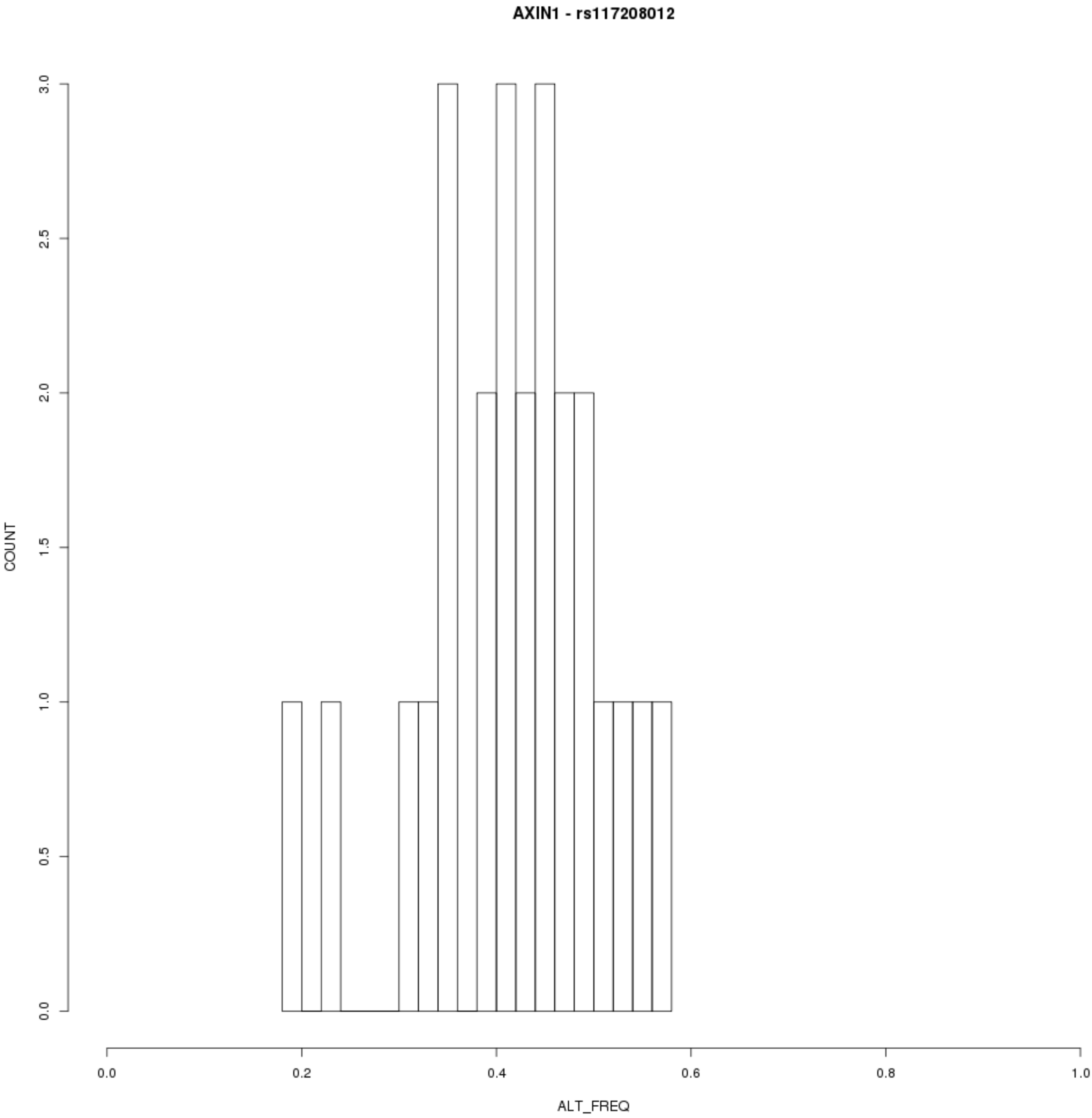

AXIN1 - rs117208012
COUNT
ALT_FREQ
0.0
0.5
1.0
1.5
2.0
2.5
3.0
0.0
0.2
0.4
0.6
0.8
1.0

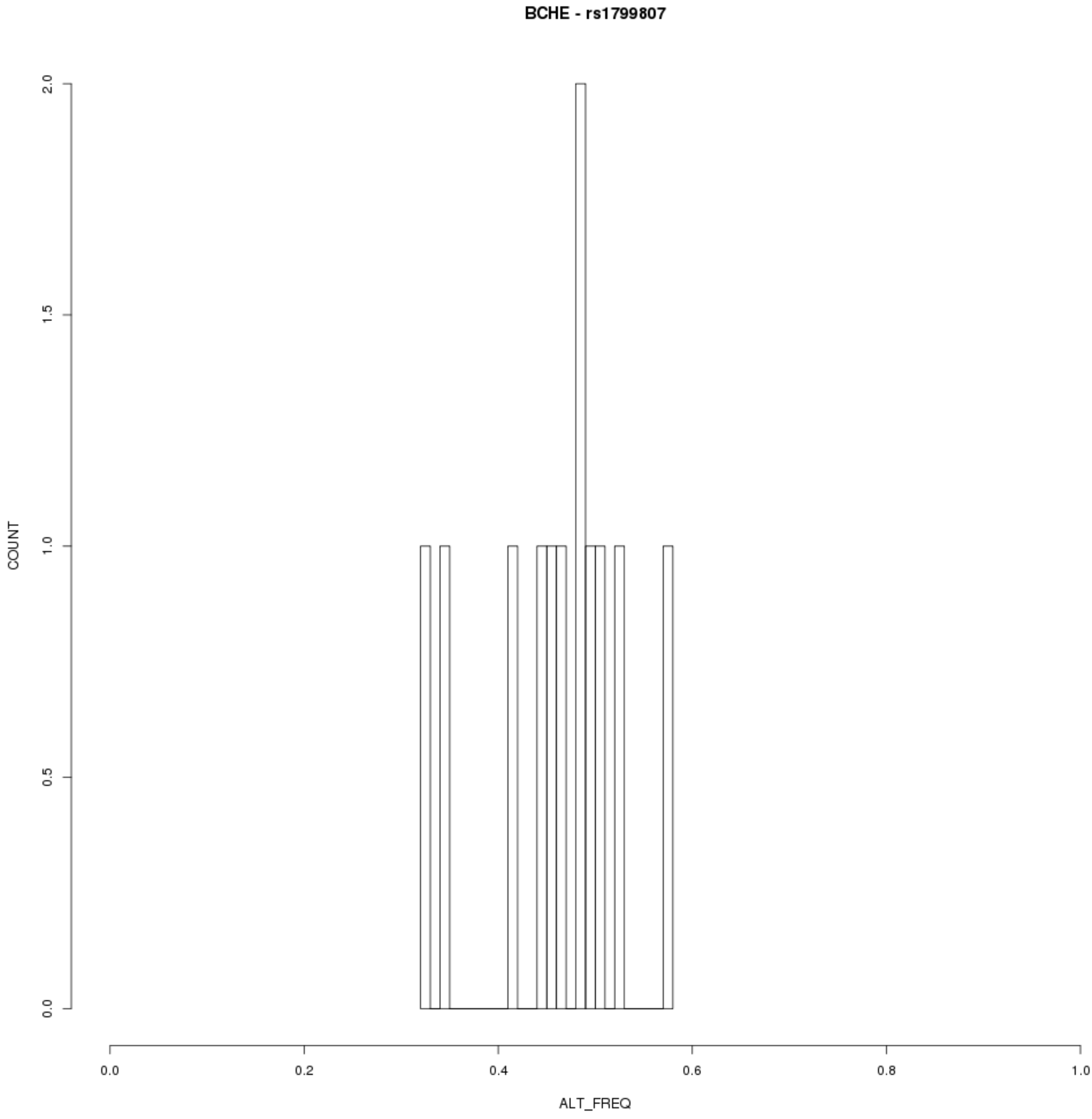

BCHE - rs1799807
COUNT
0.0
0.5
1.0
1.5
2.0
ALT_FREQ
0.0
0.2
0.4
0.6
0.8
1.0

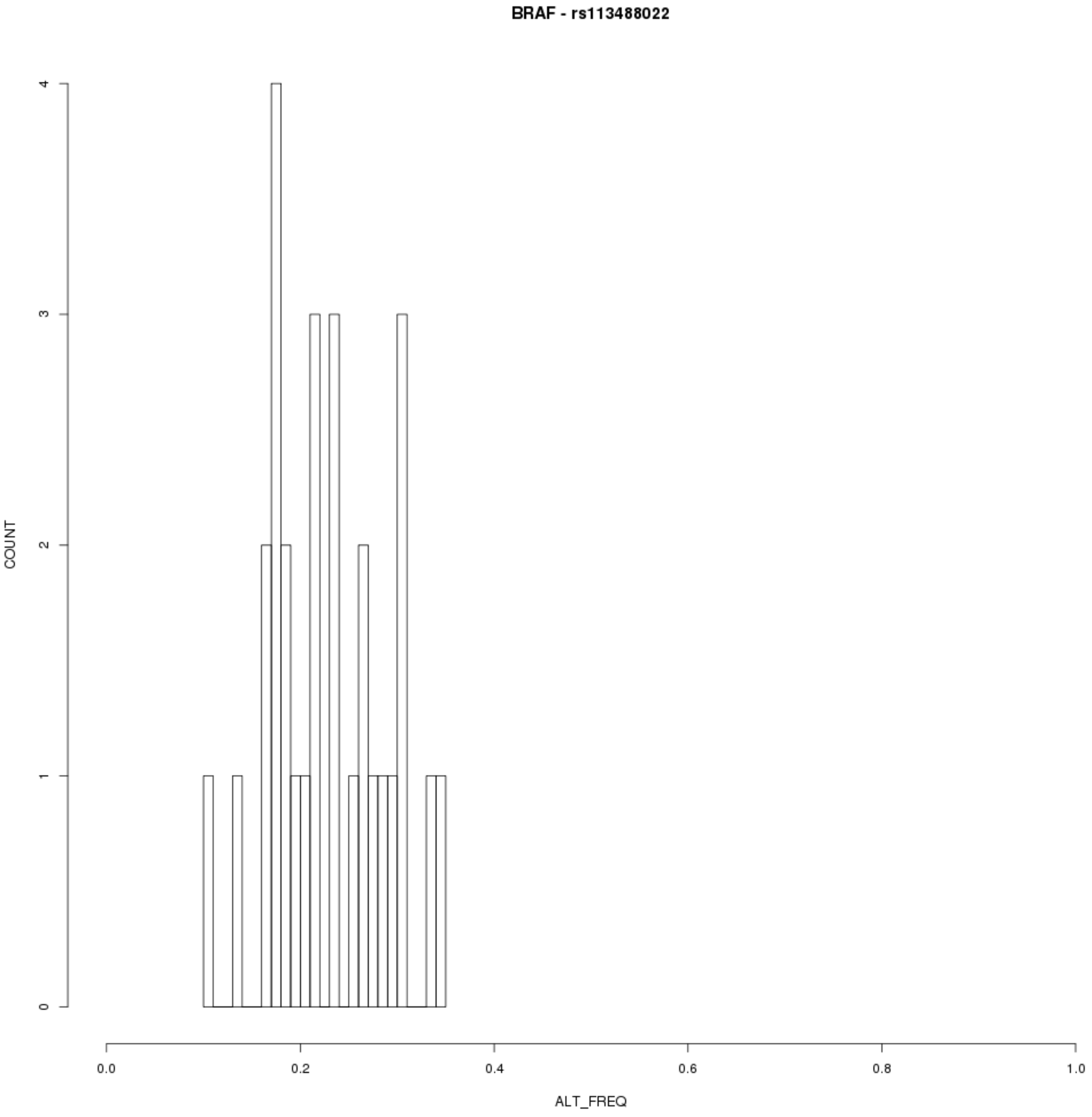
BRAF - rs113488022
COUNT
ALT_FREQ
0
1
2
3
4
0.0
0.2
0.4
0.6
0.8
1.0

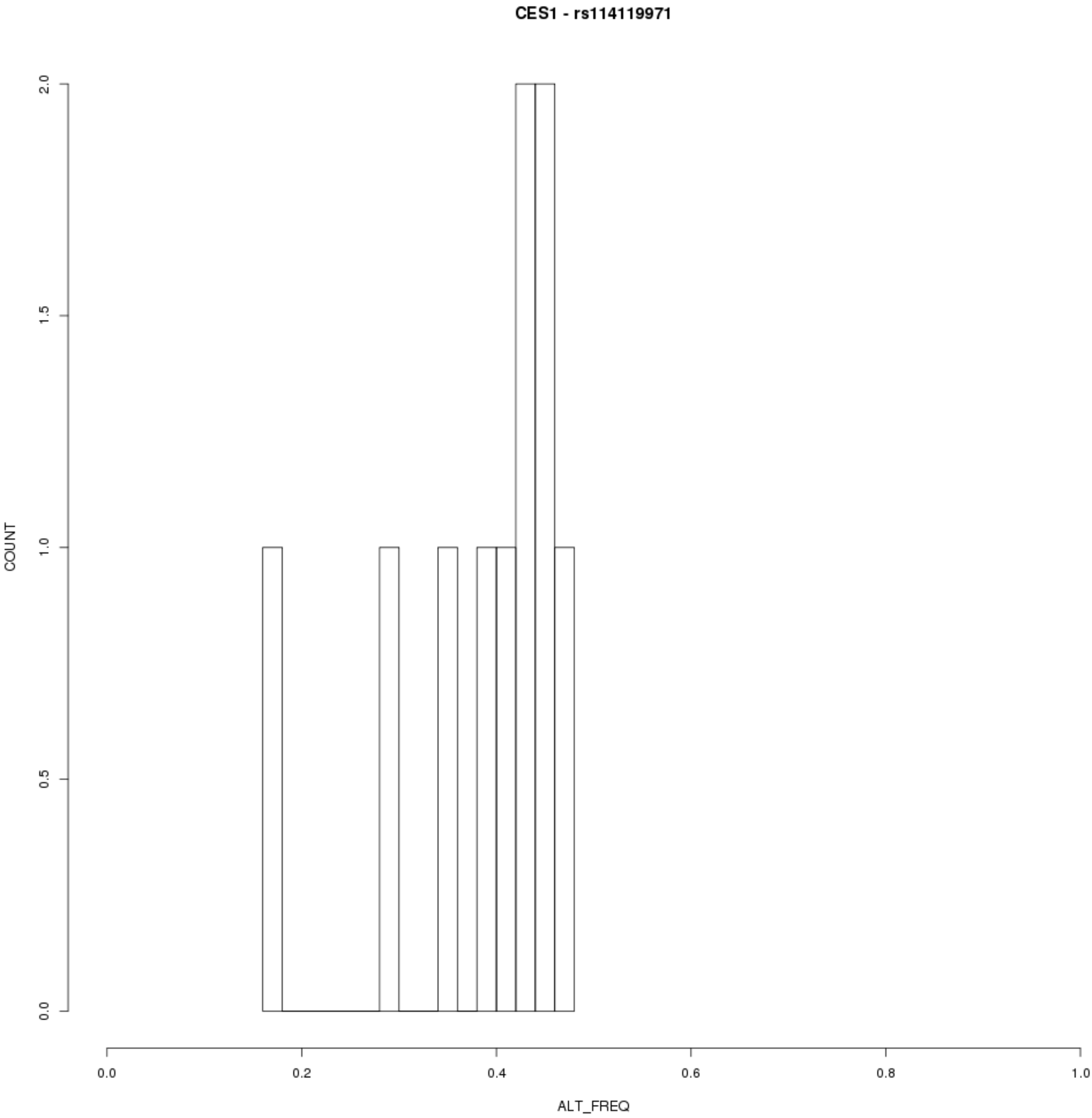
CES1 - rs114119971
COUNT
ALT_FREQ
0.0
0.5
1.0
1.5
2.0
0.0
0.2
0.4
0.6
0.8
1.0

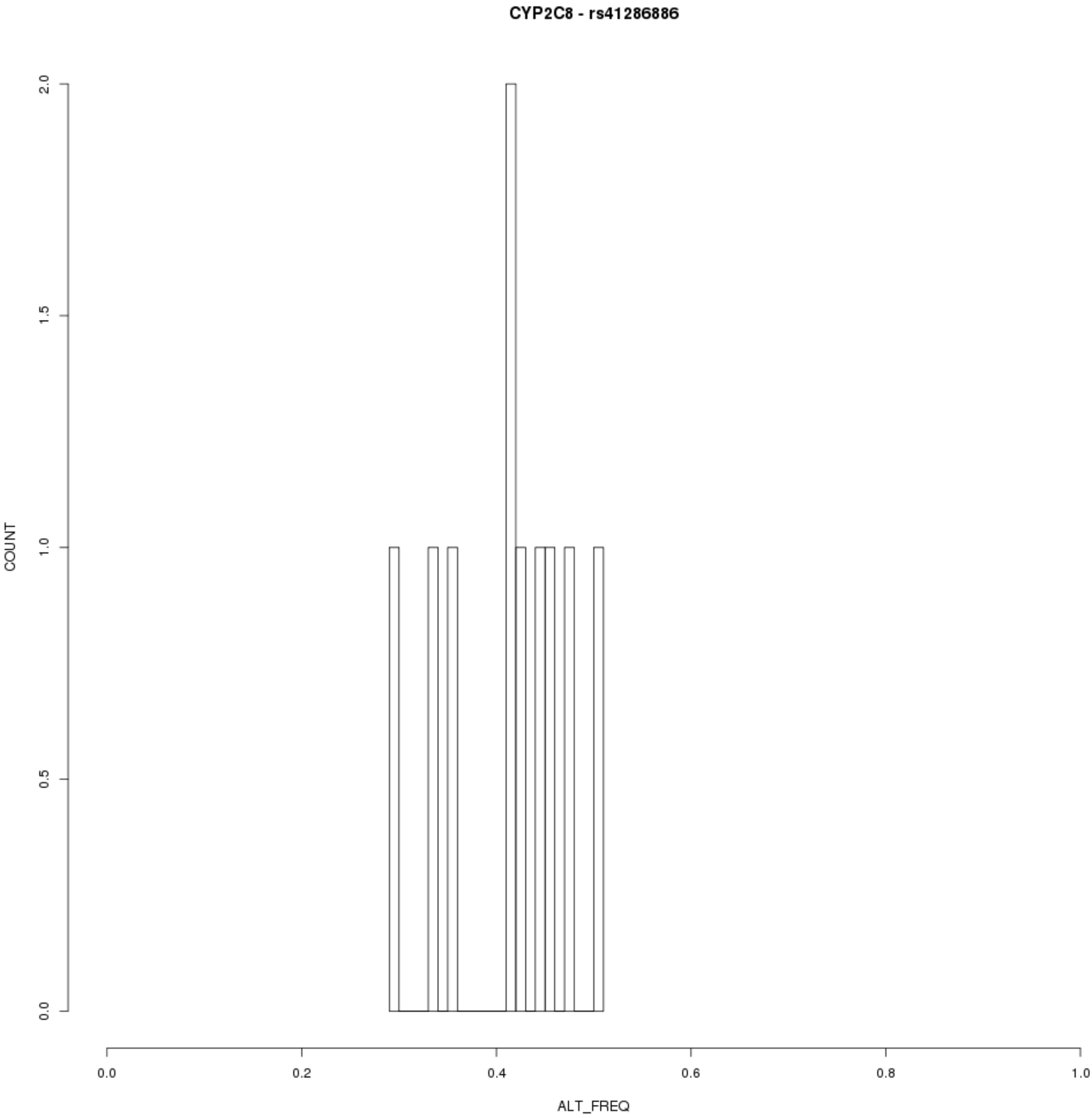

CYP2C8 - rs41286886
COUNT
ALT_FREQ
0.0
0.5
1.0
1.5
2.0
0.0
0.2
0.4
0.6
0.8
1.0

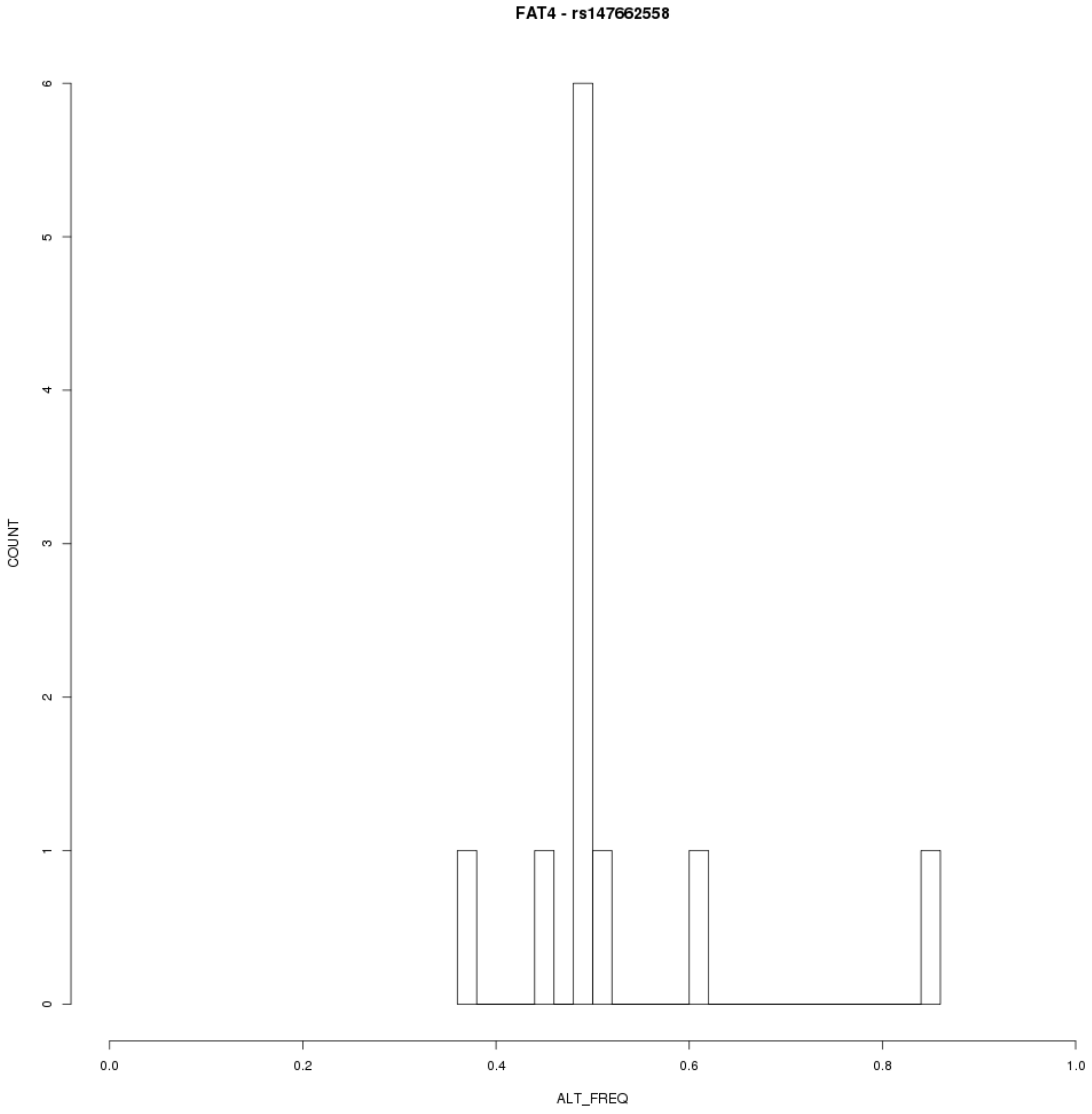

FAT4 - rs147662558
COUNT
ALT_FREQ
0
1
2
3
4
5
6
0.0
0.2
0.4
0.6
0.8
1.0

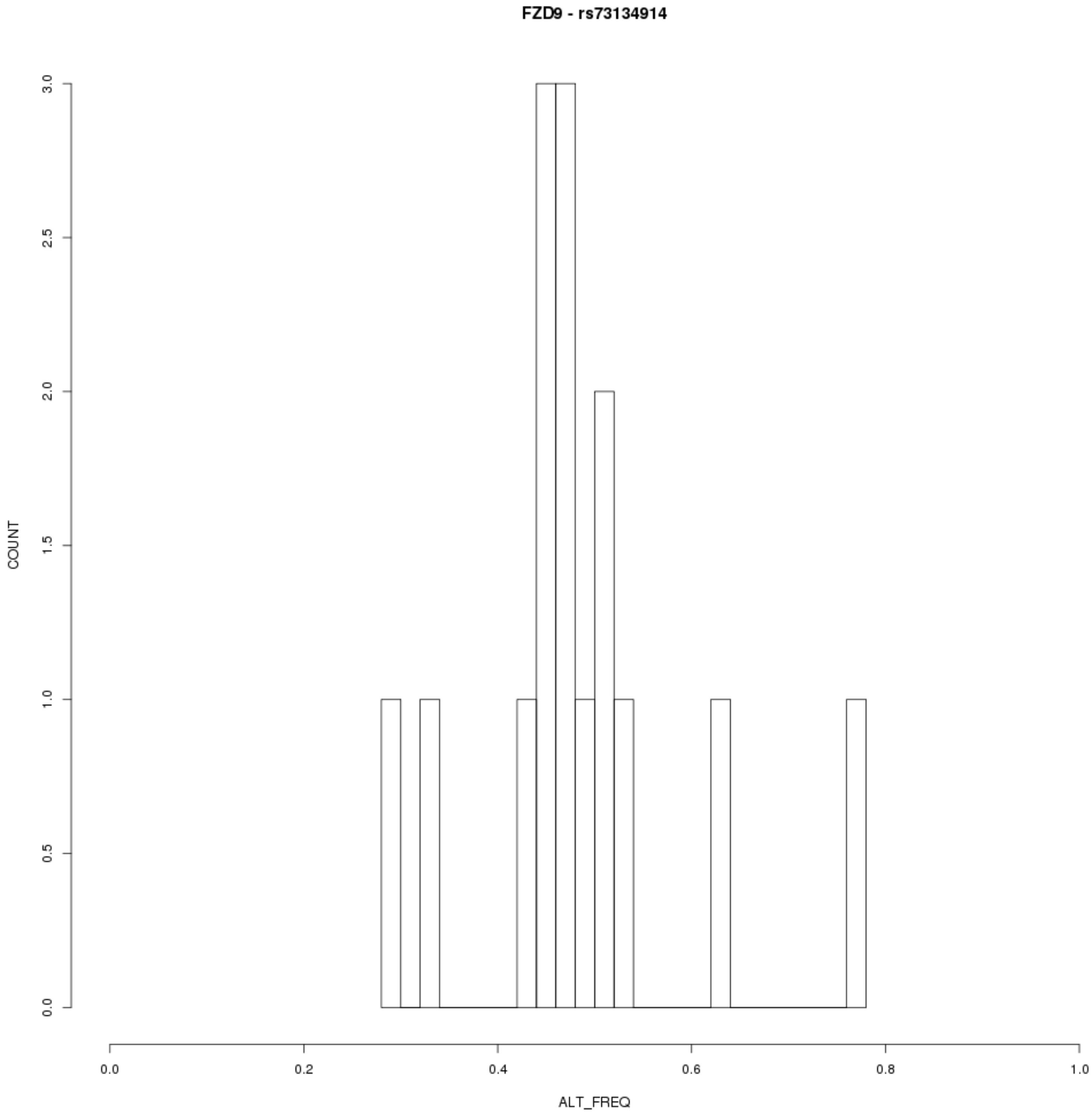

FZD9 - rs73134914
COUNT
ALT_FREQ
0.0
0.5
1.0
1.5
2.0
2.5
3.0
0.0
0.2
0.4
0.6
0.8
1.0

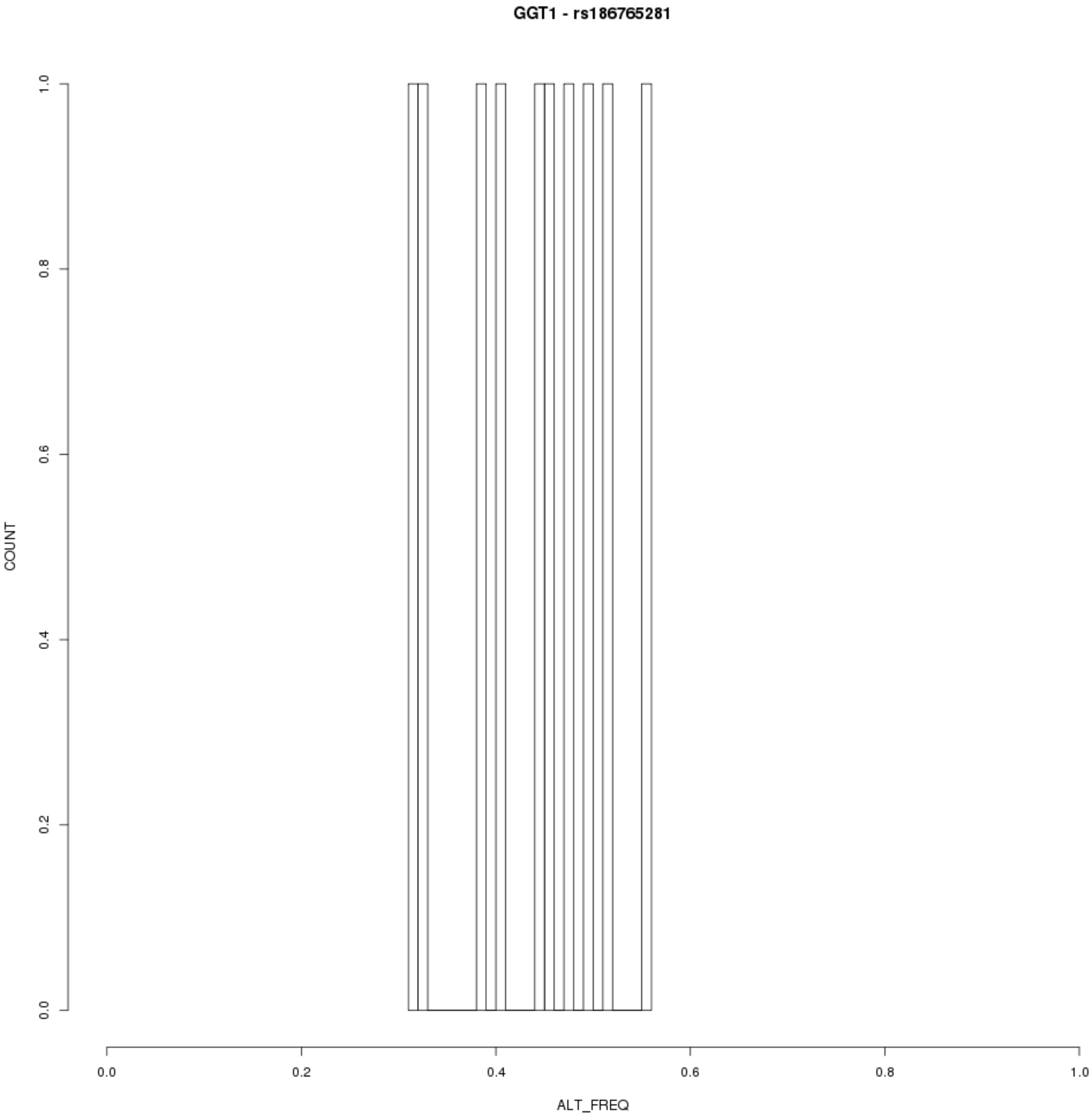
GGT1 - rs186765281
COUNT
ALT_FREQ
0.0
0.2
0.4
0.6
0.8
1.0

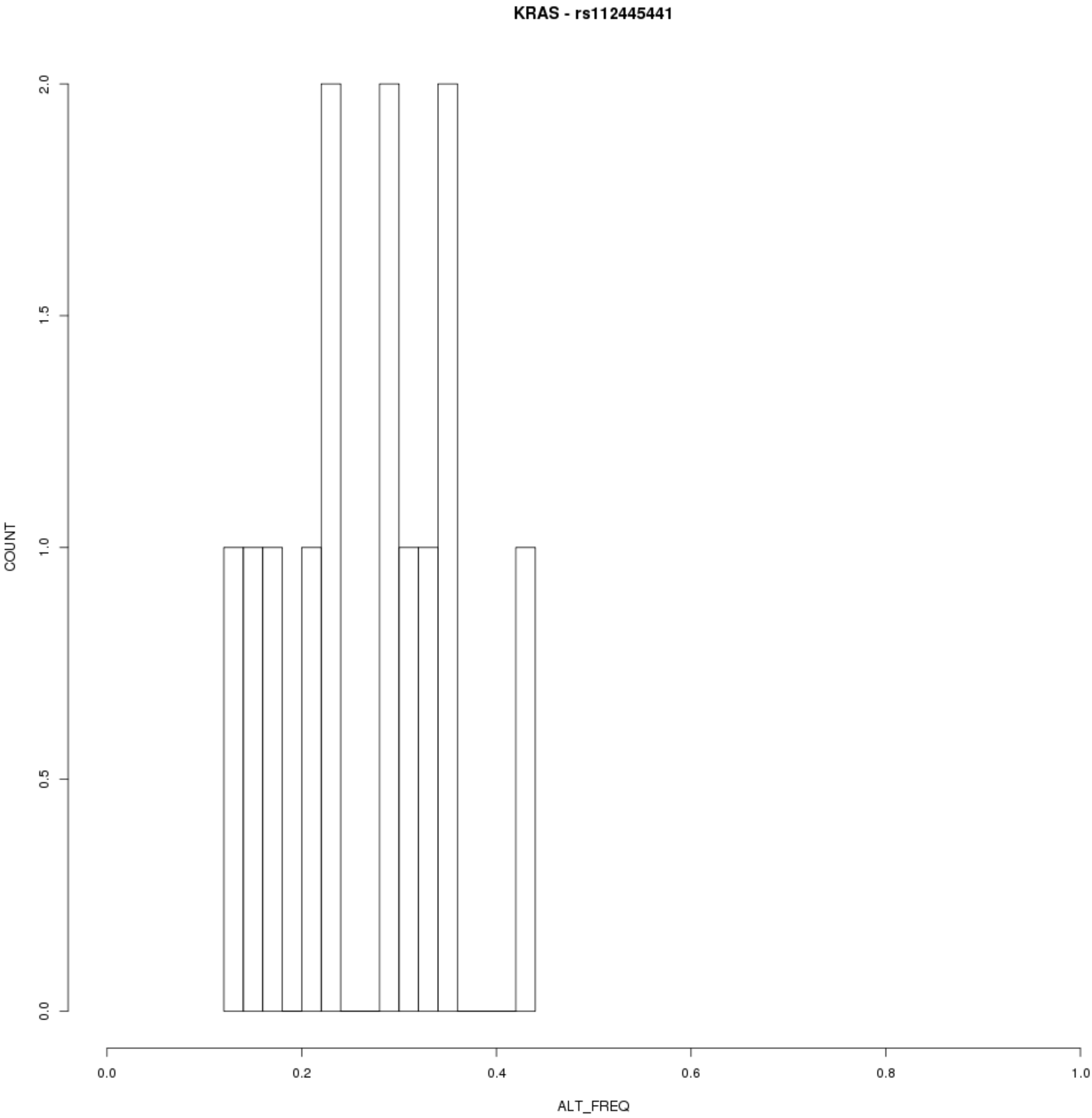
KRAS - rs112445441
COUNT
ALT_FREQ
0.0
0.5
1.0
1.5
2.0
0.0
0.2
0.4
0.6
0.8
1.0

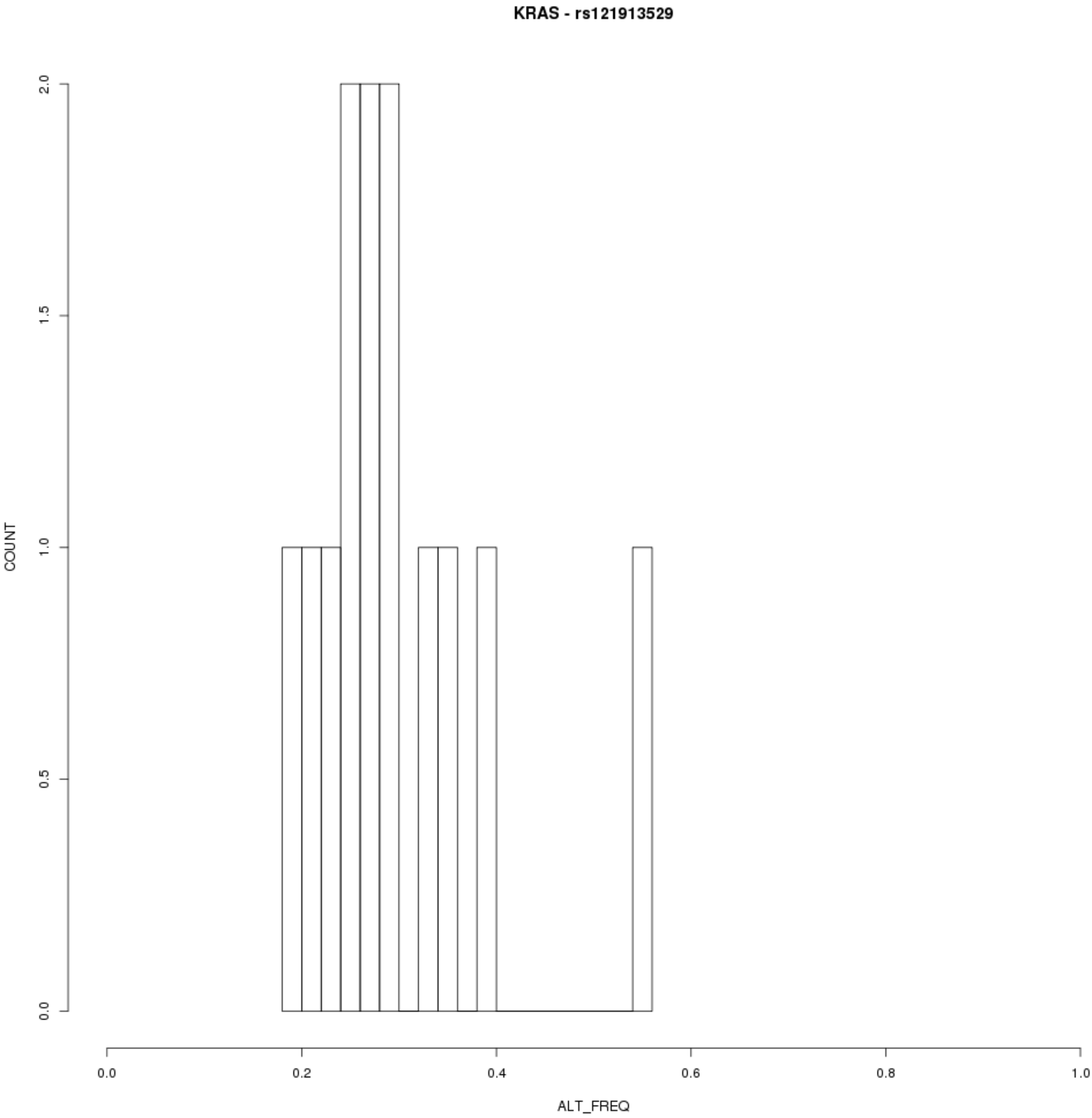
KRAS - rs121913529
COUNT
0.0
0.5
1.0
1.5
2.0
0.0
0.2
0.4
0.6
0.8
1.0
ALT_FREQ

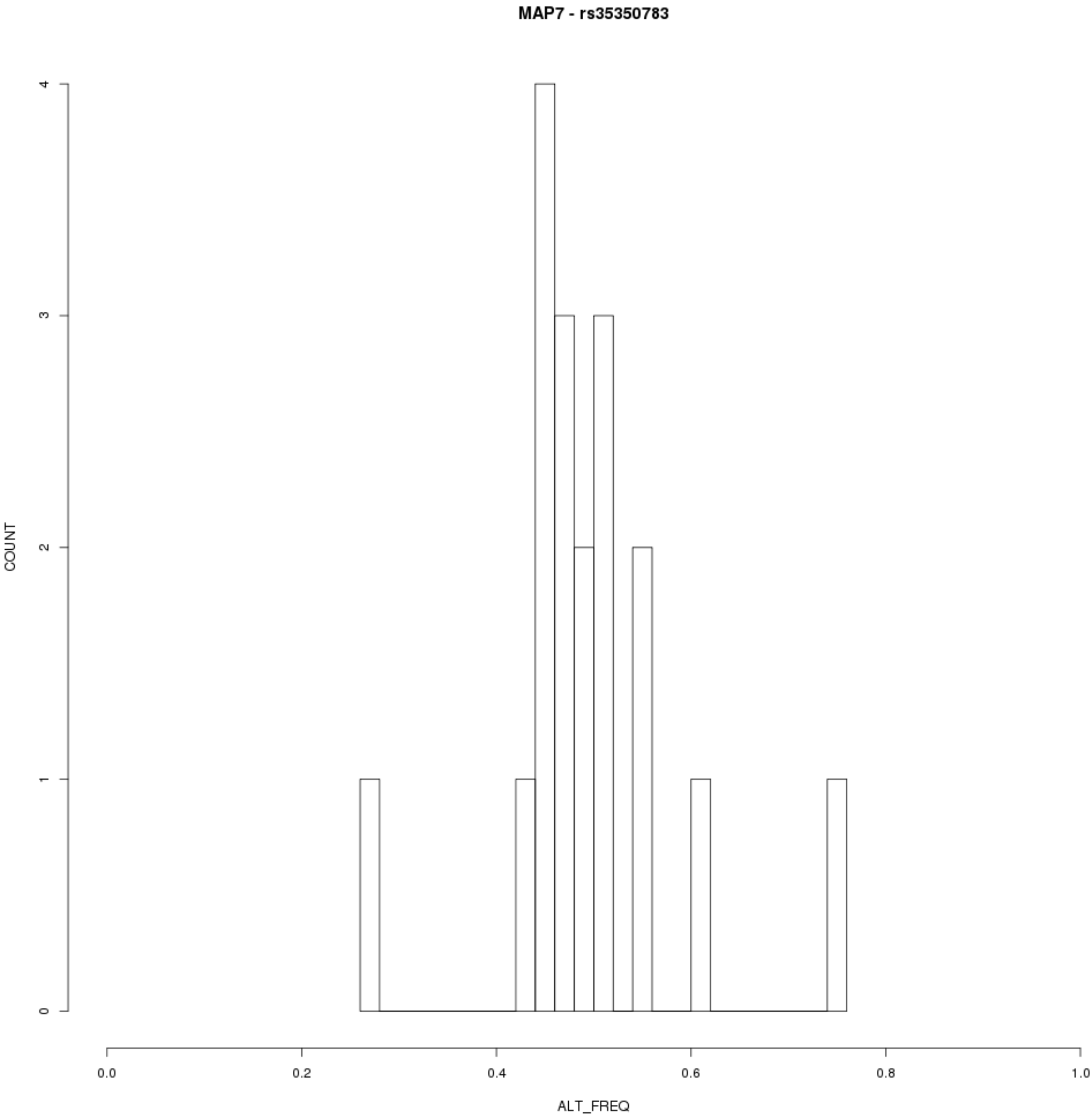
MAP7 - rs35350783
COUNT
ALT_FREQ
0
1
2
3
4
0.0
0.2
0.4
0.6
0.8
1.0

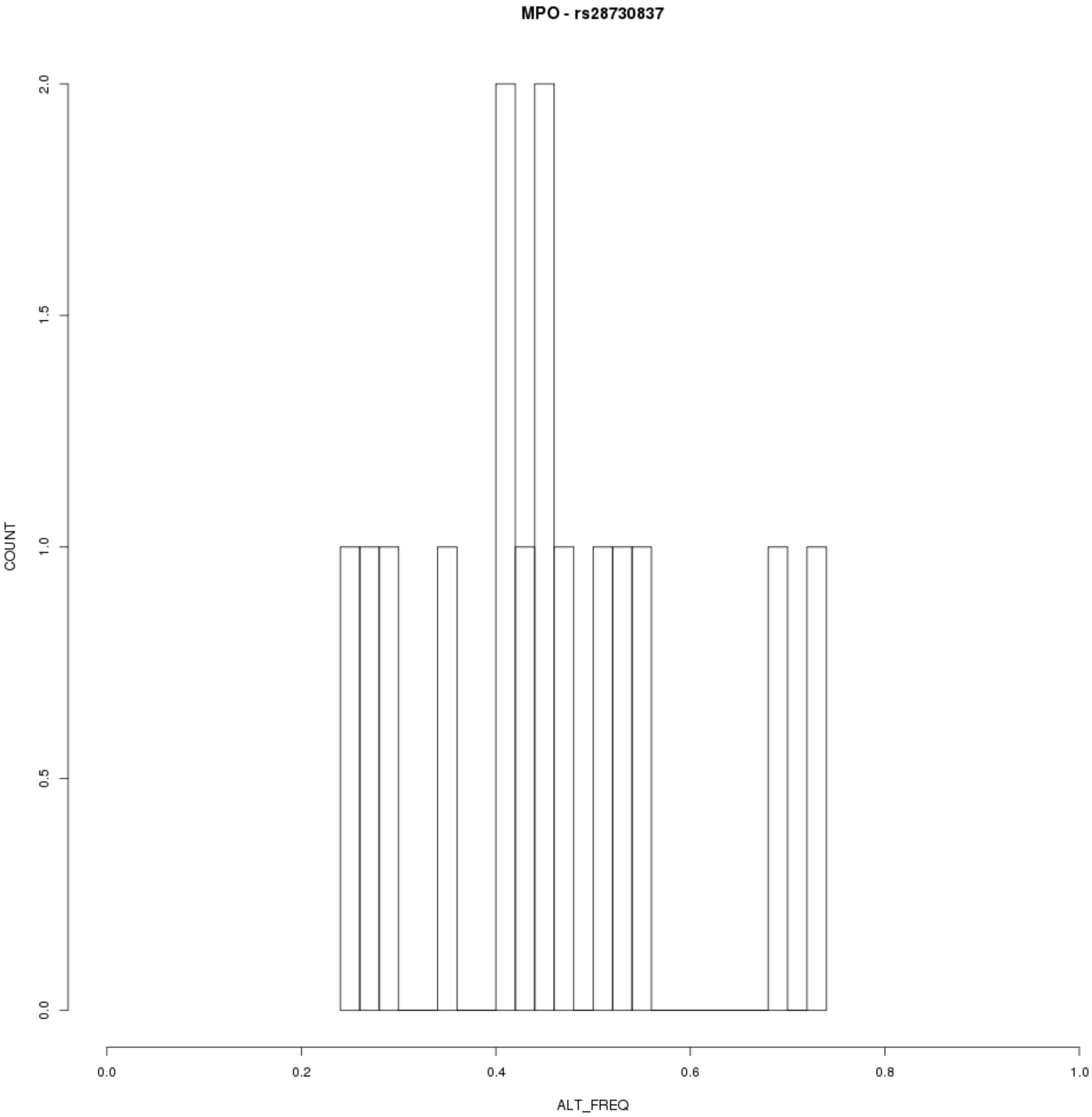
MPO - rs28730837
COUNT
ALT_FREQ
0.0
0.5
1.0
1.5
2.0
0.0
0.2
0.4
0.6
0.8
1.0

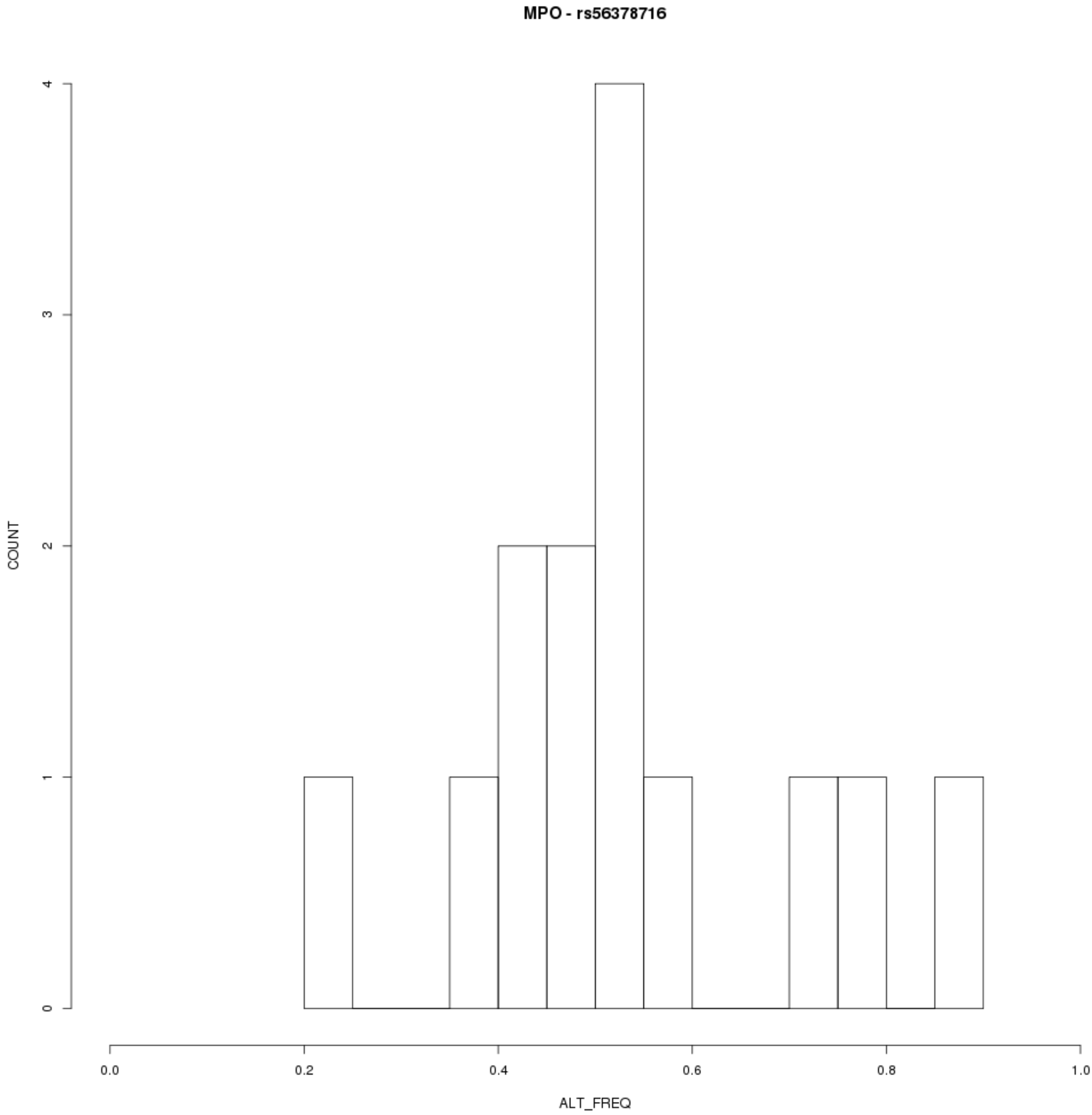
MPO - rs56378716
COUNT
ALT_FREQ
0
1
2
3
4
0.0
0.2
0.4
0.6
0.8
1.0

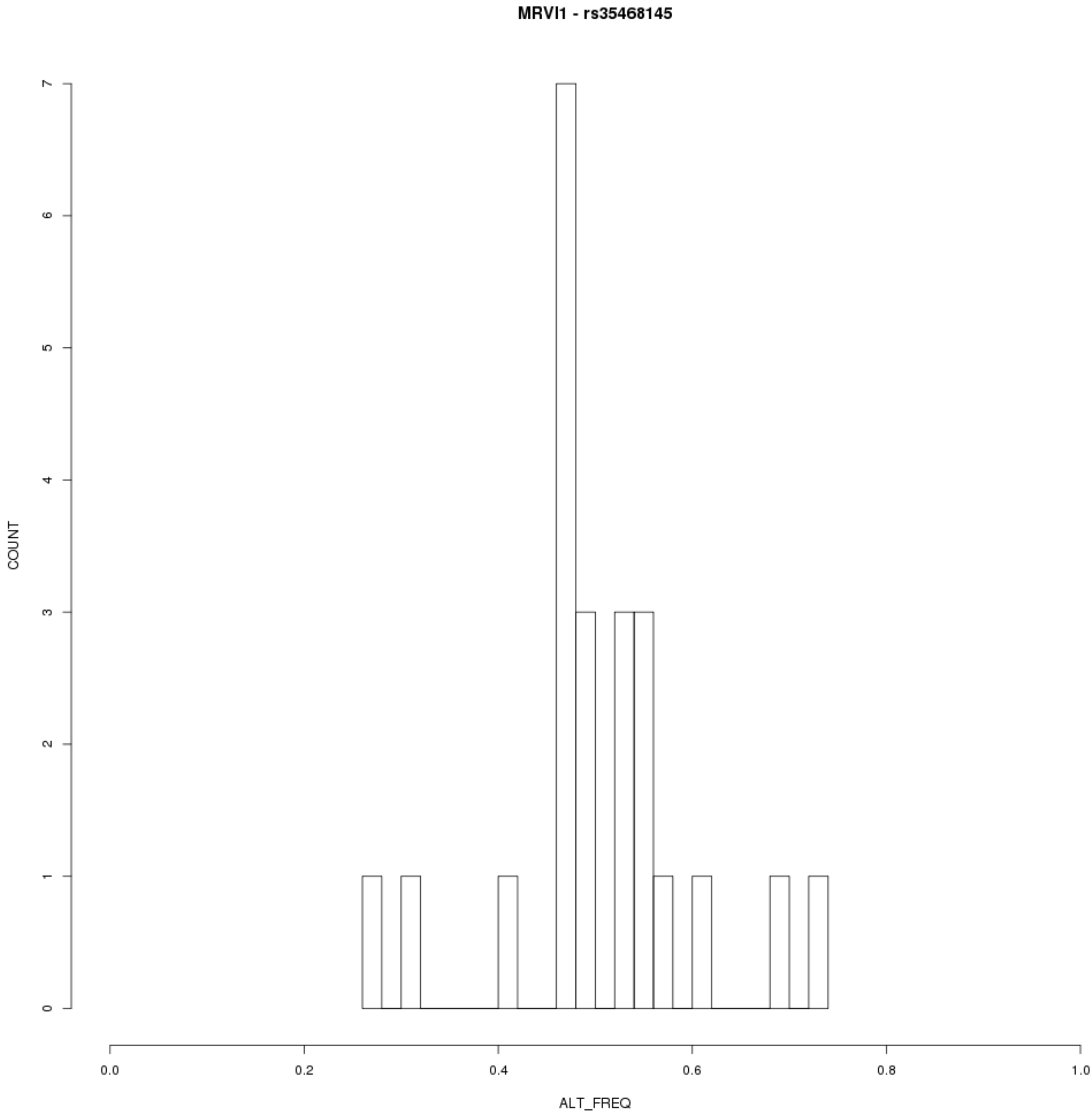

MRVI1 - rs35468145
COUNT
ALT_FREQ
0
1
2
3
4
5
6
7
0.0
0.2
0.4
0.6
0.8
1.0

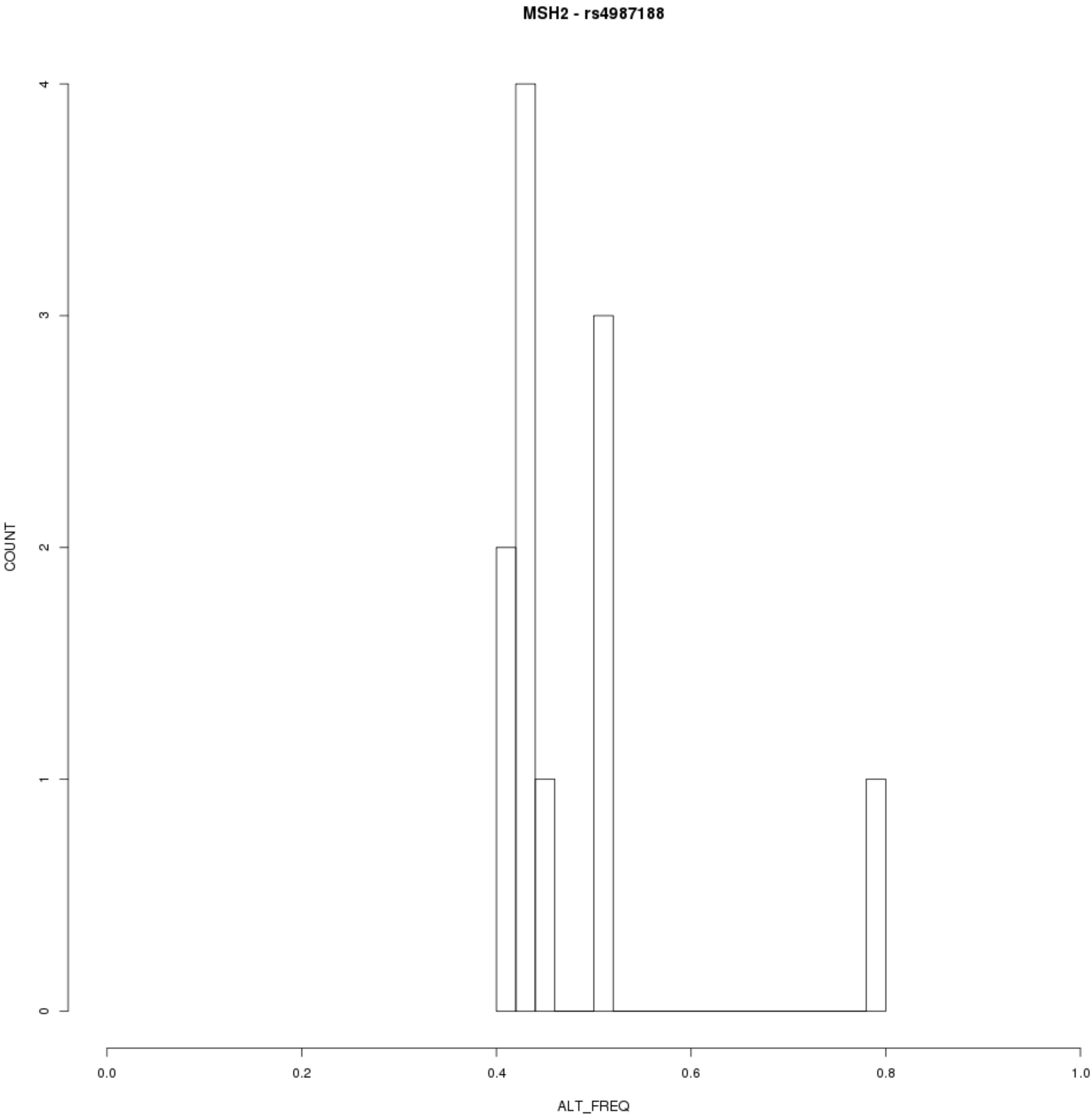
MSH2 - rs4987188
COUNT
0
1
2
3
4
ALT_FREQ
0.0
0.2
0.4
0.6
0.8
1.0

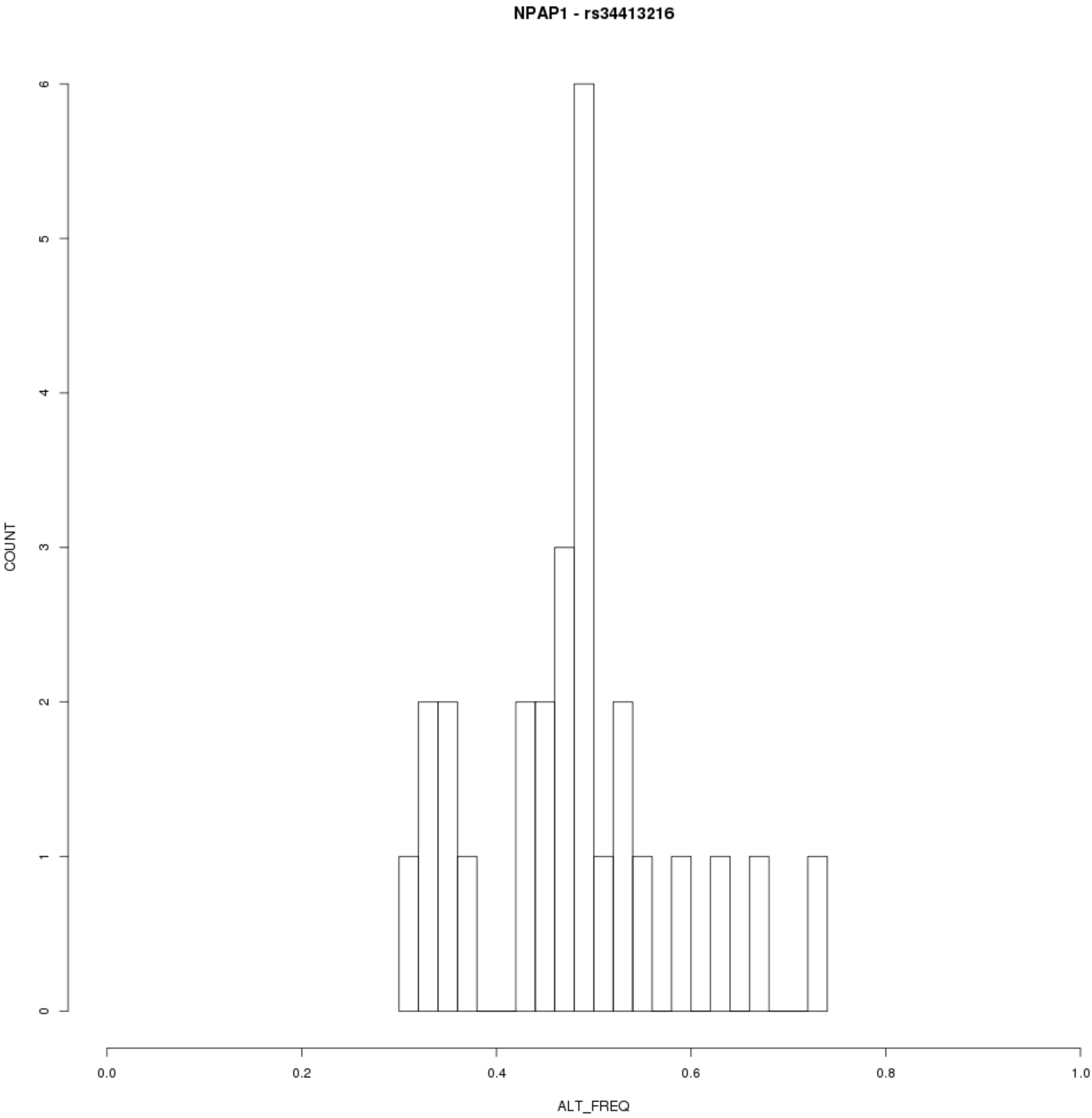

NPAP1 - rs34413216
COUNT
ALT_FREQ
0
1
2
3
4
5
6
0.0
0.2
0.4
0.6
0.8
1.0

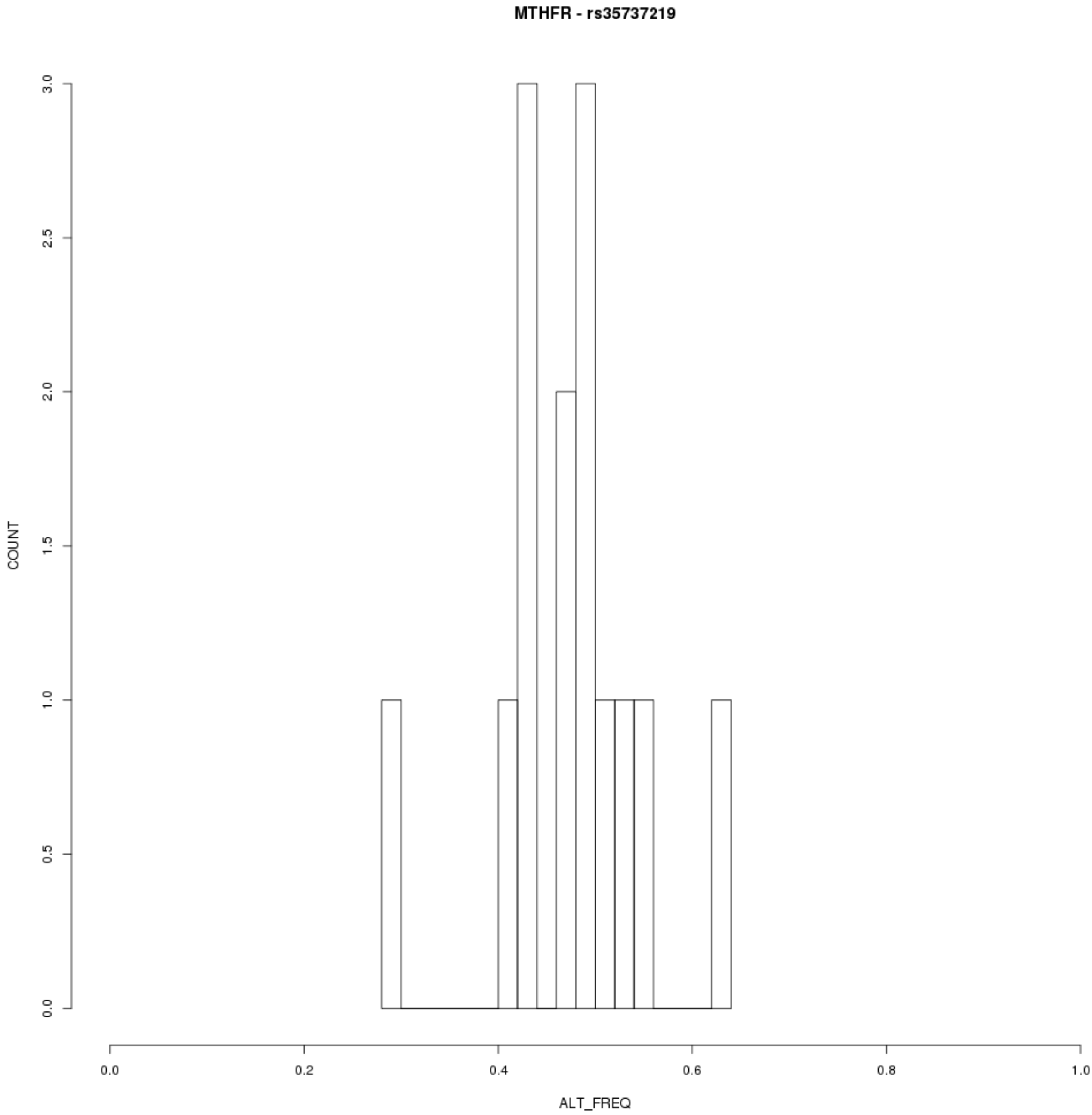
MTHFR - rs35737219
COUNT
ALT_FREQ
0.0
0.5
1.0
1.5
2.0
2.5
3.0
0.0
0.2
0.4
0.6
0.8
1.0

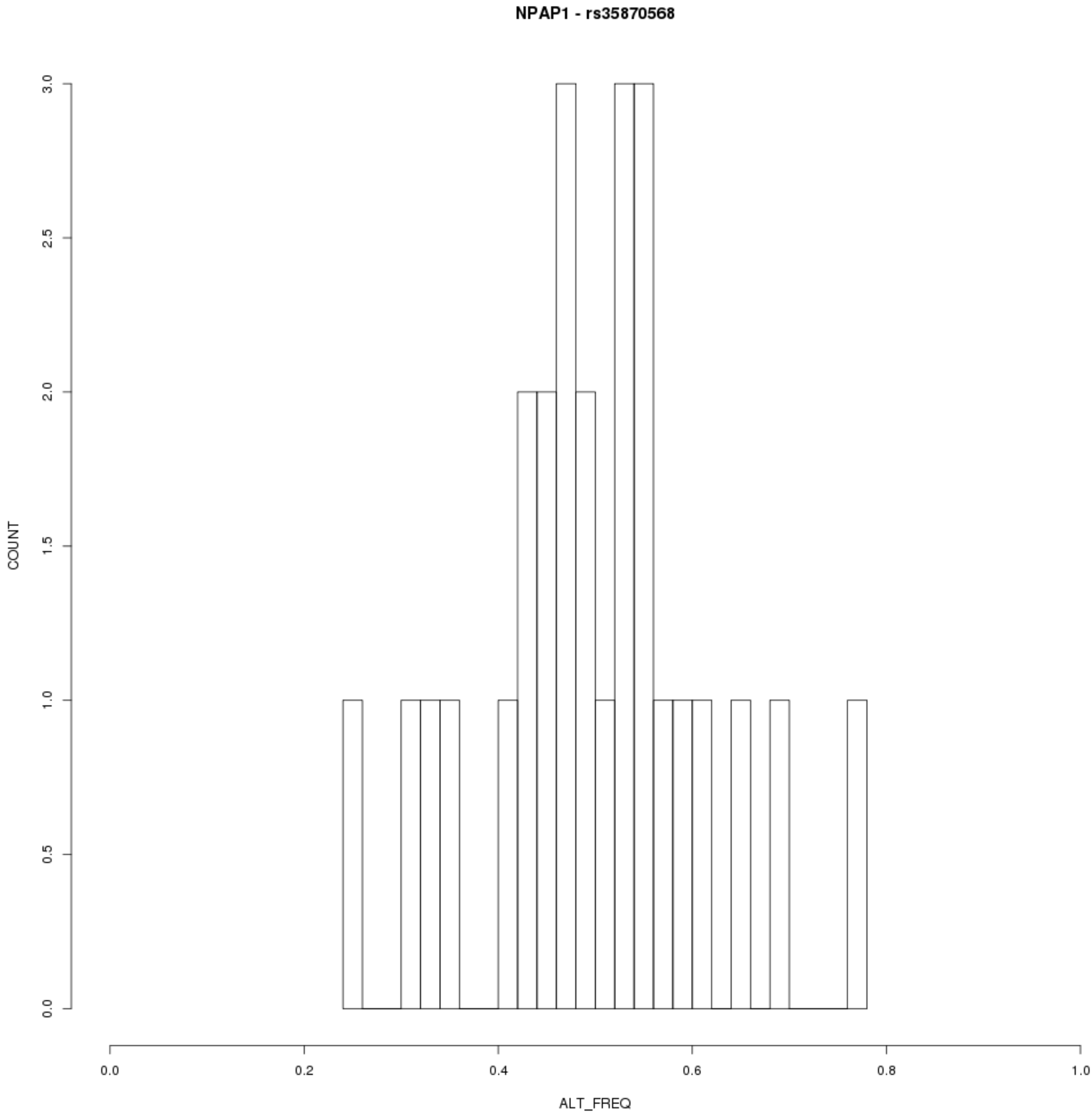

NPAP1 - rs35870568
COUNT
ALT_FREQ
0.0
0.5
1.0
1.5
2.0
2.5
3.0
0.0
0.2
0.4
0.6
0.8
1.0

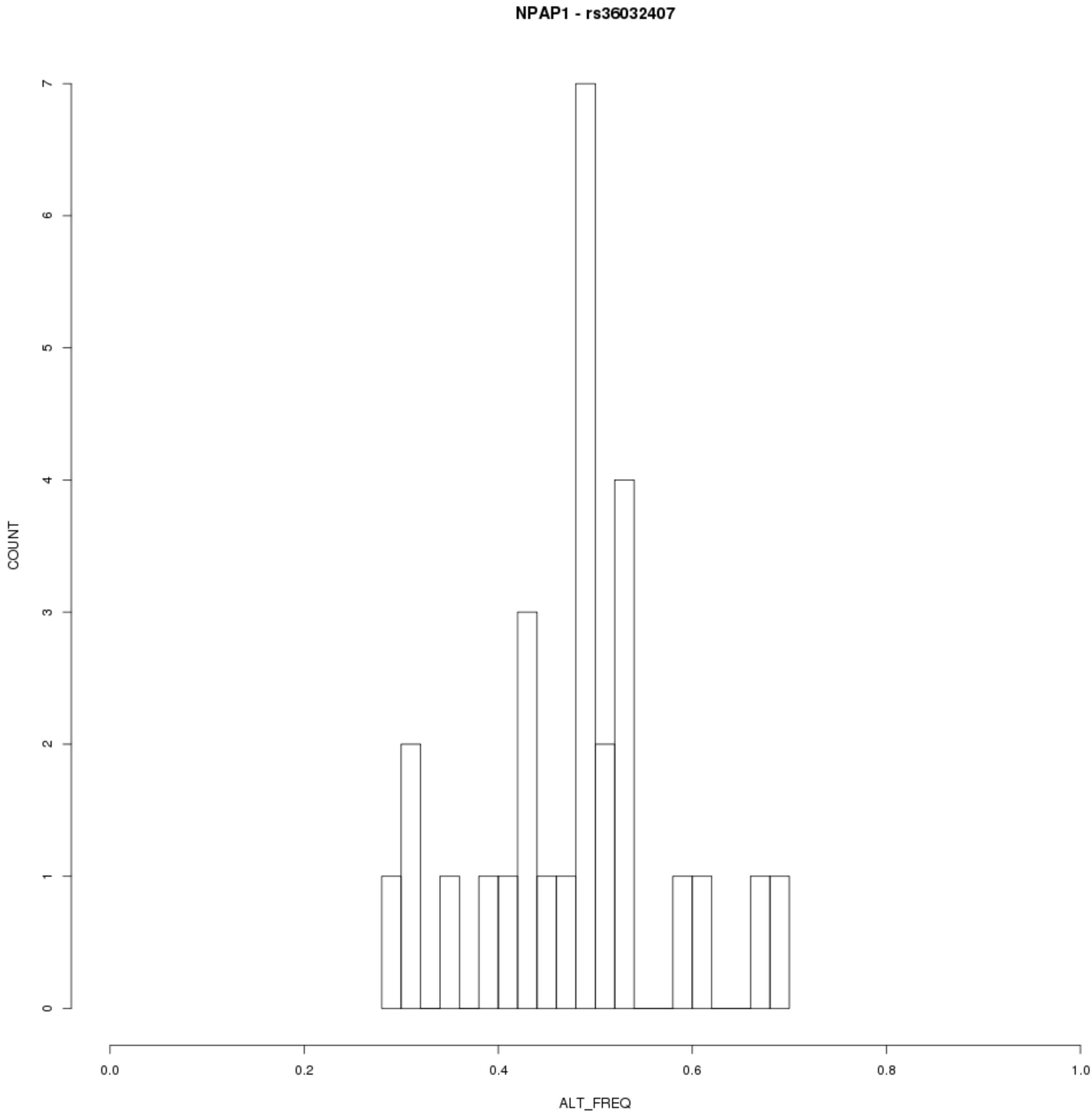

NPAP1 - rs36032407
COUNT
ALT_FREQ
0
1
2
3
4
5
6
7
0.0
0.2
0.4
0.6
0.8
1.0

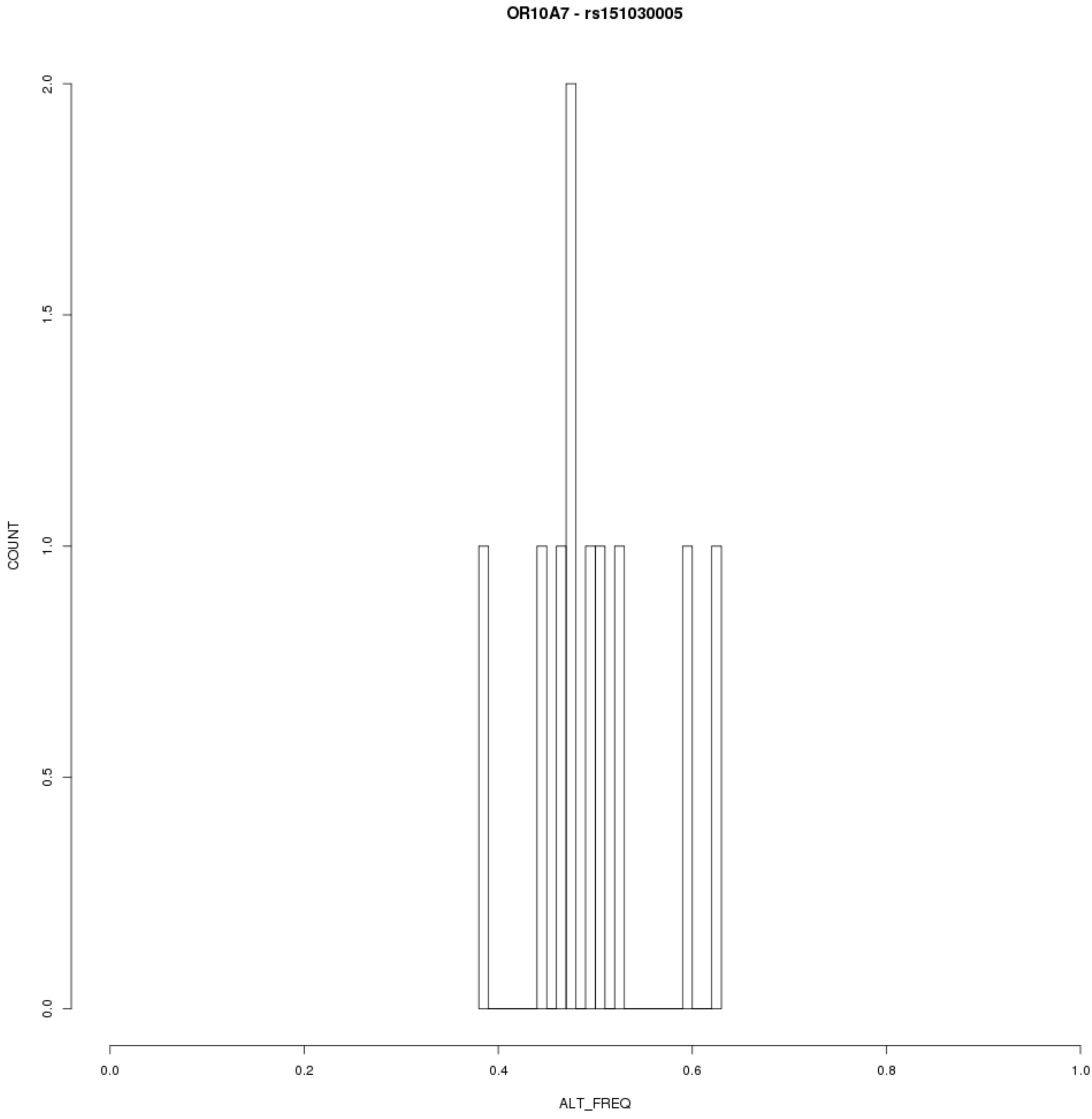

OR10A7 - rs151030005
COUNT
0.0
0.5
1.0
1.5
2.0
0.0
0.2
0.4
0.6
0.8
1.0
ALT_FREQ

OR2L13 - rs45577033

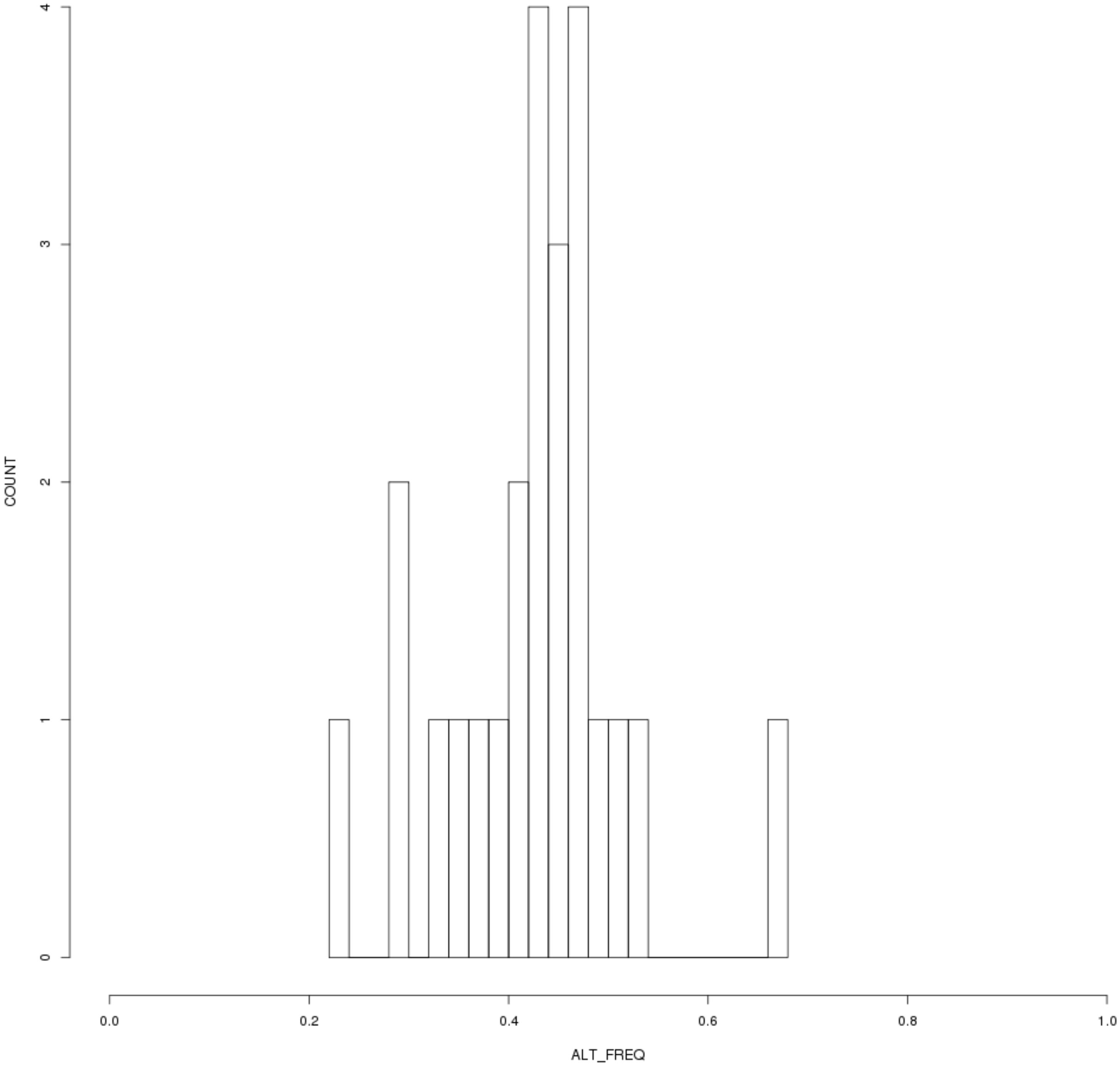

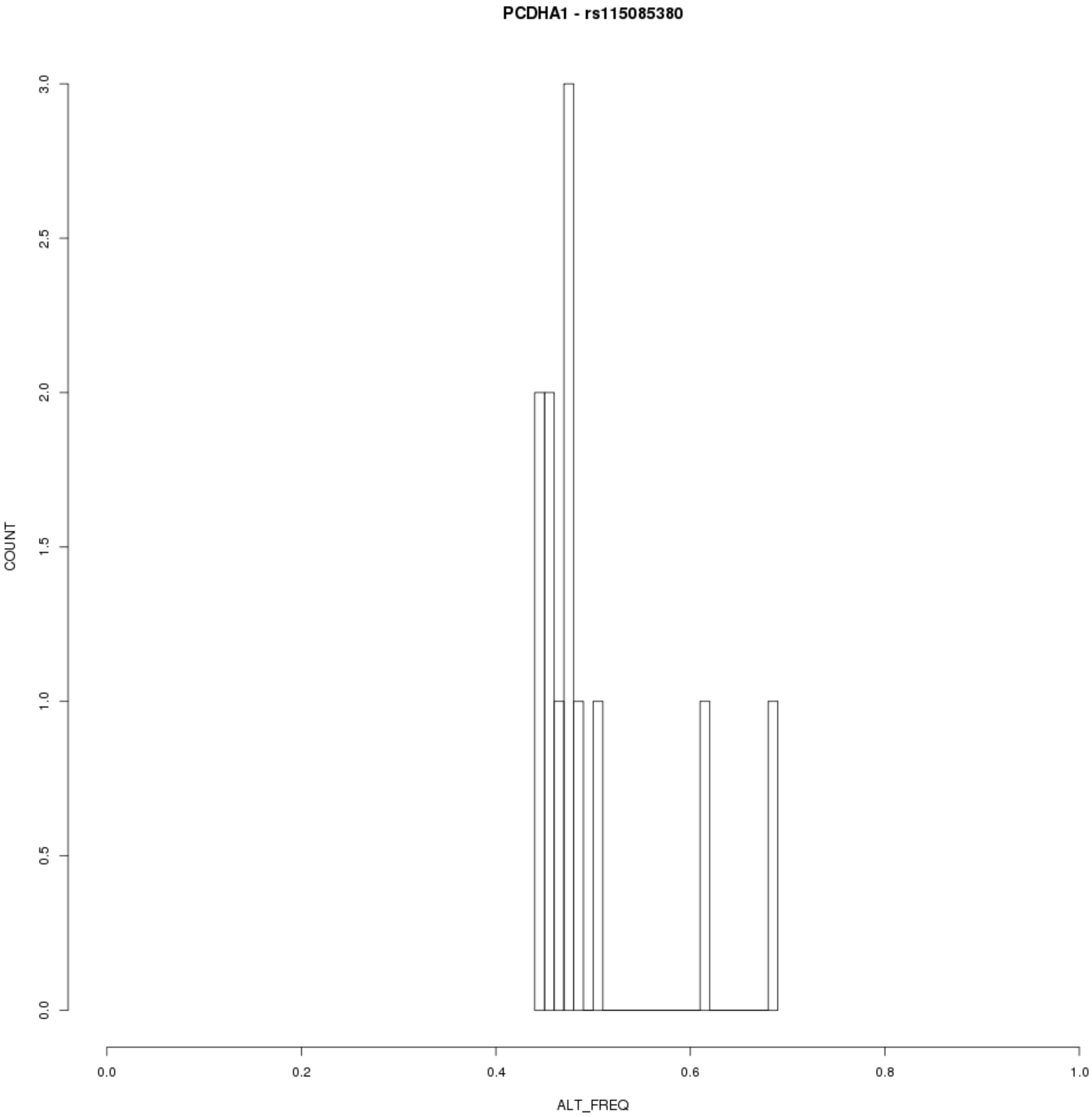
PCDHA1 - rs115085380
COUNT
ALT_FREQ
0.0
0.5
1.0
1.5
2.0
2.5
3.0
0.0
0.2
0.4
0.6
0.8
1.0

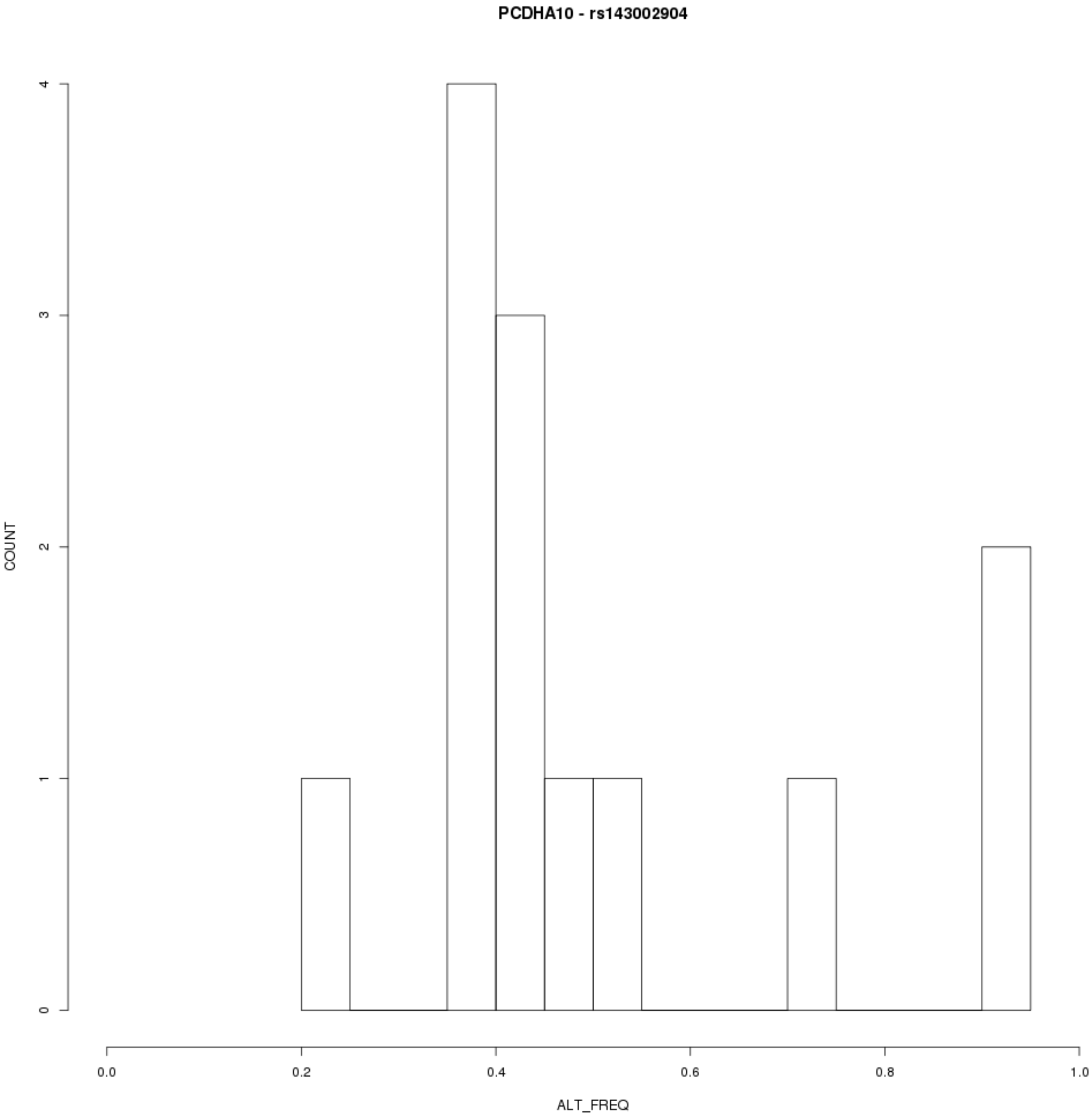
PCDHA10 - rs143002904
COUNT
ALT_FREQ
0
1
2
3
4
0.0
0.2
0.4
0.6
0.8
1.0

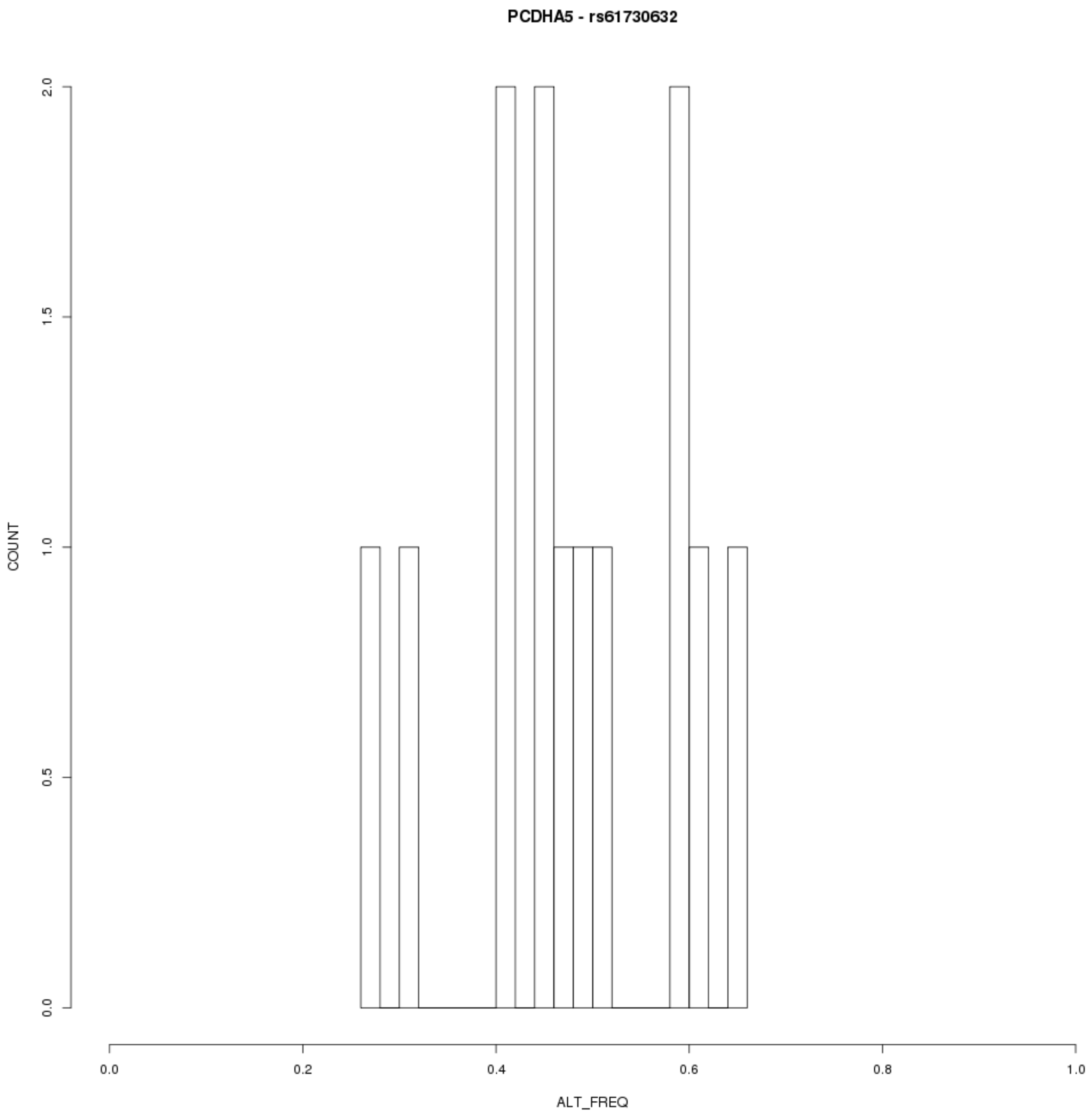
PCDHA5 - rs61730632
COUNT
ALT_FREQ
0.0
0.5
1.0
1.5
2.0
0.0
0.2
0.4
0.6
0.8
1.0

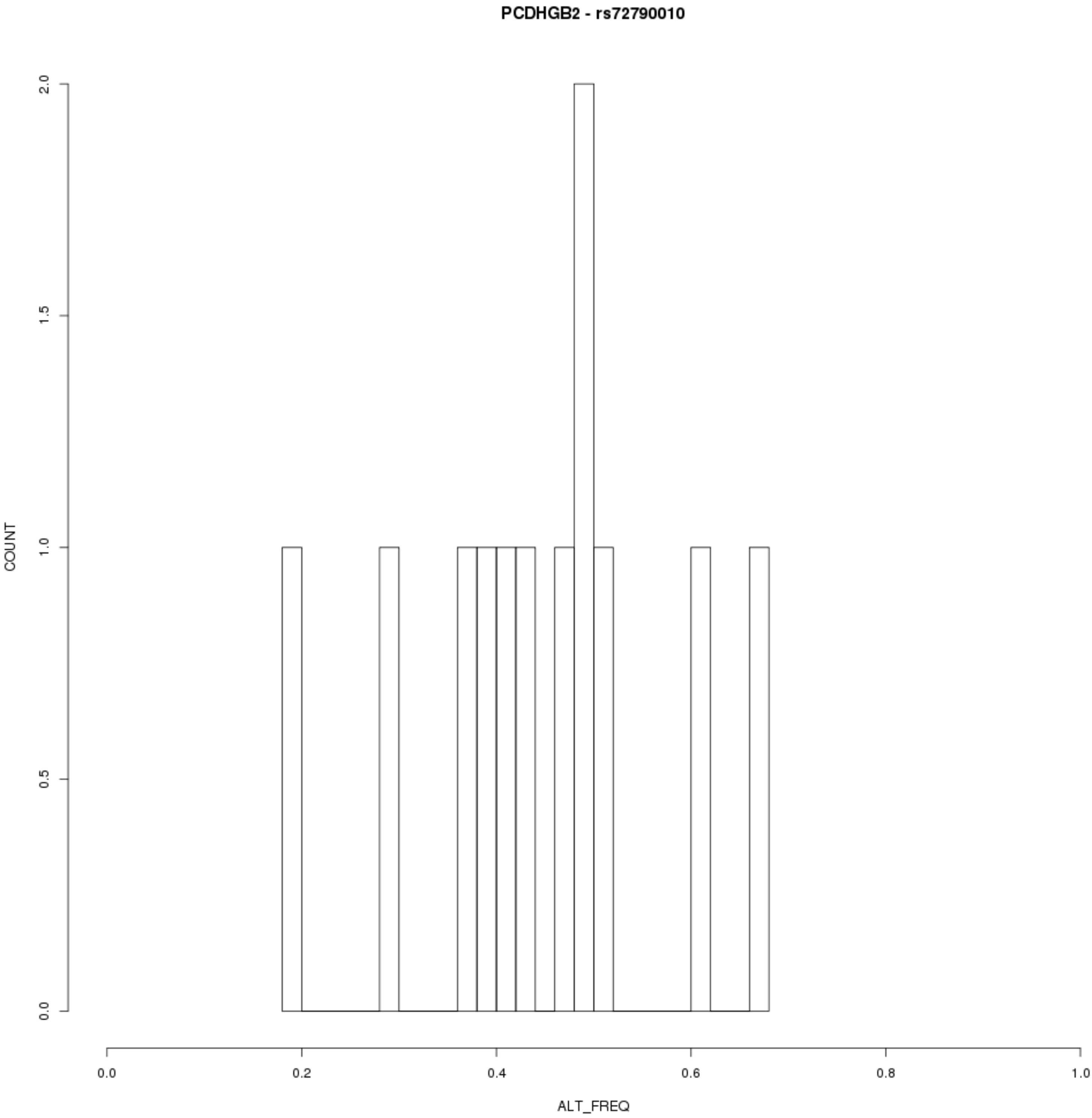
PCDHGB2 - rs72790010
COUNT
0.0
0.5
1.0
1.5
2.0
0.0
0.2
0.4
0.6
0.8
1.0
ALT_FREQ

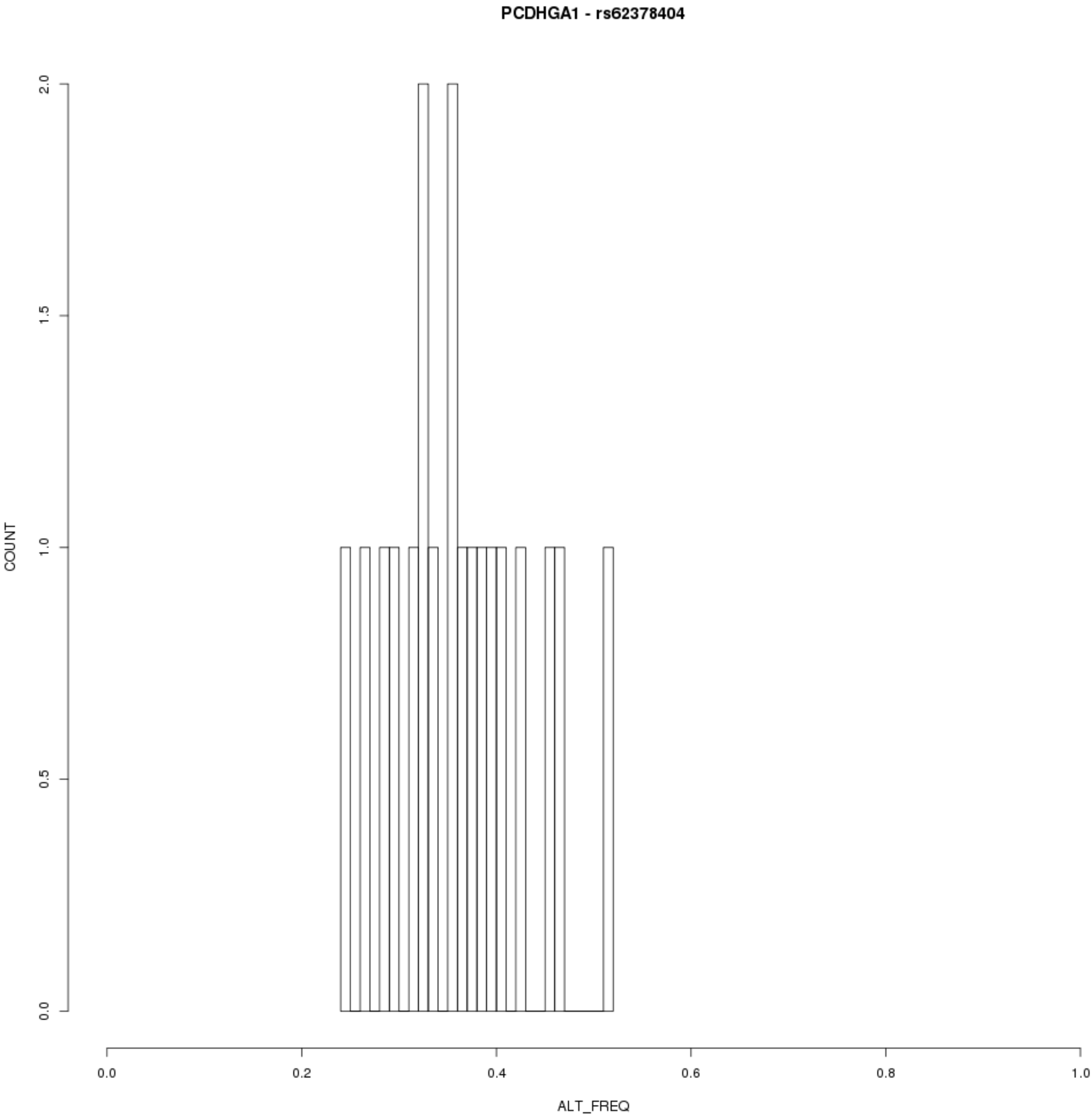

PCDHGA1 - rs62378404
COUNT
ALT_FREQ
0.0
0.5
1.0
1.5
2.0
0.0
0.2
0.4
0.6
0.8
1.0

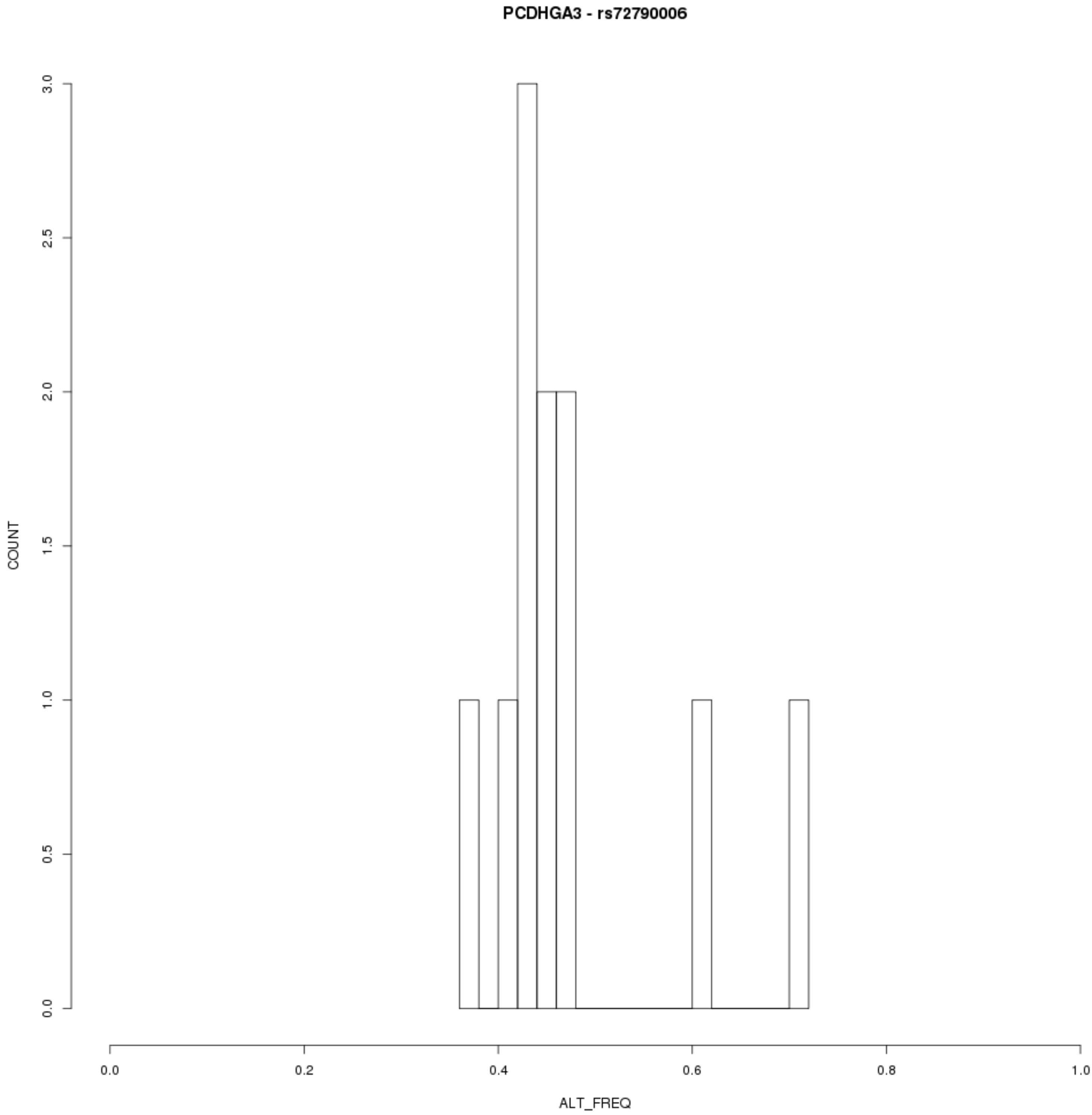
PCDHGA3 - rs72790006
COUNT
ALT_FREQ
0.0
0.5
1.0
1.5
2.0
2.5
3.0
0.0
0.2
0.4
0.6
0.8
1.0

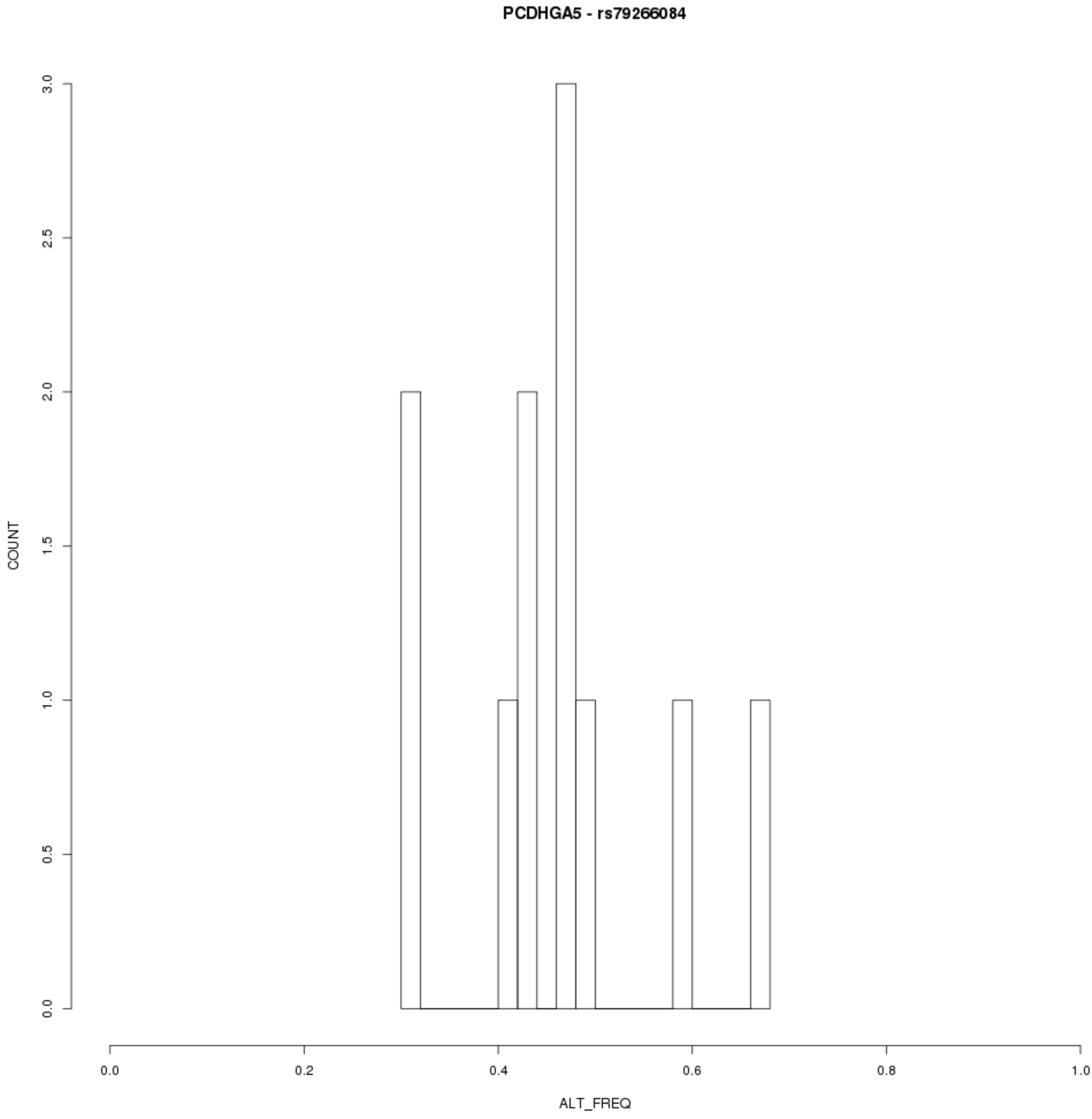

PCDHGA5 - rs79266084
COUNT
ALT_FREQ
0.0
0.5
1.0
1.5
2.0
2.5
3.0
0.0
0.2
0.4
0.6
0.8
1.0

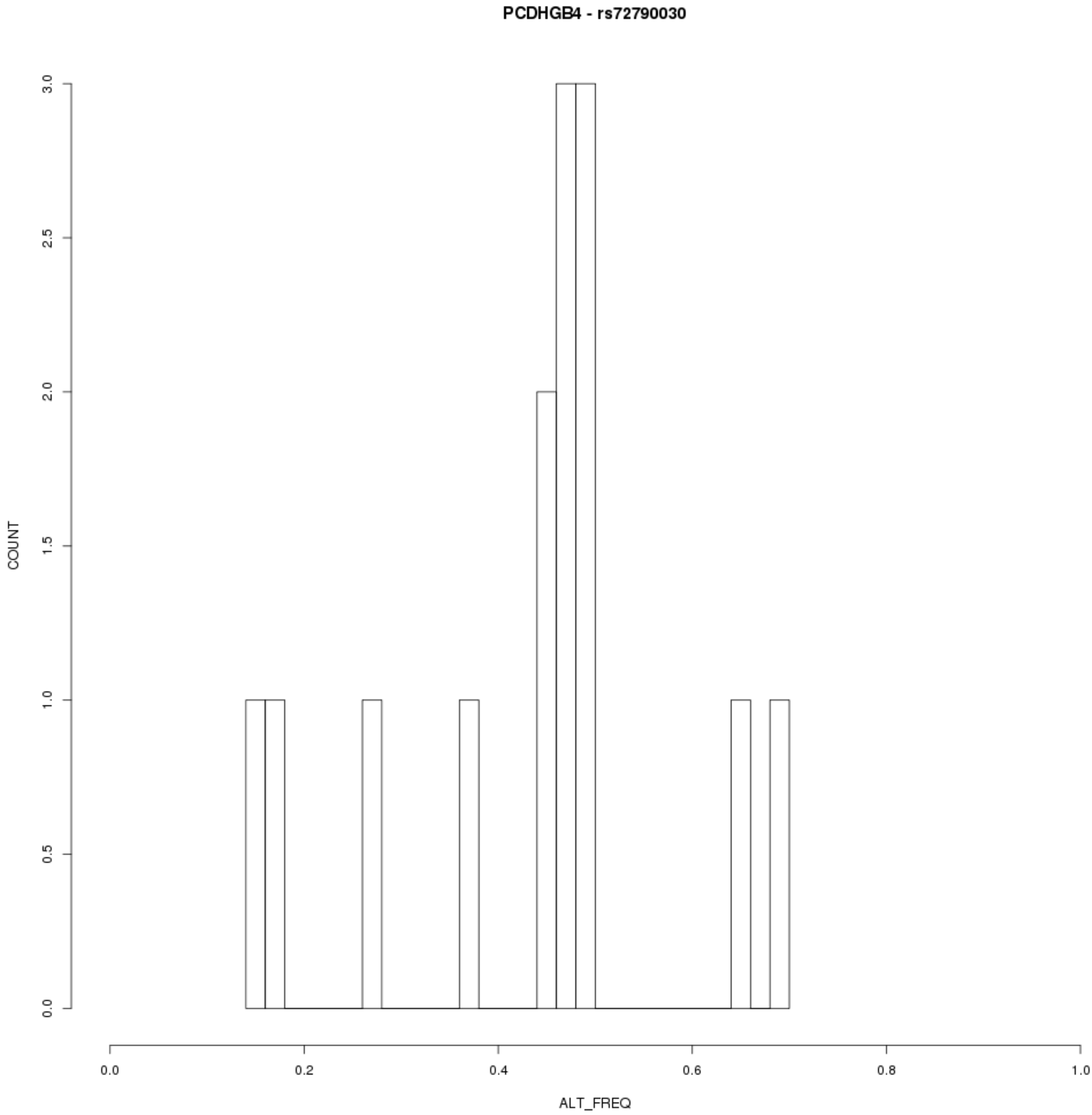
PCDHGB4 - rs72790030
COUNT
ALT_FREQ
0.0
0.5
1.0
1.5
2.0
2.5
3.0
0.0
0.2
0.4
0.6
0.8
1.0

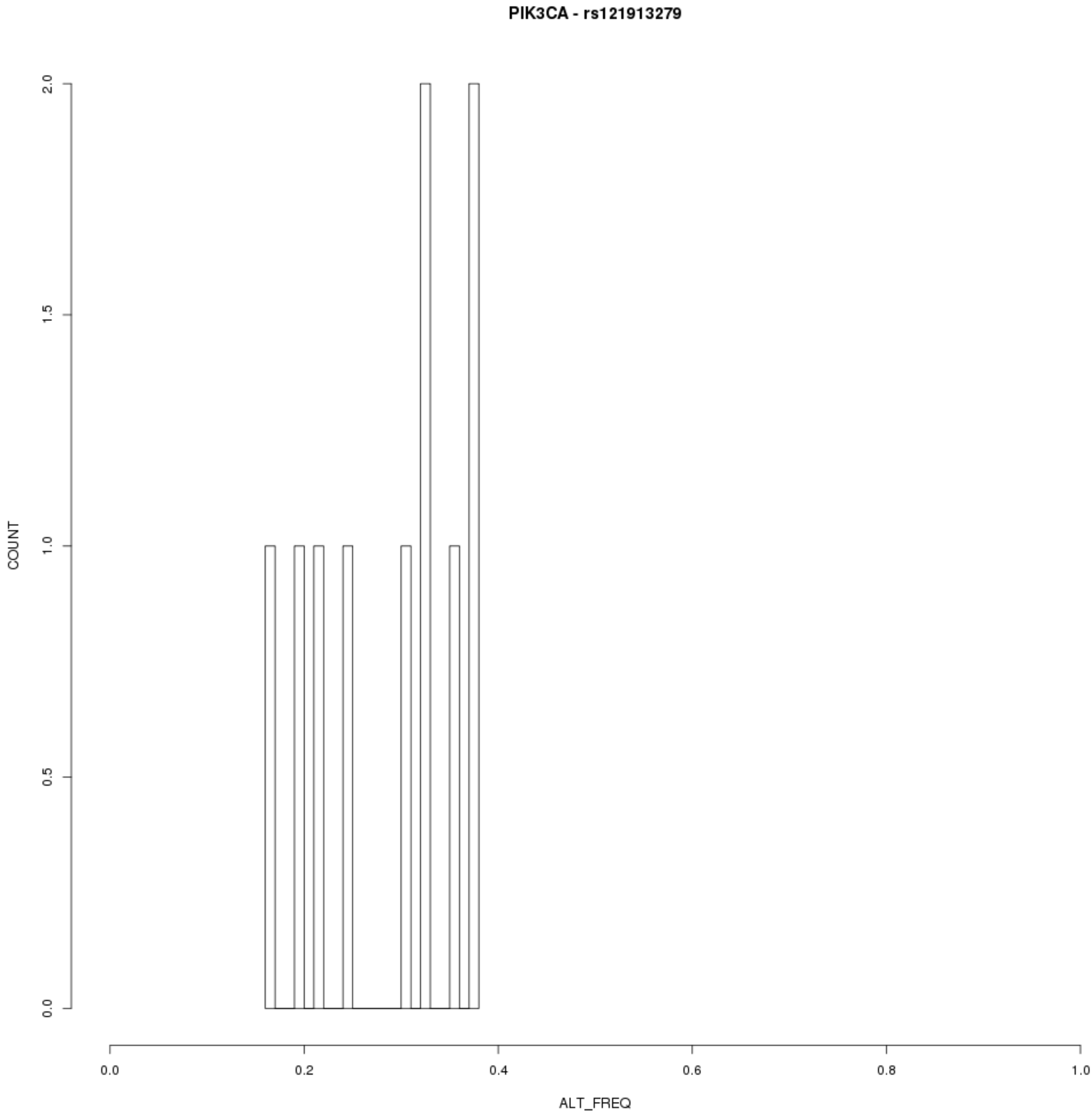
PIK3CA - rs121913279
COUNT
0.0
0.5
1.0
1.5
2.0
0.0
0.2
0.4
0.6
0.8
1.0
ALT_FREQ

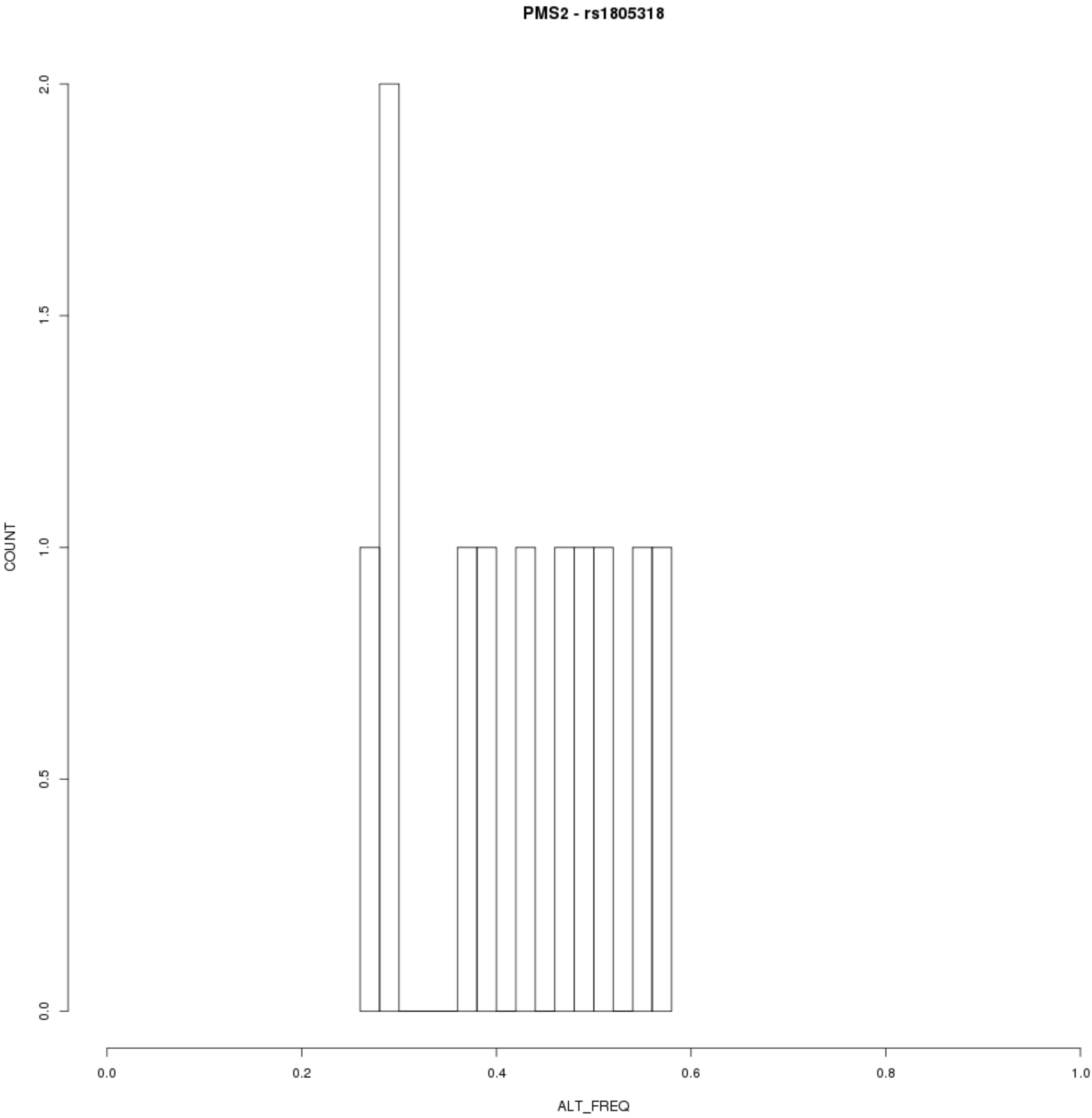
PMS2 - rs1805318
COUNT
0.0
0.5
1.0
1.5
2.0
0.0
0.2
0.4
0.6
0.8
1.0
ALT_FREQ

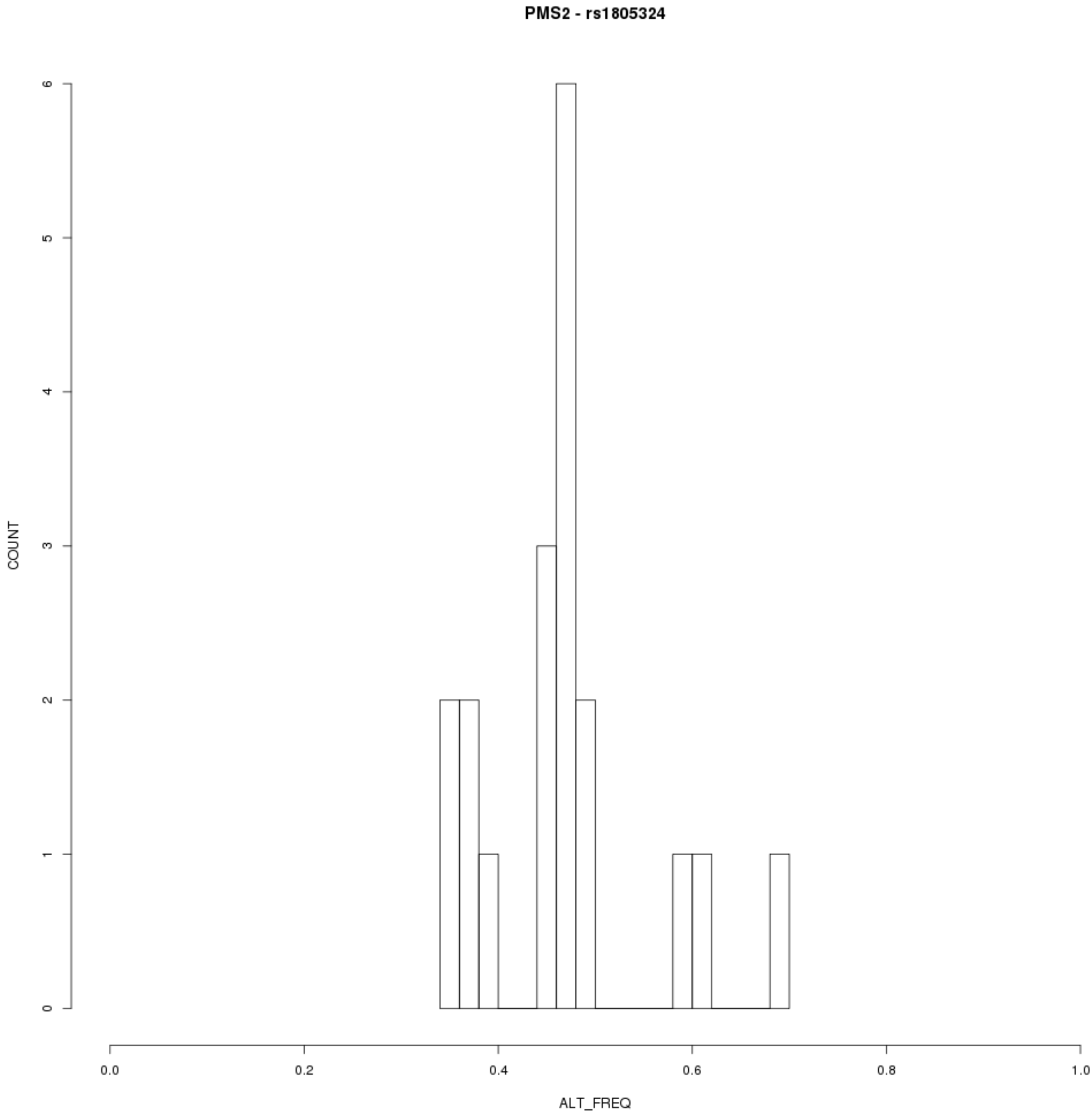
PMS2 - rs1805324
COUNT
0
1
2
3
4
5
6
0.0
0.2
0.4
0.6
0.8
1.0
ALT_FREQ

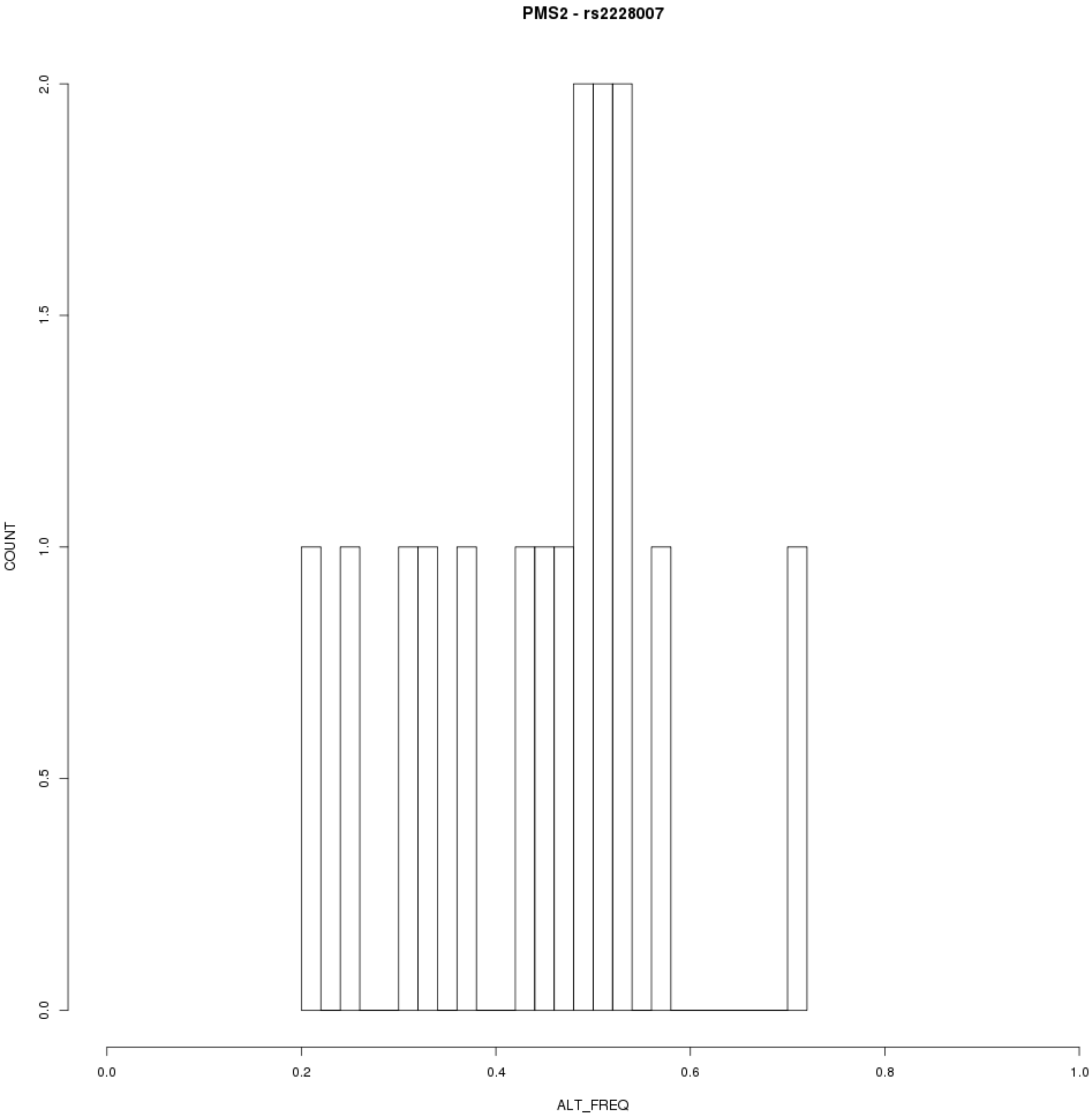

PMS2 - rs2228007
COUNT
ALT_FREQ
0.0
0.5
1.0
1.5
2.0
0.0
0.2
0.4
0.6
0.8
1.0

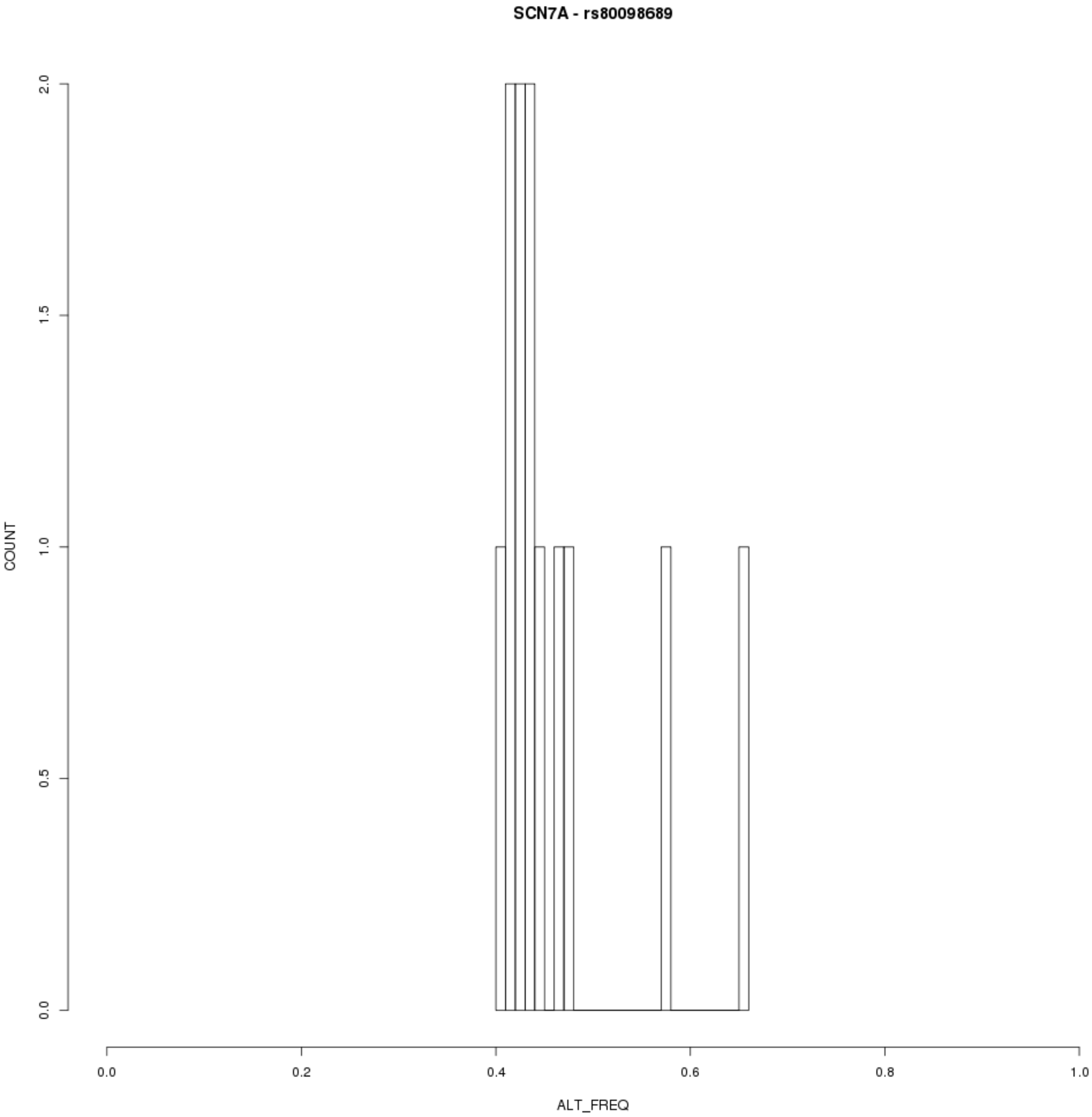
SCN7A - rs80098689
COUNT
0.0
0.5
1.0
1.5
2.0
ALT_FREQ
0.0
0.2
0.4
0.6
0.8
1.0

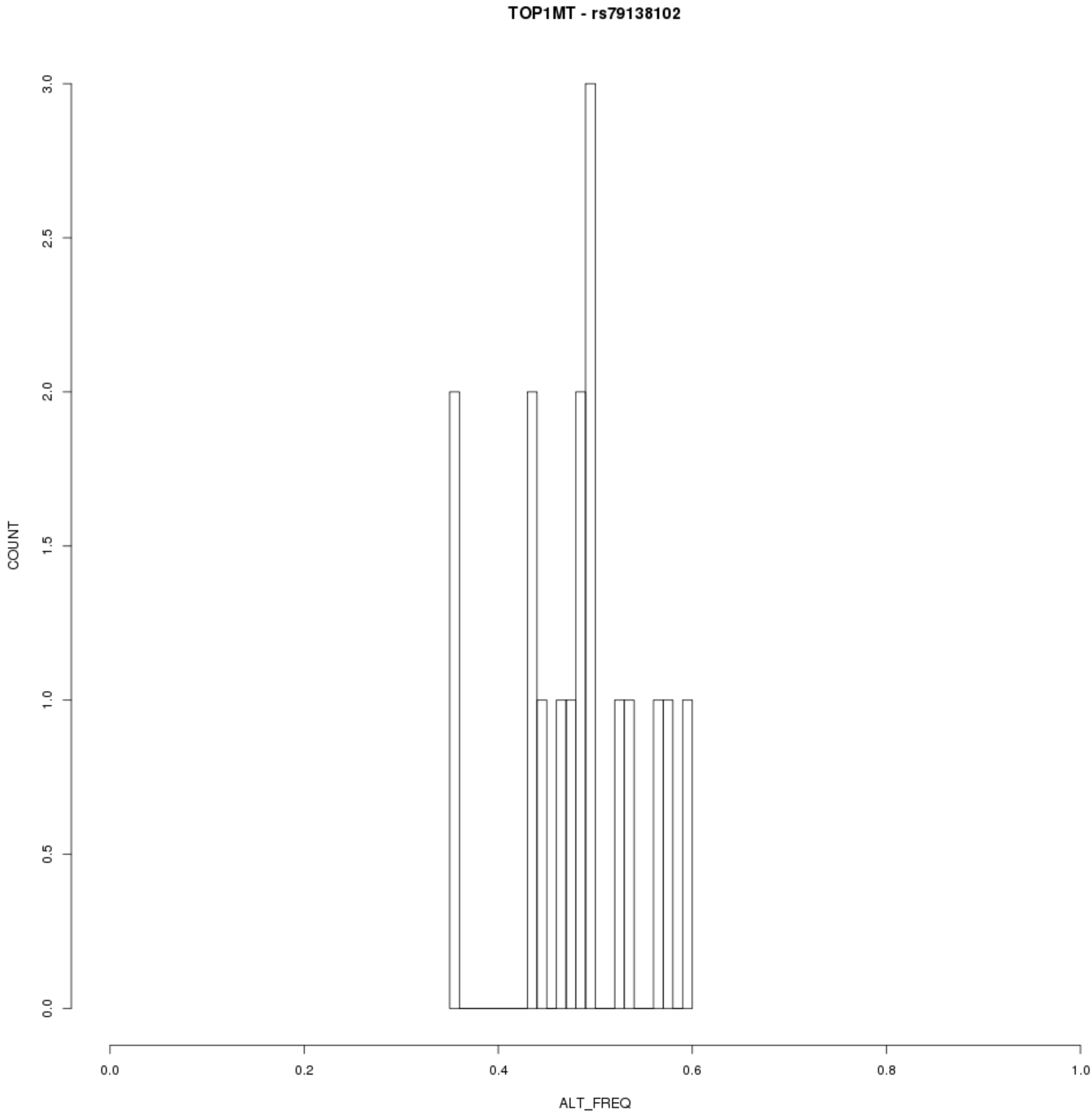
TOP1MT - rs79138102
COUNT
ALT_FREQ
0.0
0.5
1.0
1.5
2.0
2.5
3.0
0.0
0.2
0.4
0.6
0.8
1.0

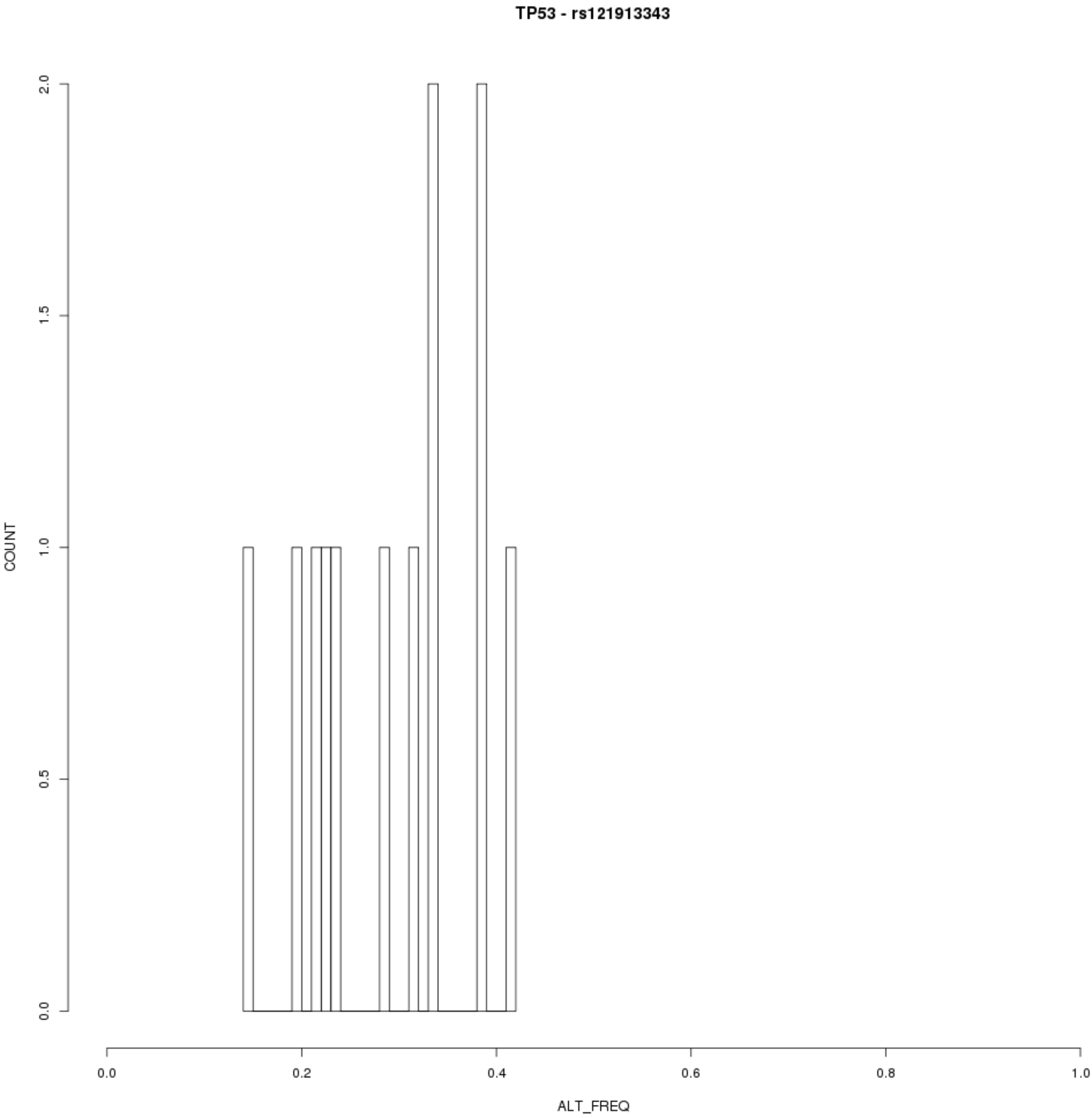

TP53 - rs121913343
COUNT
ALT_FREQ
0.0
0.5
1.0
1.5
2.0
0.0
0.2
0.4
0.6
0.8
1.0

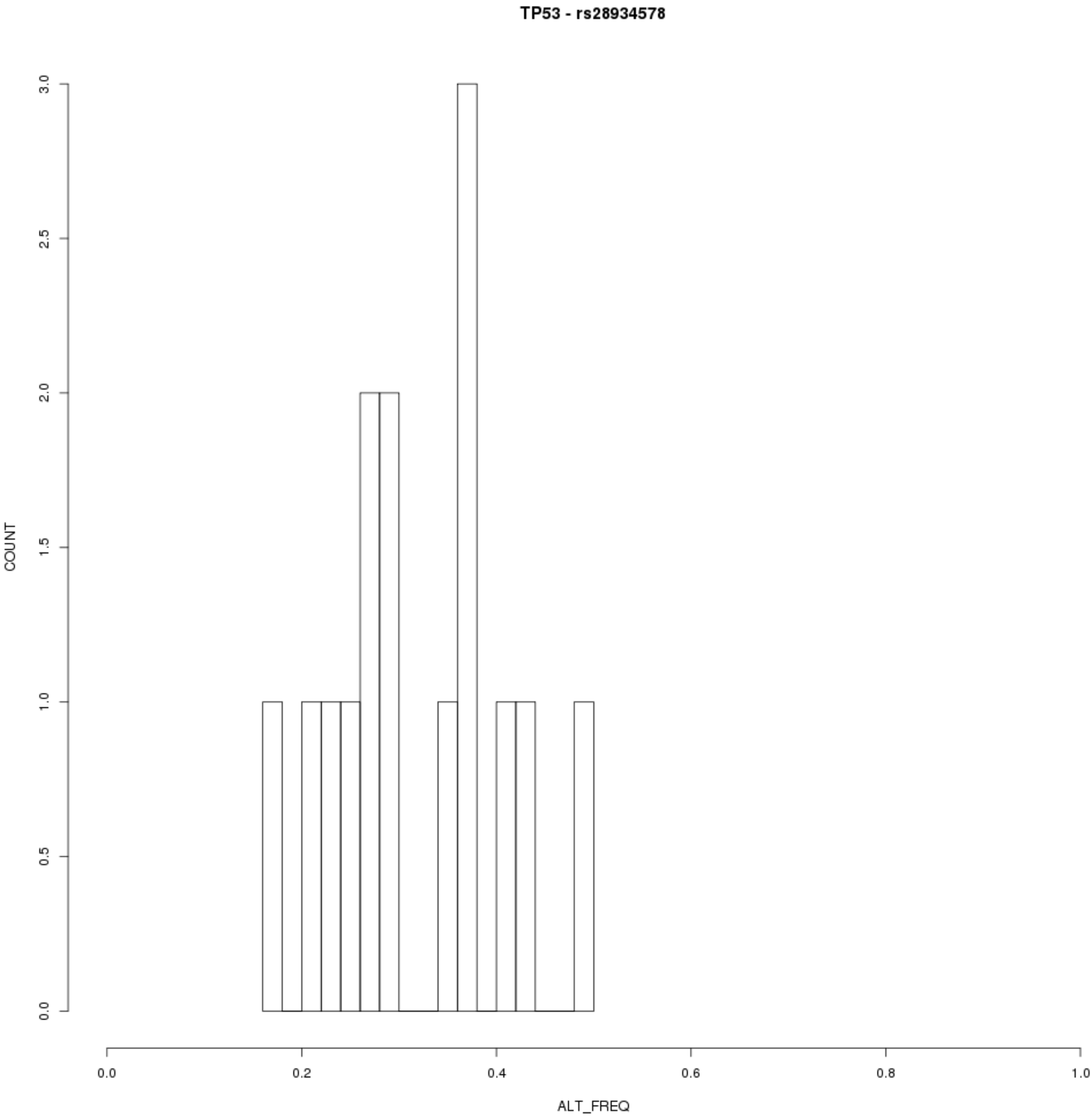

TP53 - rs28934578
COUNT
0.0
0.5
1.0
1.5
2.0
2.5
3.0
0.0
0.2
0.4
0.6
0.8
1.0
ALT_FREQ

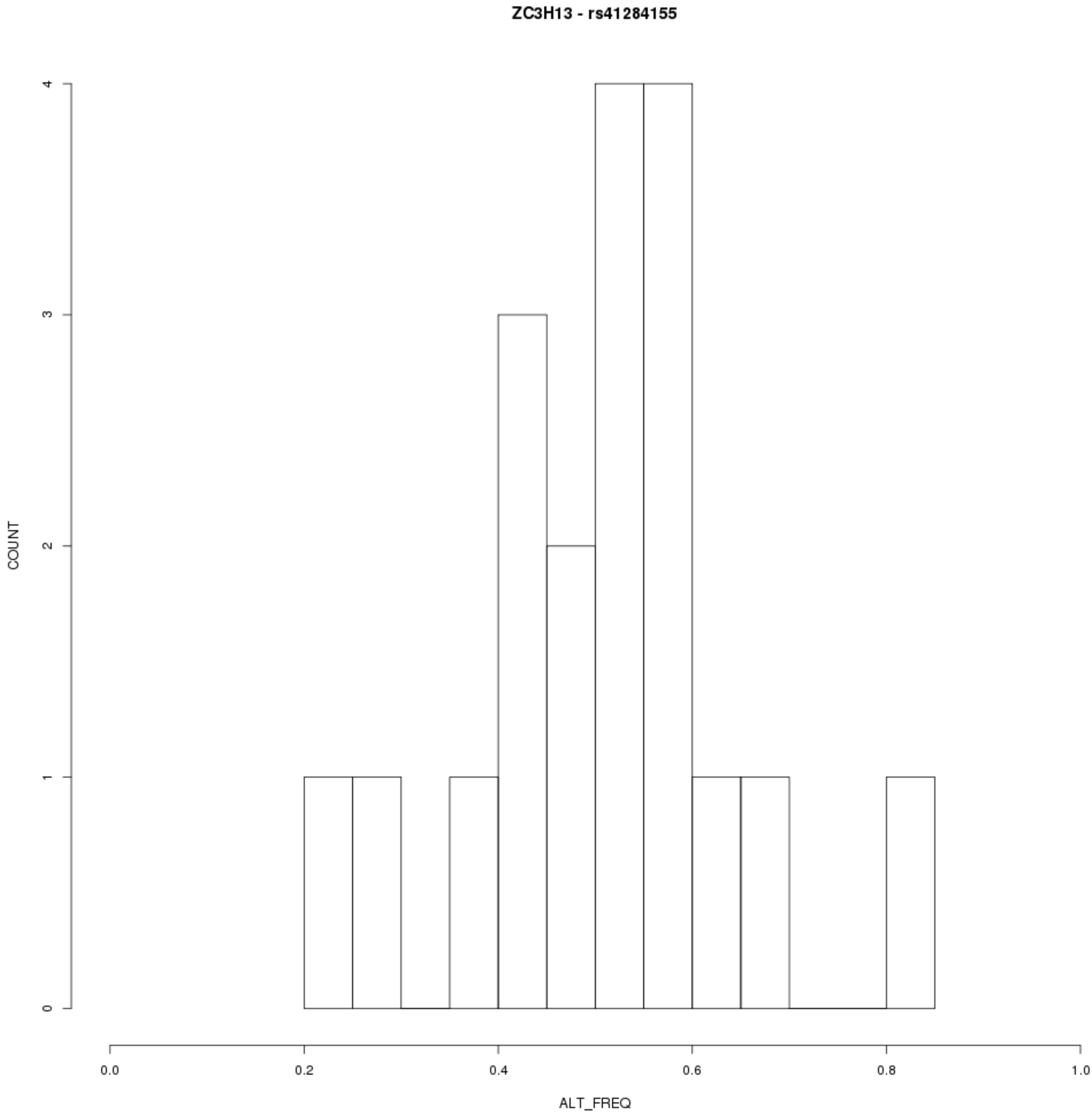
ZC3H13 - rs41284155
COUNT
ALT_FREQ
0
1
2
3
4
0.0
0.2
0.4
0.6
0.8
1.0
